# EPC1/2 regulate hematopoietic stem and progenitor cell proliferation by modulating H3 acetylation and DLST

**DOI:** 10.1016/j.isci.2024.109263

**Published:** 2024-02-17

**Authors:** WenYe Liu, Xi Liu, LingYa Li, ZhiPeng Tai, GuoLiang Li, Jing-Xia Liu

**Affiliations:** 1 College of Fisheries, Key Laboratory of Freshwater Animal Breeding, Ministry of Agriculture, Huazhong Agricultural University, Wuhan 430070, China; 2 College of Informatics, Agricultural Bioinformatics Key Laboratory of Hubei Province, Hubei Engineering Technology Research Center of Agricultural Big Data, Huazhong Agricultural University, Wuhan 430070, China

**Keywords:** Metabolomics, Animal nutrition, Laboratory animal science

## Abstract

Enhancers of polycomb 1 (EPC1) and 2 (EPC2) are involved in multiple biological processes as components of histone acetyltransferases/deacetylase complexes and transcriptional cofactors, and their dysfunction was associated with developmental defects and diseases. However, it remains unknown how their dysfunction induces hematopoietic stem and progenitor cell (HSPC) defects. Here, we show that depletion of *EPC1*/*2* significantly reduced the number of hematopoietic stem and progenitor cells (HSPCs) in the aorta-gonad mesonephros and caudal hematopoietic tissue regions by impairing HSPC proliferation, and consistently downregulated the expression of HSPC genes in K562 cells. This study demonstrates the functions of *EPC1*/*2* in regulating histone H3 acetylation, and in regulating DLST (dihydrolipoamide S-succinyltransferase) via H3 acetylation and cooperating with transcription factors serum response factor and FOXR2 together, and in the subsequent HSPC emergence and proliferation. Our results demonstrate the essential roles of *EPC1*/*2* in regulating H3 acetylation, and *DLST* as a linkage between EPC1 and EPC2 with mitochondria metabolism, in HSPC emergence and proliferation.

## Highlights

• EPC1 and EPC2 are indispensable for HSPC emergence and proliferation
• H3 acetylation and DLST are key regulators to mediate EPC1 and EPC2 effects on HSPCs

## Graphical abstract

**Figure:**
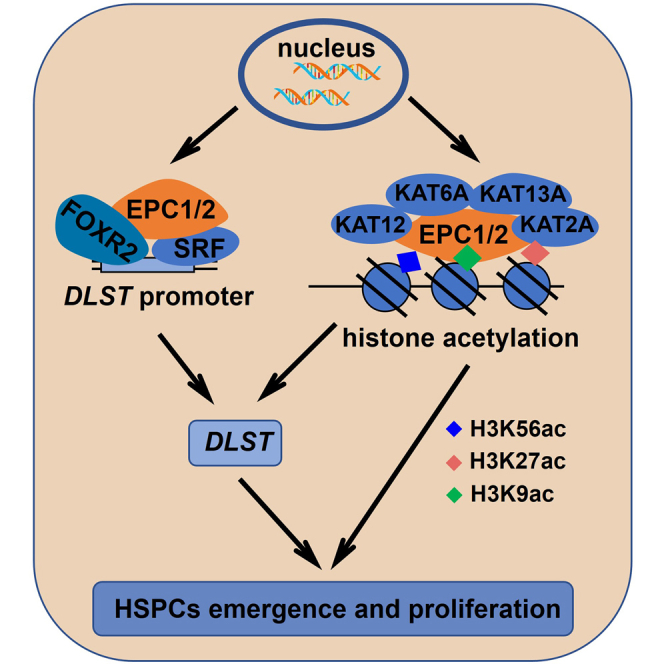

## Introduction

In vertebrates, hematopoietic stem and progenitor cells (HSPCs) are specified from hemogenic endothelium (HE) via the endothelial-hematopoietic transition (EHT) process in the aorta-gonad mesonephros (AGM) region, followed by amplification in the caudal hematopoietic tissue (CHT) region,^1^^,^^2^^,^^3^ and migration to the thymus and kidney to generate T cells and other lineages of blood cells (erythroid and myeloid lineage cells).^4^^,^^5^^,^^6^ Acetylation of histones and non-histones is involved in normal and malignant hematopoiesis by modulating chromatin status and adjusting the function of non-histone protein.^7^^,^^8^ Histone acetyltransferases (HATs) mainly include multiple families: MYST (MYST1 (KAT8), Tip60, MOZ (KAT6A), MORF, HBO1 (KAT7), MOF), p300/CBP, GNAT (PCAF, GCN5 (KAT2A), ELP3 (KAT9)), and nuclear (or steroid) receptor coactivator (NCOA1(KAT13A), NCOA2, NCOA3, and CLOCK),^8^^,^^9^ etc. Loss of CREB binding protein (CBP) function has been reported to cause defects in primitive hematopoiesis^10^ and disrupt HSPC development in mice.^11^^,^^12^ Lack of MOZ (KAT6A) HAT activity has been shown to cause defects in HSPCs^13^ and accelerate HSPC apoptosis.^14^

EPC1 and EPC2, as transcriptional cofactors or components of HATs/deacetylase complexes, have been found to be involved in a variety of biological processes, such as cell differentiation and metastasis.^15^^,^^16^^,^^17^ As transcriptional co-factors, EPC interact with RET finger protein (RFP) to repress gene transcription, and recruit homeodomain-only protein (HOP) or serum response factor (SRF) to induce skeletal muscle differentiation,^18^^,^^19^ and regulate transcriptional activity by interacting with E2F transcription factor 1 (E2F1).^17^ *EPC1* and *EPC2* are also related to acute myeloid leukemia (AML), which can directly or indirectly suppress the accumulation of MYC and apoptosis of AML cells, thereby maintaining oncogenic potential.^20^ Based on morpholino-mediated gene knockdown, a large-scale reverse genetic screen of 425 human chromatin factors in zebrafish found that EPC2 (as an epigenetic factor) is required for hematopoietic development, especially primitive erythropoiesis.^21^ However, how hematopoietic cells develop in zebrafish with deficiency of gene *EPC1* or *EPC2* and the related underlying mechanisms still remain unknown.

The purpose of this study was to identify the effects, potential mediators, and underlying mechanisms of *EPC1* and *EPC2* in hematopoietic development by a zebrafish genetic model with deletion of the genes *epc1a* or *epc2*, and human K562 cells with knockdown of functional *EPC1* or *EPC2.* We demonstrated that the functional deficiency of *EPC1* or *EPC2* led to impaired proliferation and reduced levels of acetylated H3 proteins in HSPCs. Importantly, we identified *DLST* and acetylated H3 proteins as downstream targets of *EPC1* and *EPC2* to positively regulate HSPC emergence and proliferation.

## Results

### *Epc1a* and *epc2* are indispensable for embryonic HSPC emergence

It has been reported that knockdown of *epc2* induce primitive hematopoiesis defects in zebrafish,^21^ while the hematopoiesis, especially the development of HSPCs, in embryos with either *epc1a* or *epc2* deficiency is still unknown. In this study, we found that zebrafish *epc1a* and *epc2* were ubiquitously and predominantly expressed in the brain and eyes, with expression in the intermediate cell mass (ICM) and AGM (Figure S1A), suggesting their potential involvement in zebrafish hematopoiesis. *Epc1a*^*−/−*^ (with an 11-bp deletion in exon 1), *epc2*^*−/−*^ (with a 4-bp deletion in exon 1), and *epc1a*^*−/−*^*epc2*^*−/−*^ mutants constructed in this study all exhibited mild developmental delay, with no obviously morphological defects observed during embryogenesis (Figures S1B and S1C). Compared with the control, the deletion of either *epc1a* or *epc2* induced significantly downregulated expression of the target gene in embryos (p < 0.01), respectively (Figure S1D), which was also verified by western blot (WB) (Figure S1E). Meanwhile, *epc1b* (homolog of *epc1a* and *epc2*) expression was significantly increased (p < 0.01) in both *epc1a*^*−/−*^ and *epc2*^*−/−*^ mutants (Figure S1D).

**Figure 1:**
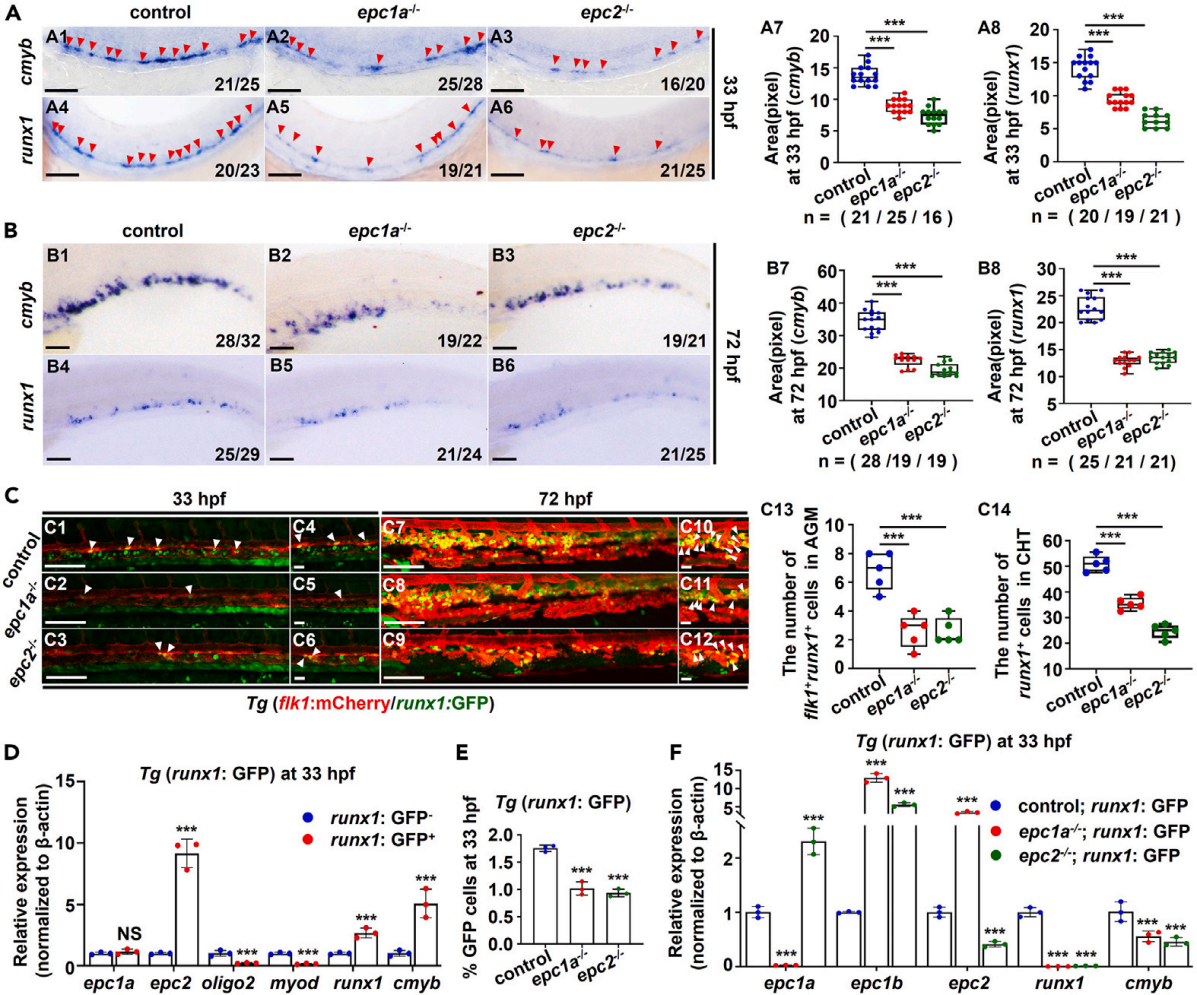
Loss of *epc1a* and *epc2* perturbed the development of hematopoietic stem and progenitor cells (HSPCs) in zebrafish (A and B) Expression of HSPC genes *cmyb* and *runx1* in the AGM (A1–A6) and CHT regions (B1–B6) in the control, *epc1a*^*−/−*^, and *epc2*^*−/−*^ embryos at 33 hpf and 72 hpf, and quantification of WISH data (A7 and A8, B7 and B8), with red arrowheads indicating positive signals. (C) Confocal images of the AGM (C1–C6) and CHT regions (C7–C12) in *Tg* (*flk1*: mCherry/*runx1*: GFP), *Tg* (*epc1a*^−/−^; *flk1*: mCherry/*runx1*: GFP), and *Tg* (*epc2*^−/−^; *flk1*: mCherry/*runx1*: GFP) embryos, with white arrowheads indicating double-positive cells. C4–C6 and C10–C12 present the magnified views of C1–C3 and C7–C12, respectively. (D) Expression of *epc1a*, *epc2*, *oligo2*, *myoD*, *runx1*, and *cmyb* in *runx1*-GFP^-^ (*runx1*^-^) and *runx1*-GFP^+^ (*runx1*^*+*^) cells sorted from *Tg* (*runx1*: GFP) at 33 hpf by One Step Cell-Direct qRT-PCR. (E) The percentage of *runx1*^*+*^ cells in *Tg* (*runx1*: GFP) (control), *Tg* (*epc1a*^−/−^; *runx1*: GFP), and *Tg* (*epc2*^−/−^; *runx1*: GFP) at 33 hpf, respectively. (F) Expression of *epc1a*, *epc1b*, *epc2*, *runx1*, and *cmyb* in *runx1*^+^ cells sorted from *Tg* (*runx1*: GFP) (control), *Tg* (*epc1a*^−/−^; *runx1*: GFP), and *Tg* (*epc2*^−/−^; *runx1*: GFP) at 33 hpf by One Step Cell-Direct qRT-PCR. Each experiment was repeated three times, and a representative result is shown. N_changed_/N_total_ in the right bottom corner of each panel indicates embryos with changed expression/total tested embryos, and n in calculation panels indicates the number of total tested embryos in each group. A1–A6, B1–B6, C1–C12, lateral view, anterior to the left, and dorsal to the up. Scale bars, 100 μm. Data are mean ± SD (n ≥ 3). t test, ∗p < 0.05, ∗∗p < 0.01, ∗∗∗p < 0.001; NS, not significant.

To explore whether *epc1a* and *epc2* deficiency affect the development of HSPCs in zebrafish, we detected the expression of HSPC markers *runx1*/*cmyb* in the mutants firstly. Whole-mount in situ hybridization (WISH) results displayed that compared with the control, *epc1a*^*−/−*^ and *epc2*^*−/−*^ mutants showed reduced expression (p < 0.001) of *runx1* and *cmyb* in the AGM at 33 hpf (Figure 1A) and in the CHT region at 72 hpf (Figure 1B), and of the T cell marker *rag1* in the thymus at 4 dpf (Figure S2A). Double knockout of *epc1a* and *epc2* resulted in a more severe reduction of *runx1*/*cmyb* and *rag1*, compared with their expression in either *epc1a*^*−/−*^ or *epc2*^*−/−*^ mutants (Figure S2B). Additionally, the numbers of *flk1*^+^*runx1*^*+*^ cells and *runx1*^*+*^ cells were significantly decreased in the AGM and CHT regions in the mutants at both 33 hpf and 72 hpf (Figure 1C). Similarly, antisense morpholinos targeting *epc1a* or *epc2*, respectively, led to the downregulated expressions (p < 0.001) of *cmyb* (Figure S3B) and *rag1* (Figures S3E1–S3E3) independent of *p53* (Figures S3G and S3H).^22^ Meanwhile, *epc2* knockdown significantly decreased (p < 0.001) the number of *flk1*^+^*runx1*^*+*^ cells in AGM at 33 hpf (Figure S3C), and both *epc1a* and *epc2* morphants showed an obvious decrease (p < 0.001) in the number of *runx1*^*+*^ cells in CHT at 72 hpf (Figure S3D), coupled with a decrease (p < 0.001) in *rag2*-positive fluorescence (*Tg* (*rag2*: dsRed)) (Figures S3E5–S3E10) and with growth retardation (Figure S3A). Collectively, *epc1a* and *epc2* deficiency might impair HSPC development in zebrafish.

In this study, in *runx1*^*+*^ cells sorted from *Tg* (*runx1*: GFP) embryos at 33 hpf,^23^ *runx1/cmyb* exhibited abundant expression (p < 0.001), in contrast to obvious low expression of the neural gene *olig2* and the muscle gene *myoD* (p < 0.001) (Figure 1D), suggesting the HSPC identity of *runx1*^*+*^ cells. The abundant expression of *epc2* and approximate expression of *epc1a* were observed in *runx1*^*+*^ cells (Figure 1D), further supporting their potential involvement in HSPC development. Depletion of either *epc1a* or *epc2* markedly decreased (p < 0.001) the percentage of *runx1*^*+*^ cells relative to the control group (Figure 1E). However, in *Tg* (*epc1a*^−/−^; *runx1*: GFP), *epc1a* exhibited significantly reduced expression (p < 0.001) while *epc2* increased in *runx1*^*+*^ cells and vice versa, and *epc1b* expression was markedly upregulated in *epc1a-* or *epc2-*deficient *runx1*^*+*^ cells (Figure 1F). Meanwhile, the ectopic expression of *epc1* mRNA not only restored the decrease of *runx1/cmyb* in the AGM region at 33 hpf in *epc1a*^−/−^ embryos, but also partially restored the decrease of *runx1/cmyb* in *epc2*^−/−^ embryos, and vice versa (Figure S4), suggesting there might be no off-target effects occurred in each of mutants and that *epc1a* and *epc2* may function redundantly during HSPC development.

Compared with the control, *epc1a*^*−/−*^ and *epc2*^*−/−*^ mutants showed no change in the expression of lateral mesoderm maker *pax2a* at 10 hpf (Figures S5A1–S5A4) but a decrease at 12 hpf (Figures S5A5–S5A8), and they also exhibited no change in the expression of ectoderm maker *six3b* (Figure S5B), trunk mesoderm maker *myoD* (Figure S5C) and endoderm maker *gata5* (Figure S5D). In addition*, fli1/flk1* marking the vessels displayed normal expression in *epc1a*^*−/−*^ and *epc2*^*−/−*^ mutants (Figure S5E), suggesting that *epc1a* and *epc2* may function specifically in hematopoiesis.

### *Epc1a* and *epc2* deficiency inhibit HSPC proliferation

**Figure 2:**
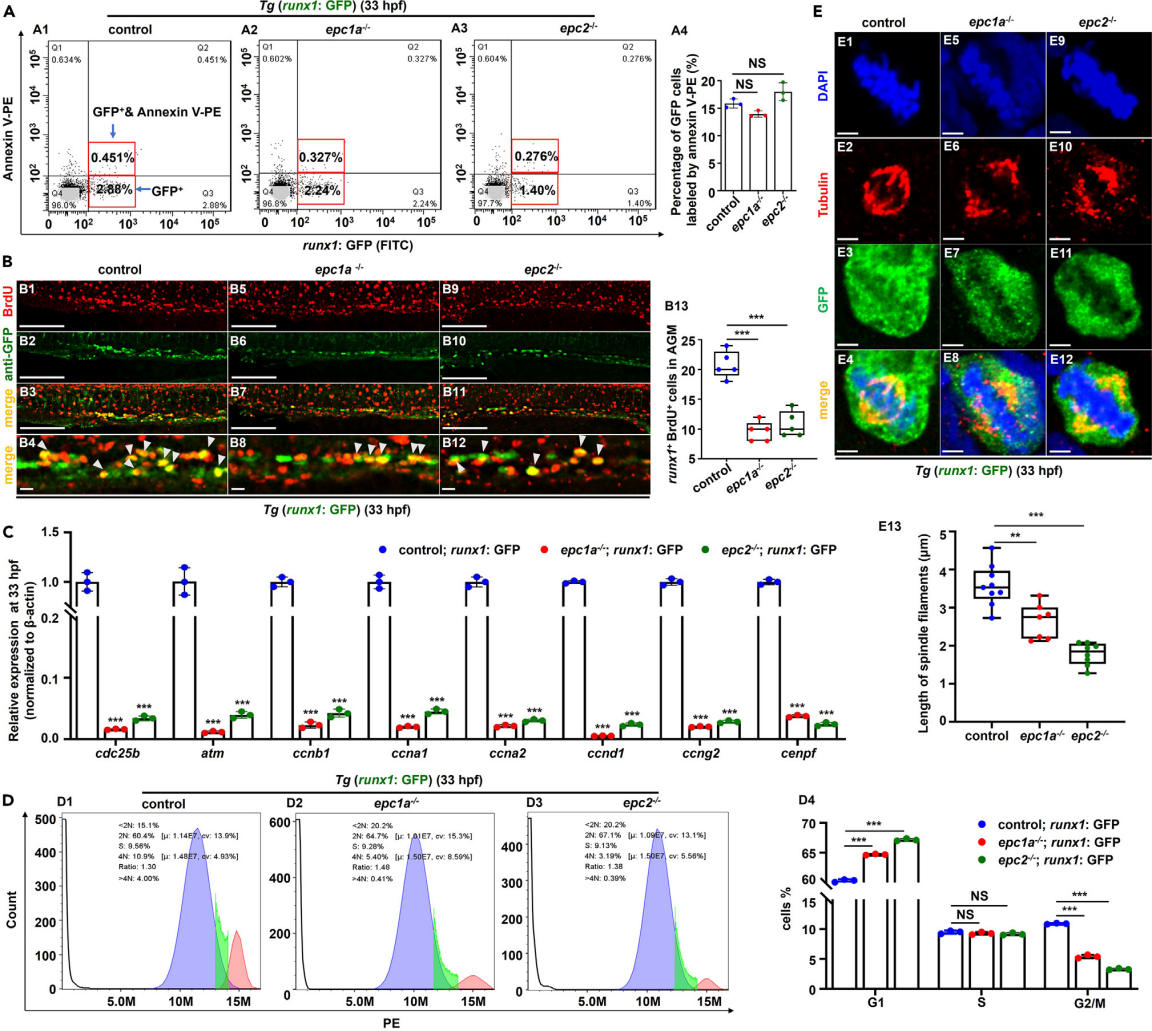
Effects of *epc1a* and *epc2* deficiency on HSPC proliferation and apoptosis (A) The apoptosis of *runx1*^+^ cells in *Tg* (*runx1*: GFP), *Tg* (*epc1a*^−/−^; *runx1*: GFP), and *Tg* (*epc2*^−/−^; *runx1*: GFP) embryos at 33 hpf (A1–A3), with GFP-positive cells selected for the analysis of apoptotic cells labeled by annexinV-PE, as well as percentages of GFP-positive cells labeled by annexinV-PE (A4). (B) Double staining of *runx1*^*+*^(GFP) and BrdU in the AGM in the control, *epc1a*^−/−^ and *epc2*^−/−^ embryos at 33 hpf (B1–B12), and quantification of *runx1*^+^BrdU^+^ cells (B13), with white arrowheads indicating double-positive cells. B4, B8, and B12 present the magnified views of B3, B7, and B11, respectively. (C) Expression of the cell cycle-related genes in *runx1*^+^ cells sorted from *Tg* (*runx1*: GFP) (control), *Tg* (*epc1a*^−/−^; *runx1*: GFP), and *Tg* (*epc2*^−/−^; *runx1*: GFP) at 33 hpf. (D) Cell cycle analysis in *runx1*-positive cells sorted from *Tg* (*runx1*: GFP) (control), *Tg* (*epc1a*^−/−^; *runx1*: GFP), and *Tg* (*epc2*^−/−^; *runx1*: GFP) at 33 hpf (D1–D3), and quantification analysis of the percentage of cells in G1, S and G2/M phases (D4). (E) Double staining of *runx1*^+^ (GFP) and α-Tubulin in the AGM region in the control, *epc1a*^−/−^ and *epc2*^−/−^ embryos at 33 hpf (E1–E12), and quantification of length of spindle filaments (E13). Each experiment was repeated three times, and a representative result is shown. B1–B12, lateral view, anterior to the left, and dorsal to the up. Scale bars, 100 μm (B1–B12) and 2 μm (E1–E24), Data are presented as mean ± SD (n ≥ 3). t test, ∗p < 0.05, ∗∗p < 0.01, ∗∗∗p < 0.001, NS, not significant.

The alteration in cell proliferation or apoptosis^23^^,^^24^^,^^25^ might contribute to HSPC defects in the mutants. In Figure 2A, compared with the control, *epc1a*^*−/−*^ or *epc2*^*−/−*^ mutants showed no significant change in HSPC apoptosis, but marked reduction (p < 0.001) in the number of *runx1*^*+*^BrdU^*+*^ double-positive cells in the AGM (Figure 2B) and CHT regions (Figure S6A). The expression of cell cycle-related genes displayed obvious changes in *epc1a*^−/−^ embryos based on RNA-seq analysis (Figure S6F; Table S12). Compared with the control, *epc1a*^−/−^ and *epc2*^−/−^ embryos showed a significant decrease in the expression of cell cycle-related genes (p < 0.001), such as *ccna1*, *ccnb1*, and *ccng2* (Figure 2C) and a lower percentage of *runx1*^+^ HSPCs (p < 0.01) at the G2/M phase, while an increase (p < 0.05) at the G1 stage at 33 hpf (Figure 2D) and 72 hpf (Figure S6B). Additionally, *epc1a*^−/−^ and *epc2*^−/−^ embryos exhibited abnormal spindle structure in metaphase of GFP^+^ cells (*runx1*^+^ HSPC cells) (Figures 2E and S6C) and GFP^−^ cells (Figures S7D and S7E) in the AGM and CHT regions by immunofluorescent staining using an anti-α-tubulin (labeling mitotic spindle) antibody. Collectively, HSPC defects in *epc1a*^*−/−*^ or *epc2*^*−/−*^ mutants may be attributed to the impaired proliferation caused by disturbing HSPC cell cycle in zebrafish.

### Histone H3 acetylation and *dlst* are key regulators for *epc1a* and *epc2* modulation of HSPC emergence and proliferation

**Figure 3:**
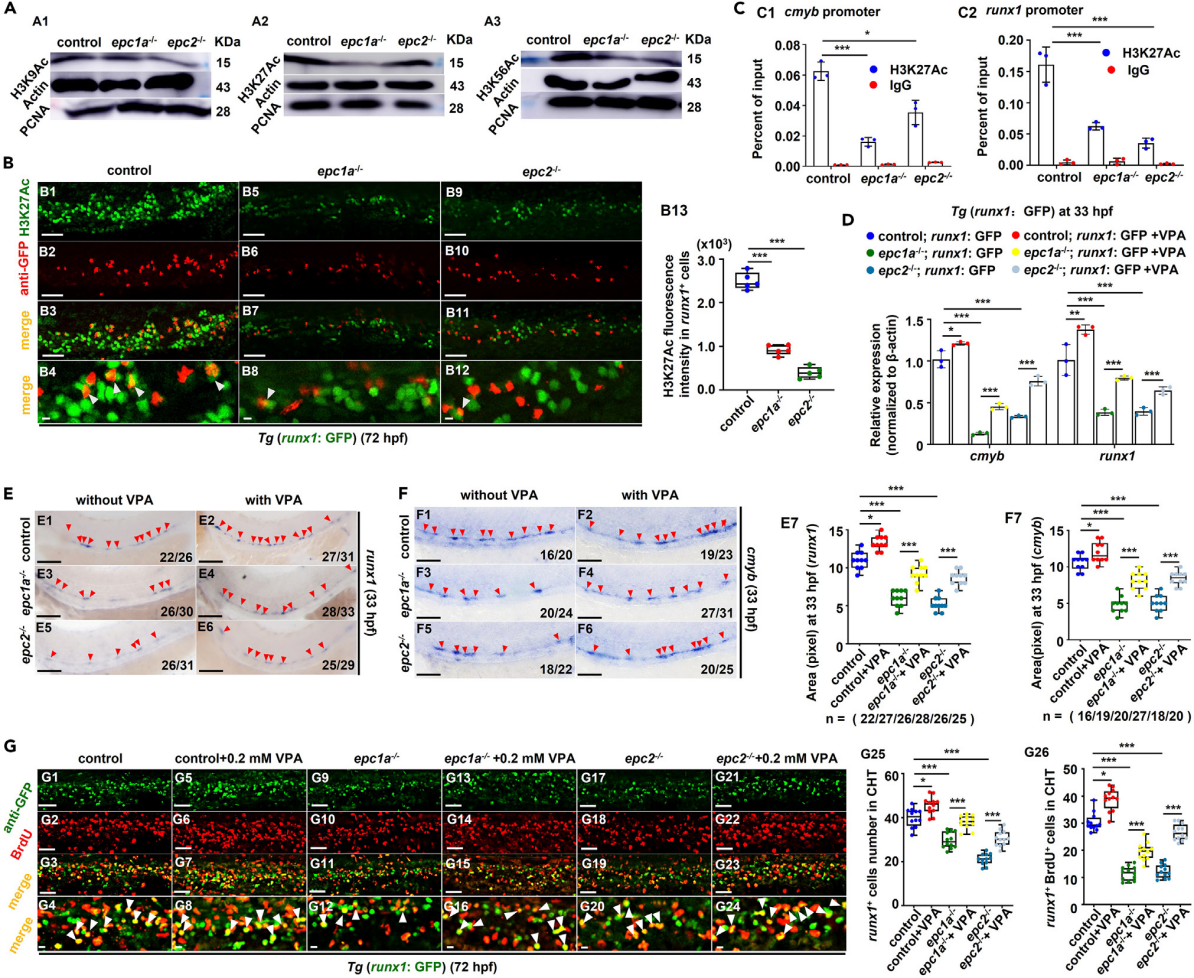
Deletion of *epc1a* and *epc2* induced impairment in HSPC proliferation by downregulating histone acetylation level (A) Western blotting analysis of H3K9Ac (A1), H3K27Ac (A2), and H3K56Ac (A3) in the control, *epc1a*^*−/−*^, and *epc2*^*−/−*^ embryos, with actin and PCNA as the internal control, and quantification of H3K9Ac (A4), H3K27Ac (A5), and H3K56Ac (**A6**). (B) Double staining of *runx1*^+^ (GFP) with H3K27Ac in the CHT region in the control, *epc1a*^−/−^ and *epc2*^−/−^ embryos at 72 hpf (B1–B12), and quantification of H3K27Ac fluorescence intensity in *runx1*^+^ cells (B13), with white arrowheads indicating double-positive cells. B4, B8, and B12 present the magnified images of B3, B7, and B11, respectively. (C) ChIP-qPCR analysis of H3K27Ac occupancy at promoters of *cmyb* (C1) and *runx1* (C2) in the control, *epc1a*^−/−^, and *epc2*^−/−^ embryos. The occupancy was presented as the percentage of input. (D) qRT-PCR analysis of *runx1* and *cmyb* in *runx1*^+^ cells sorted from *Tg* (*runx1*: GFP) (control), *Tg* (*epc1a*^−/−^; *runx1*: GFP), and *Tg* (*epc2*^−/−^; *runx1*: GFP), and the corresponding groups treated with 0.2 mM VPA at 33 hpf. (E and F) WISH analysis of *runx1* (E1–E6) and *cmyb* (F1–F6) in the control, *epc1a*^*−/−*^, and *epc2*^*−/−*^ embryos and the corresponding groups treated with VPA in the AGM region at 33 hpf, with red arrowheads indicating positive signals, and quantification of the WISH data (E7 and F7). (G) Double staining of *runx1*^+^(GFP) and BrdU in the CHT region in the control, *epc1a*^−/−^ and *epc2*^−/−^ embryos and the corresponding groups treated with VPA at 72 hpf (G1–G24), and quantification of *runx1*^+^ cells (G25) and *runx1*^+^BrdU^+^ cells (G26), with white arrowheads indicating double-positive cells. G4, G8, G12, G16, G20, and G24 show the magnified images for better visualization. Each experiment was repeated three times, and a representative result is shown. N_changed_/N_total_ in the right bottom corner of each panel indicates embryos with changed expression/total tested embryos, and n in calculation panels indicates the number of total tested embryos in each group. B1–B12, E1–E6, F1–F6 and G1–G24, lateral view, anterior to the left, and dorsal to the up. Scale bars,100 μm. Data are presented as mean ± SD (n ≥ 3). t test, ∗p < 0.05, ∗∗p < 0.01, ∗∗∗p < 0.001, NS, not significant.

As epigenetic factors, *EPC1* and *EPC2* have been found to play important roles in the histone acetylation/deacetylation process,^26^^,^^27^^,^^28^ and involve in zebrafish primitive erythropoiesis via NuA4 or HDAC-NuRD complexes.^21^ In this study, acetylation-related Gene Ontology (GO) terms, such as internal protein amino acid acetylation, histone acetyltransferase complex, and histone H4 acetylation, were seen to be significantly enriched in *epc1a*^*−/−*^ mutants versus the control (Figure S7A; Table S13). The levels of H3K9Ac, H3K27Ac, H3K56Ac (Figure 3A; Figure S7B), and H4K5/K8/K12/K16Ac (Figure S7C) were clearly downregulated (p < 0.01), while H3K4Me1 protein level showed no change (Figure S7D) and H3K27Me3 increased (Figure S7E), with loss of functional *epc1a* or *epc2* relative to the control group. The fluorescence intensity of H3K9Ac, H3K27Ac, and H3K56Ac in *runx1*^+^ HSPC cells showed a marked decrease (p < 0.001), and the number of *runx1*^+^ cells was decreased significantly in the mutants versus the control (Figures 3B, S7F, and S7G). Meanwhile, depletion of *epc1a* or *epc2* in zebrafish caused significant reduction (p < 0.05) of H3K27Ac (as a marker of active gene promoters and enhancers)^29^ enrichment on promoters of *cmyb* (Figure 3C1) and *runx1* (Figure 3C2).

Valproic acid (VPA, an inhibitor of the deacetylase activity of HDACs *in vitro* and *in vivo*^30^^,^^31^) treatment from 11 hpf was seen to effectively rescue global H3K27Ac level (Figure S7H) and *cmyb*/*runx1* expression in *epc1a-* or *epc2-*deficient embryos and in *runx1*^+^ HSPCs (p < 0.01) (Figures 3D–3F). Importantly, VPA treatment resulted in an efficient rescue (p < 0.001) of *runx1*^*+*^ cells and *runx1*^*+*^BrdU^*+*^ cells in CHT region in *epc1a*^*−/−*^ and *epc2*^*−/−*^ mutants (Figure 3G), suggesting that *epc1a* and *epc2* may regulate HSPC emergence and proliferation by facilitating histone H3 acetylation.

**Figure 4:**
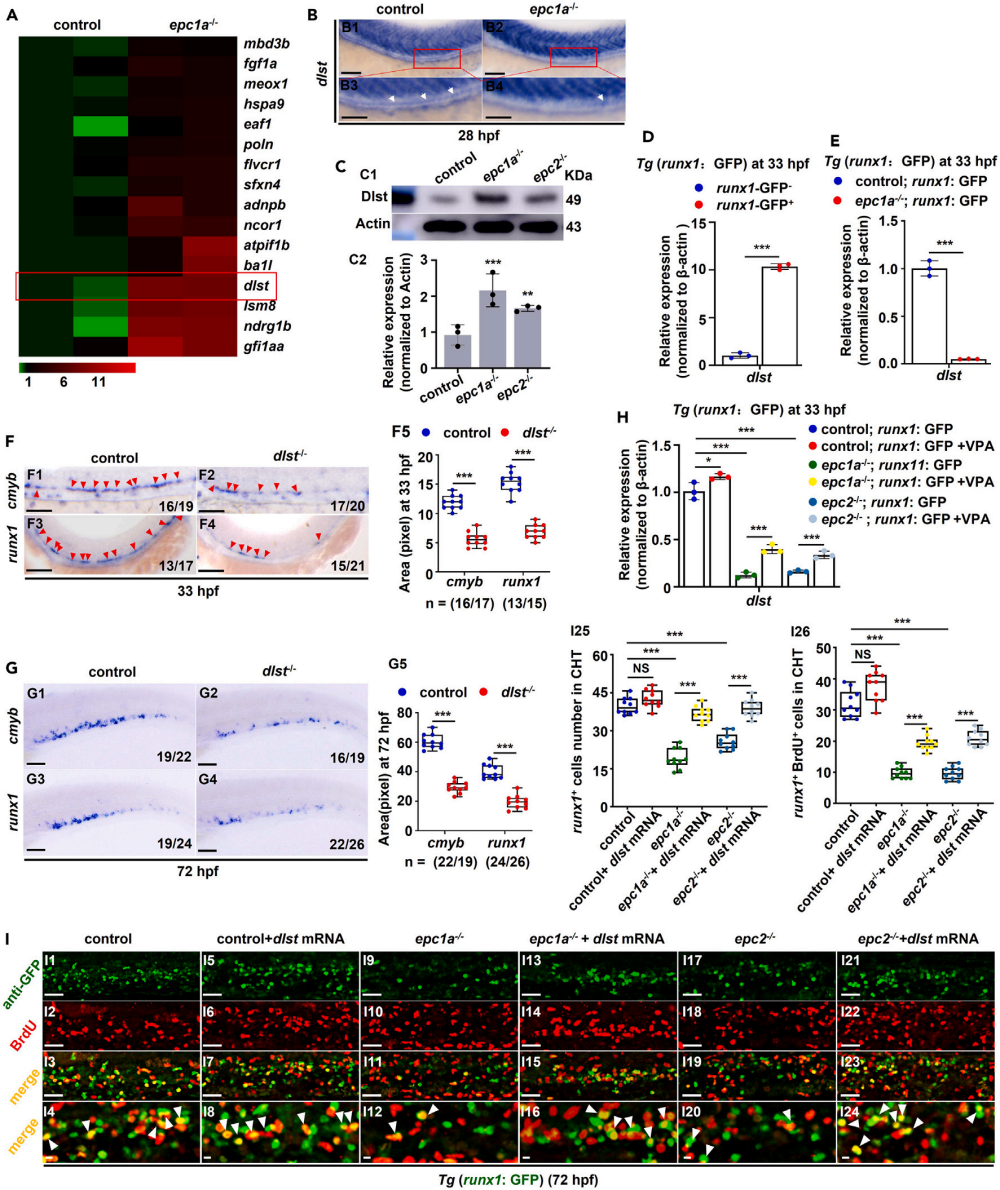
*Dlst* acts downstream of *epc1a* and *epc2* in regulating HSPC emergence and proliferation (A) Heatmap for upregulated DEGs related to hematopoiesis in the control and *epc1a*^−/−^ embryos based on RNA-seq data, with red box indicating the *dlst* expression. (B) WISH analysis of *dlst* expression in the control and *epc1a*^−/−^ embryos, with white arrowheads indicating AGM region. (C) Protein level of Dlst in the control, *epc1a*^−/−^ and *epc2*^−/−^ embryos, with actin as the internal control (C1), and quantification analysis (C2). (D) Expression of *dlst* in *runx1*GFP^−^ (*runx1*^-^) and *runx1*GFP^+^ (*runx1*^+^) cells at 33 hpf. (E) Expression of *dlst* in *runx1*^+^ cells in the control and *epc1a*^−/−^ embryos. (F and G) WISH analysis of *cmyb* and *runx1* in the control and *dlst*^*−/−*^ embryos in the AGM region at 33 hpf (F1–F4) (with red arrowheads indicating positive signals) and in the CHT region at 72 hpf (G1–G4), respectively, and quantification of the WISH data (F5 and G5), with arrowheads indicating positive signals. (H) Expression of *dlst* in *runx1*^+^ (GFP) cells sorted from *Tg* (*runx1*: GFP), *Tg* (*epc1a*^−/−^; *runx1*: GFP), and *Tg* (*epc2*^−/−^; *runx1*: GFP), and the corresponding groups treated with VPA. (I) Double staining of *runx1*^+^ and BrdU in the CHT region in the control, *epc1a*^−/−^, and *epc2*^−/−^ embryos and the corresponding groups injected with *dlst* mRNA at 72 hpf (I1–I24), and quantification of *runx1*^+^ cells (I25) and *runx1*^+^BrdU^+^ cells (I26), with white arrowheads indicating double-positive cells. I4, I8, I12, I16, I20, and I24 show the magnified images for I3, I7, I11, I15, I19, and I23, respectively. Each experiment was repeated three times, and a representative result is shown. N_changed_/N_total_ in the right bottom corner of each panel indicates embryos with changed expression/total tested embryos, and n in calculation panels indicates the number of total tested embryos in each group. All embryos are shown in lateral view, anterior to the left, and dorsal to the up. Scale bars, 100 μm. Data are presented as mean ± SD (n ≥ 3). t test, ∗p < 0.05, ∗∗p < 0.01, ∗∗∗p < 0.001, NS, not significant.

In order to comprehensively understand the potential other roles of *epc1a* or *epc2* during zebrafish HSPC development, we further analyzed differentially expressed genes (DEGs) related to hematopoiesis in *epc1a*^*−/−*^ mutant based on RNA-seq data (Table S12). Compared with the control, *epc1a*^*−/−*^ mutant showed obvious changes (p < 0.001) in the expression of a series of hematopoiesis-related genes, such as *dlst*, *nap1l4a*, *mbd3b*, *kat8*, and *ncor1* (Figure 4A; Figure S8A), which were further confirmed by WISH analysis (Figure S8B). Among them, *dlst* displayed reduced expression specifically in the AGM region in *epc1a*^−/−^ embryos, but with increased expression elsewhere in the whole embryos (Figure 4B), which was also verified by WB (Figure 4C). Meanwhile, *dlst* was expressed abundantly in *runx1*^*+*^ cells (Figure 4D), but downregulated significantly (p < 0.001) in *runx1*^*+*^ HSPC cells sorted from either *Tg* (*epc1a*^−/−^; *runx1*: GFP) (Figure 4E) or *Tg* (*epc2*^−/−^; *runx1*: GFP) embryos (Figure S9A).

DLST (dihydrolipoamide S-succinyltransferase), a tricarboxylic acid (TCA) cycle enzyme, is an important mediator of MYC-driven leukemogenesis,^32^ and promotes tumor aggression in neuroblastoma.^33^ Meanwhile, the TCA cycle is one of the constituents of metabolome which has been reported function pivotally in HSPC emergence^34^ and differentiation.^35^^,^^36^ In this study, we found the HSPC-specific downregulated expression of *dlst*, which might be the potential attributor in HSPC defects occurred in *epc1a*^−/−^or *epc2*^−/−^ mutants. In this study, zebrafish *dlst* was ubiquitously and predominantly expressed in muscle during early embryogenesis (Figure S9B). *Dlst* morphants exhibited obvious reduction (p < 0.001) in the expressions of *runx1/cmyb* (Figures S9C and S9D) and *rag1* (Figure S9E), and decreased number of *flk1*^*+*^*runx1*^*+*^ and *runx1*^*+*^ cells in the AGM and CHT regions (Figure S9F), independent of *p53* function (Figures S9G–S9I). Consistently, the robustly reduced expression of *runx1/cmyb* in the AGM and CHT regions was further confirmed in *dlst*^*−/−*^ mutants (with an 18 bp insertion and 3 bp substitution in the coding region of exon 8) (Figures S10A–S10C; Figures 4F and 4G), and *dlst* mRNA ectopic expression partially recovered the decrease of *runx1/cmyb* in *dlst*^*−/−*^ embryos at 33 hpf (Figure S10D). VPA was seen to significantly rescue (p < 0.001) the expression of *dlst* mRNA (Figure 4H) and the fluorescence intensity of Dlst protein (Figure S11) in *runx1*^*+*^ HSPC cells in either *epc1a*^*−/−*^ or *epc2*^*−/−*^ mutant. Meanwhile, the decreased *runx1/cmyb* expression in the mutants were efficiently rescued in both the AGM region and the CHT region (p < 0.01 and p < 0.001, respectively) by ectopic expression of *dlst* mRNA (Figure S12; Figure 4I), suggesting that *epc1a* and *epc2* may also regulate HSPC emergence and proliferation by modulating *dlst* specifically in HSPCs, which was also partially regulated by histone H3 acetylation.

### Roles of *EPC1* and *EPC2* are conserved in human hematopoietic cells

In a previous study, *EPC1* and *EPC2* were shown to play critical roles in sustaining AML stem cell potential.^20^ Here, we inactivated *EPC1* and *EPC2* in K562 cells (K562 line is derived from a chronic myeloid leukemia/erythroleukemia patient,^37^^,^^38^ and commonly used as an *in vitro* model to study hematopoietic development)^23^^,^^39^ By lentivirus-mediated shRNA knockdown^40^ in constructing shEPC1 or shEPC2 functional deficiency K562 cells, effective targets were screened by enrichment score based on qRT-PCR of the transcript level of *hEPC1* or *hEPC2*. Compared with the control, ShEPC1-2 targeting *EPC1* (abbreviated as shEPC1 in the following) and ShEPC2-3 targeting *EPC2* (abbreviated as shEPC2) exhibited stable and obvious knockdown activities (p < 0.001) (Figures S13A and S13B), and K562 cells with stable expression of ShEPC1-2 (shEPC1) and of ShEPC2-3 (shEPC2), respectively, were selected and maintained as stable knockdown cell lines in the following tests. Both *EPC1* and *EPC2* were seen to be significantly downregulated (p < 0.001) in ShEPC1/2 (both knockdown of *EPC1* and *EPC1* via both ShEPC1-2 and ShEPC2-3 in K562 cells) stable cell lines (Figure S13C). Cell proliferation in K562 cells was significantly weakened (p < 0.001) by knockdown of either *EPC1* or *EPC2* versus the control (Figure S13D), consistent with the tendency in *epc1a* or *epc2* mutant. Meanwhile, *EPC1* or *EPC2* knockdown induced a marked increase (p < 0.001) in cell apoptosis (Figure S13E). These data indicated that human erythroleukemia cells may depend on EPC1 and EPC2 for proliferation and anti-apoptosis defense.

**Figure 5:**
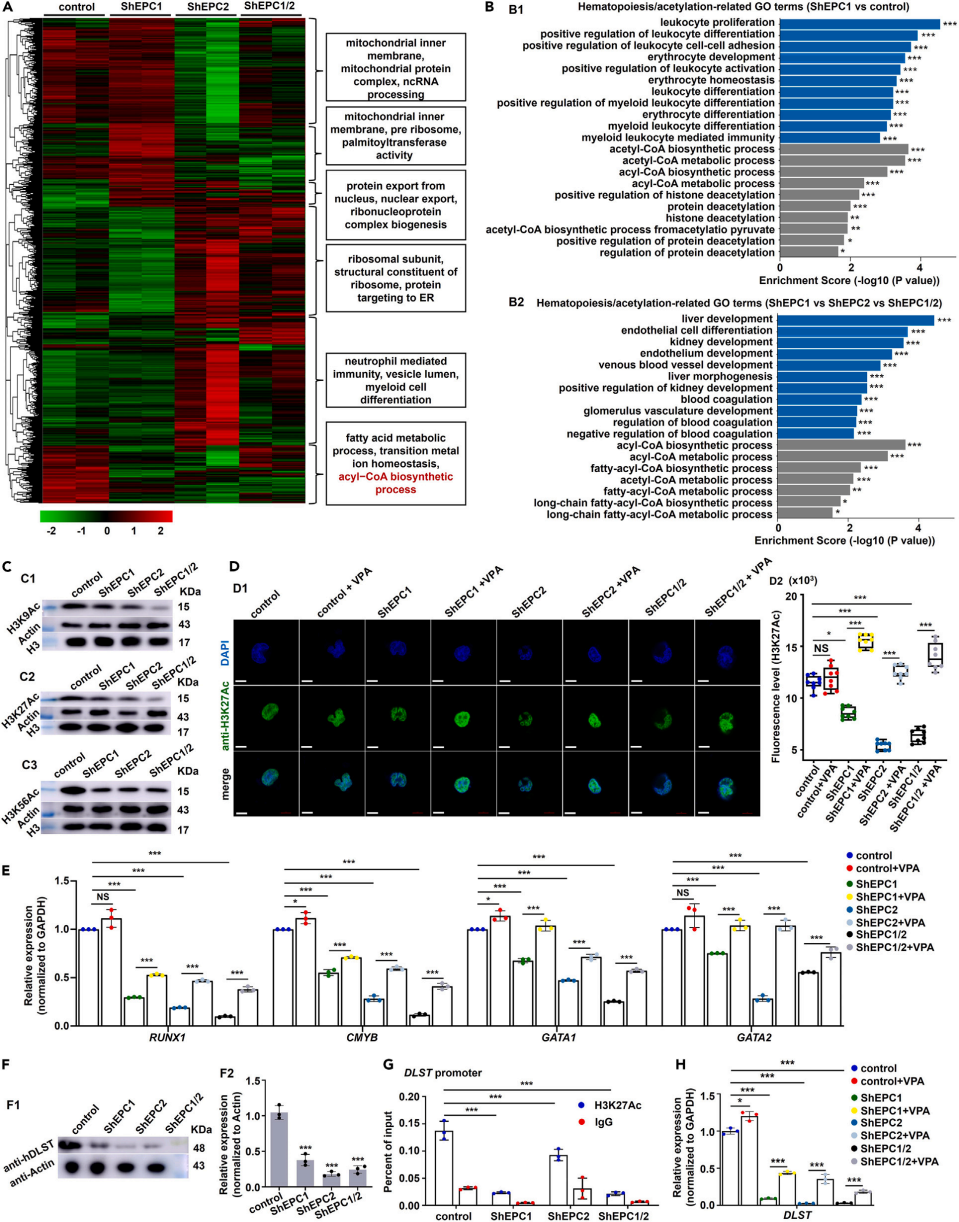
Roles of EPC1 and EPC2 are conserved in human hematopoietic cells (A) Heatmap for the DEGs involved in different biological processes based on RNA-seq data of control, ShEPC1, ShEPC2, and ShEPC1/2 K562 cells. (B) GO analysis of DEGs involved in acetylation and hematopoiesis in control and ShEPC1 K562 cells (B1), and in control, ShEPC1, ShEPC2, and ShEPC1/2 K562 cells (B2), respectively, based on RNA-seq data. (C) Western blotting analysis of the protein levels of H3K9Ac (C1), H3K27Ac (C2) and H3K56Ac (C3) in control, ShEPC1, ShEPC2, and ShEPC1/2 cells, with actin and PCNA as the internal controls. (D) Immunofluorescence analysis of the control, ShEPC1, ShEPC2, and ShEPC1/2 K562 cells and corresponding groups treated with VPA. (D1) using the anti-H3K27Ac antibody, and quantification of fluorescence intensity (D2). (E) qRT-PCR expression analysis of *RUNX1*, *CMYB*, *GATA1*, and *GATA2* in control, ShEPC1, ShEPC2, and ShEPC1/2 cells as well as the corresponding groups treated with VPA. (F) Western blotting analysis of the protein level of DLST in control, ShEPC1, ShEPC2, and ShEPC1/2 cells, with actin as the internal controls (F1), and quantification results (F2). (G) *DLST* expression in control, ShEPC1, ShEPC2, and ShEPC1/2 cells as well as corresponding groups treated with VPA. (H) ChIP-qPCR analysis of H3K27Ac occupancy at *DLST* promoters in the control, ShEPC1, ShEPC2, and ShEPC1/2 cells. The occupancy was presented as the percentage of input. Each experiment was repeated three times, and a representative result is shown. Scale bars, 5 μm, Data are presented as mean ± SD (n ≥ 3). t test, ∗p < 0.05, ∗∗p < 0.01, ∗∗∗p < 0.001, NS, not significant.

The expressions of most hematopoietic genes were clearly reduced (p < 0.001) upon either *EPC1* or *EPC2* knockdown relative to the control (Figures S13F and S13G). The transcription profiles were further analyzed by RNA-seq upon knockdown of either *hEPC1*, *hEPC2*, or *hEPC1/2*. ShEPC1, ShEPC2, and ShEPC1/2 cells were seen to be downregulated in the expression of genes related to fatty acid metabolic process, transition metal ion homeostasis, and acyl-CoA biosynthetic process (Figure 5A; Table S13), and ShEPC1, ShEPC2, and ShEPC1/2 cells showed remarkable enrichment (p < 0.001) in GO terms related to hematopoiesis and acetylation (Figure 5B; Figure S13H; Tables S14 and S15), which was further confirmed by RT-qPCR results (Figures S13I and S13K; Table S16).

In the absence of either *EPC1* or *EPC2* or both, K562 cells exhibited remarkable reduction (p < 0.001) in H3K9Ac (Figure 5C1; Figure S14A1), H3K27Ac (Figure 5C2; Figure S14A2), and H3K56Ac (Figure 5C3; Figure S14A3), with remarkable enrichment (p < 0.001) in GO terms related to acyl-CoA biosynthetic process (Figure 5A) and histone acetylation, histone H2A acetylation, etc. (Figure 5B), while H3K27Me3 showed increased level (Figure S14B). Meanwhile, the decrease of acetylated histones and hematopoiesis-related genes could be partially restored by treatment with VPA (Figures 5D and 5E; Figure S14C and S14D), which was in line with the results in zebrafish (Figures S7H and Figures 3D–3F). Furthermore, knockdown of *EPC1* or *EPC2* or both caused obvious downregulation (p < 0.001) of DLST protein in K562 cells versus the control (Figure 5F) and obvious downregulation in binding enrichment of H3K27Ac on promoter of gene *DLST* (Figure 5G), and the decreased *DLST* expression could also be rescued by VPA treatment (Figure 5H). Overall, our findings supported the conserved roles of EPC1 and EPC2 in hematopoietic development via regulation of histone H3 acetylation and *DLST*, and *DLST* expression was modulated by histone H3 acetylation, which might be specific in hematopoiesis.

### EPC1 and EPC2 co-operate with histone acetylation factors KATs and BRPF1 in modulating DLST

**Figure 6:**
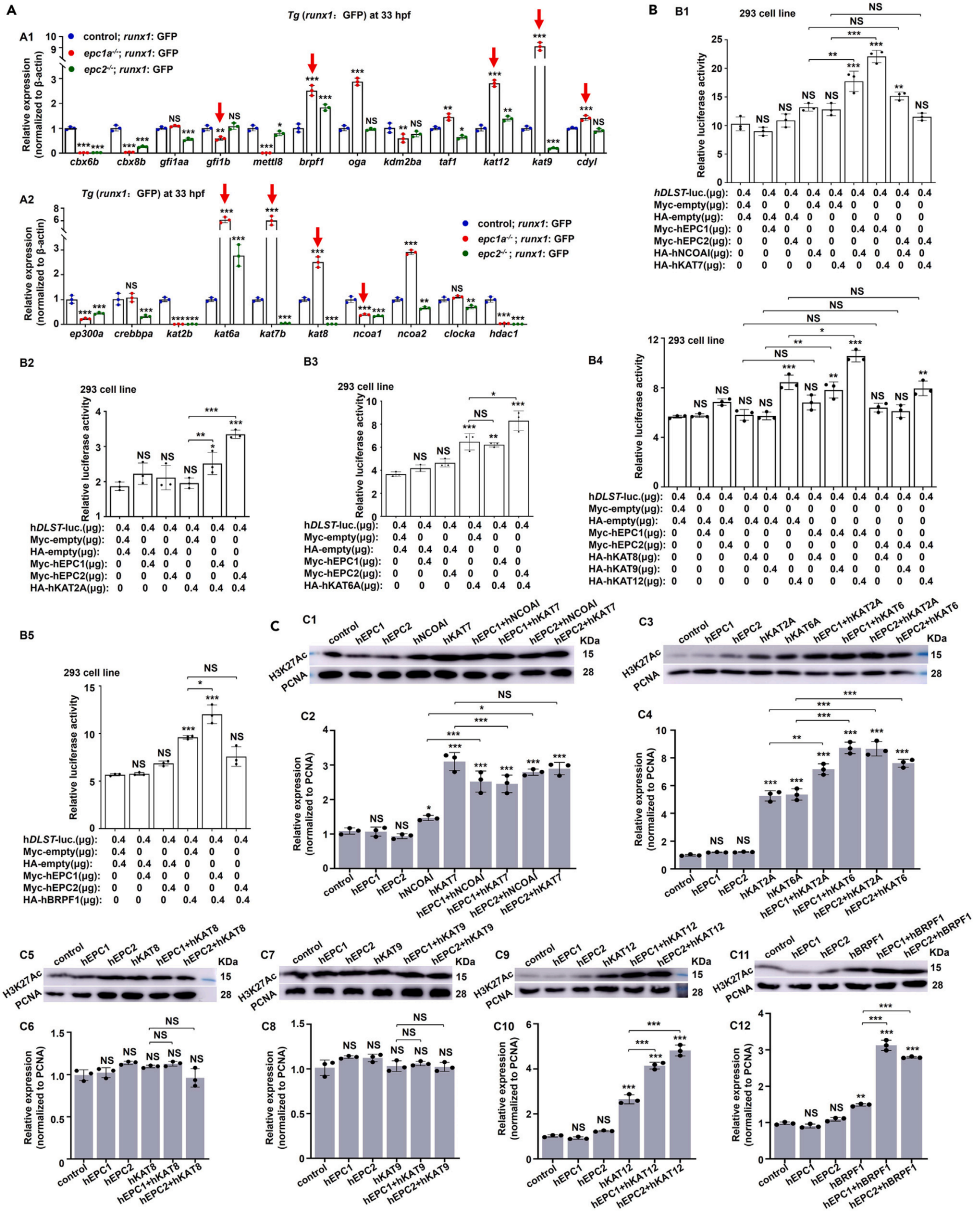
EPC1 and EPC2 cooperate with histone acetylation regulators (A) Expression of genes in histone acetylation machinery in *runx1*^*+*^ cells at 33 hpf, with red arrows indicating the genes verified by luciferase reporter assays in this study. (B) The transcriptional activities of the *DLST* promoter through co-transfection of EPC1 and EPC2 with hKAT7, hNCOA1 (B1), hKAT2A (B2), hKAT6A (B3), hKAT8, hKAT9 and hKAT12 (B4), and hBRPF1 (B5) in 293T cells, respectively. (C) Western blotting analysis of acetylated histone H3K27Ac in cells co-transfected of *EPC1* or *EPC2* with hNCOA1, hKAT7 (C1 and C2), hKAT2A, hKAT6A (C3 and C4), hKAT8 (C5 and C6), hKAT9 (C7 and C8), hKAT12 (C9 and C10), and hBRPF1 (C11 and C12), respectively, with PCNA as the internal controls. Data are presented as mean ± SD (n ≥ 3). t test, ∗p < 0.05, ∗∗p < 0.01, ∗∗∗p < 0.001, NS, not significant.

Previous studies have shown the involvement of EPC1 and EPC2 in HAT complexes,^26^^,^^27^^,^^41^ and the aforementioned results showed that VPA could rescue the expression of *DLST*, which acted as a downstream gene of *EPC1* and *EPC2* in *runx1*^*+*^ HSPCs and K562 cells, suggesting that the transcription of *DLST* might be modulated via histone acetylation. In this study, we found that acetylation-related genes, such as *ep300a*, *kat8*, *ncoa2*, and *gfi1a/b*, were highly expressed in *runx1*^+^ HSPCs (Figure S15B). Meanwhile, *epc1a-* or *epc2-*deficient HSPCs and embryos both exhibited marked changes in the expressions of a series of the histone acetylation genes, such as *kat2a*, *kat6a*, *kat7b*, *ncoa1*, *kat9*, and *brpf1* (p < 0.001) compared with their expression in the control (Figure 6A; Figure S15A). We speculated that the upregulated expressions of acetylation-related genes in EPC1/2 complexes in HSPCs might prefer to maintain the complex functions with either *EPC1* or *EPC2* functional deficiency, as genetic compensation response (GCR) reported recently.^42^^,^^43^ Thus, using *DLST* promoter activities and protein levels of H3K27Ac as indicators, we tested the interactions of EPC1/2 with the candidate histone acetylation-related regulators (Figure 6A, red arrowhead). The transcriptional activities of the *DLST* promoter were seen to be significantly activated (p < 0.01) through collaboration of histone acetylation writers KAT2A (GCN5), KAT6A (MYST3/MOZ), KAT12 (GTF3C4) or KAT13A (NCOAI), and histone acetylation reader BRPF1 (Figure 6B; Figure S15C), with either EPC1 or EPC2 (Table S17), coupled with increased levels of acetylated proteins in the co-transfected cells (Figure 6C; Figure S15D). Taken together, EPC1 and EPC2 may modulate histone acetylation by collaborating with histone acetylation regulators KATs and BRPF1, then to regulate *DLST* expression, and subsequently regulate HSPC development.

### EPC1 and EPC2 transcriptionally regulate *DLST* in cooperation with SRF or FOXR2

**Figure 7:**
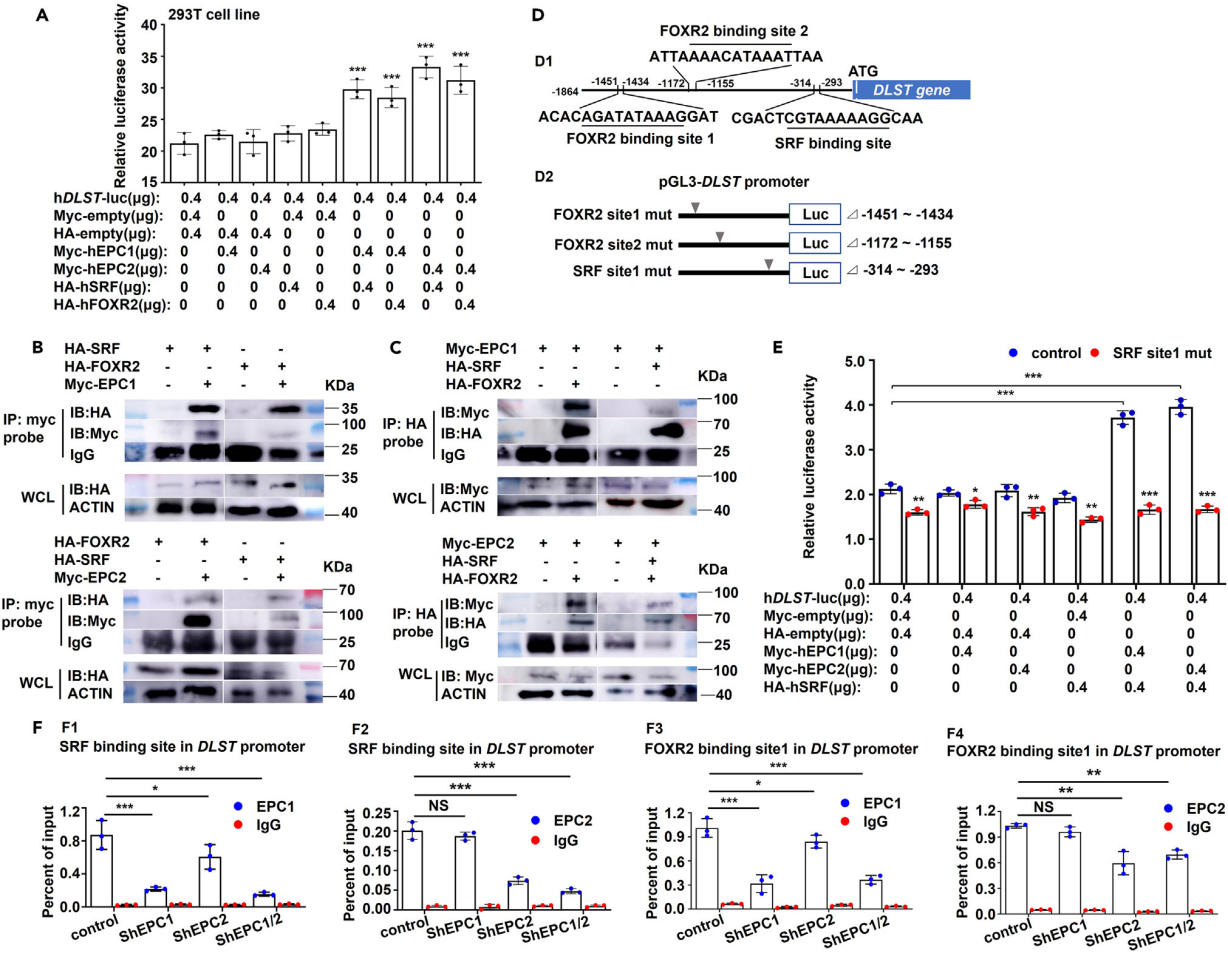
EPC1 and EPC2 transcriptionally regulate *DLST* expression by interacting with SRF and FOXR2 (A) The transcriptional activities of the *DLST* promoter through co-transfection of EPC1 and EPC2 with hSRF or hFOXR2 in 293T cells, respectively. (B and C) CoIP analysis of the interaction between hEPC1, hEPC2, hSRF, and hFOXR2. IP, immunoprecipitated; IB, immunoblot; WCL, whole cell lysate. (D) Schematic illustration of the predicted binding sites of SRF and FOXR2 in DLST promoter region (D1), and DLST promoter (+13–1864 bp) mutated with SRF and FOXR2 binding sites named control, FOXR2 site1 mut, FOXR2 site2 mut, and SRF site1 mut, respectively (D2), the mutated regions are marked with arrows. (E) Luciferase reporter assays of *DLST* promoter with mutated SRF binding site. (F) ChIP-qPCR analysis of EPC1 and EPC2 occupancies at predicted SRF binding site and FOXR2 binding site in *DLST* promoter in the control, ShEPC1, ShEPC2, and ShEPC1/2 cells. The occupancy was presented as the percentage of input. Each experiment was repeated three times, and a representative result is shown. Data are presented as mean ± SD (n ≥ 3). t test, ∗p < 0.05, ∗∗p < 0.01, ∗∗∗p < 0.001, NS, not significant.

EPC1 and EPC2 have been reported to be involved in transcriptional regulation as cofactors by interacting with transcription factors.^44^^,^^45^^,^^46^^,^^47^ The potential protein interaction between EPC and SRF or FOXR2 (forkhead box R2) was found by retrieving GeneCards database (https://www.genecards.org/). In this study, the transcriptional levels and protein levels of SRF and FOXR2 were significantly decreased in EPC1- or EPC2-deficient *runx1*^+^ cells (Figure S16A) and K562 cells (Figures S16B and S16C). The binding sites of SRF on *hDLST* (human *DLST*) and *zdlst* (zebrafish *dlst*) promoter region has been reported and referenced in many studies,^48^^,^^49^^,^^50^ and the FOXR2 binding sequence were predicted by consulting JASPAR database (http://jaspar.genereg.net/) (Table S18; Figure S17).^51^ To verify the predicted results, we performed dual-luciferase reporter experiments and observed that EPC1 and EPC2 significantly enhanced (p < 0.001) the *DLST* transcriptional activities by co-transfection with SRF or FOXR2 (Figure 7A). Co-immunoprecipitation assays further confirmed the interaction of EPC1 and EPC2 with SRF or FOXR2 (Figures 7B and 7C). Mutational analysis of the *DLST* promoter region showed that deletion of the predicted SRF binding site (CGTAAAAAGG) significantly decreased (p < 0.001) the transcriptional activity of *DLST* promoter when EPC1 or EPC2 was co-transfected with SRF, but deletion of the predicted FOXR2 binding sites in this study had no influence on the its transcriptional activity when co-transfecting EPC1 or EPC2 with FOXR2 (Figures 7D and 7E; Figure S18A). Furthermore, ChIP-qPCR assay indicated that knockdown of *EPC1* and *EPC2* led to significant decrease (p < 0.001) in EPC1/EPC2 and SRF/FOXR2 enrichments at the predicted SRF binding site and FOXR2 binding sites of *DLST* promoter in K562 cells (Figures 7F; Figures S18B and S18C). However, in zebrafish knock out embryos, their enrichments at the predicted SRF binding site were reduced (Figures S18C1 and S18C2), but remained unchanged at the predicted FOXR2 binding sites (Figures S18C3–S18C6).

In addition, we tried to delete the SRF or FOXR2 binding sites at zebrafish *dlst* promoter (Table S19) via the CRISPR-Cas9 system, and tested emergence of HSPCs in the F_0_ mutants. We effectively got a line of F_0_ mutants carrying an 8 bp insertion as shown in Figure S19A, which led to an increasing number of possible SRF motif (CArG^48^^,^^49^^,^^50^), and the F_0_ mutants exhibited increased *cmyb* expression at 33 hpf () and *runx1*^+^BrdU^+^ cells (Figure S19C) compared with the WT control. Meanwhile, ectopic expression of *srf* and *foxr2* mRNA could partially recover the decrease of *cmyb* at 33 hpf (Figure S20A) and *runx1*^+^BrdU^+^ cells at 72 hpf (Figures S20B–S20C) in *epc1a*^*−/−*^ and *epc2*^−/−^ mutants.

Collectively, these results suggested that EPC1 and EPC2 may recruit SRF or FOXR2 to transcriptionally activate *DLST* expression, and the predicted SRF/FOXR2 binding site might be the potential target of EPC1/EPC2 and SRF/FOXR2 complex on *DLST* promoter specifically in HSPCs.

## Discussion

### *EPC1* and *EPC2* are indispensable for HSPC emergence and proliferation

*EPC1* and *EPC*2 have been reported to play essential roles in leukemic hematopoiesis^20^ and involve in primitive erythropoiesis in zebrafish,^21^ but how the deficiency of *EPC1* and *EPC*2 induces hematopoiesis developmental defects is unknown. Here, we show that *EPC1* and *EPC2* are indispensable for HSPC emergence and proliferation via regulation of histone H3 acetylation and modulation of *DLST* expression. Apart from demonstrating EPC1 and EPC2 in histone H3 acetyltransferases complexes to regulating HSPC emergence and proliferation, this study unveils an important linkage of EPC1/2 with a TCA enzyme DLST then with HSPCs, and unveils the HSPC-specific role of DLST, downstream of EPC1/2 to link the epigenetic factors with mitochondrial metabolism, regulates HSPC emergence and proliferation.

A series of observations in this study support the essential roles of *EPC1* and *EPC2* in HSPC development. First, the expressions of the HSPC markers *runx1/cmyb* are markedly decreased in *epc1a*- or *epc2-*deficient embryos and *runx1*^+^ HSPCs. Second, depletion of either *epc1a* and *epc2* results in impaired proliferation, malformed spindles, and impaired cell cycle in *runx1*^+^ HSPCs. Third, knockdown of either *EPC1* or *EPC2* or both restrains cell proliferation in K562 cells. These observations are consistent with the reports that *EPC1* or *EPC2* are involved in cell cycle progression^26^^,^^52^ and cell proliferation^53^ in yeast and mammalian cells, and unveil the essential roles of EPC1 and EPC2 in HSPC proliferation. Knockdown of *EPC1* or *EPC2* induced cell apoptosis in K562 cells rather than in embryonic HSPCs, not only agreeing with the reports that knockdown of *EPC1* and/or *EPC2* induced apoptosis in AML cells,^20^ but also suggesting that roles of EPC1 and EPC2 in apoptosis are different between *in vivo* and *in vitro* cells. Collectively, our data shed light on the evolutionarily conserved roles of EPC1 and EPC2 in hematopoietic development.

Additionally, HSPC defects were more severe in *epc1a*^−/−^*epc2*^−/−^ embryos, and the ectopic expression of *epc2* mRNA partially restores the decrease of *runx1/cmyb* in *epc1a*^−/−^ embryos and vice versa, suggesting *epc1a* and *epc2* may play redundant roles in HSPC development. Some studies have confirmed that organisms can upregulate related family genes to response to mutations by genetic compensation mechanism.^42^^,^^43^ In this study, the transcriptional adaptations between the three genes (*epc1a*, *epc1b*, and *epc2*) in *runx1*^*+*^HSPCs and whole embryos are also observed.

### *EPC1* and *EPC2* regulate HSPC emergence and proliferation by modulating histone H3 acetylation and epigenetically regulating *DLST*

EPC1 and EPC2, the components of the EP400 and Tip60-EP400 complexes, have been reported to activate gene expression by stimulating H3.3 deposition into promoters and enhancers in U2OS human osteosarcoma cells.^54^ Here, we show that *EPC1* and *EPC2* could positively modulate the levels of histone acetylation in embryonic HSPCs and K562 cells, especially H3 histones, such as H3K9Ac, H3K27Ac, and H3K56Ac. Histone deacetylase inhibitors VPA could efficiently curb these inhibitory effects on the levels of acetylated histones and expressions of HSPC genes (*CMYB* and *RUNX1*) in either *EPC1*- or *EPC2*-deficient K562 cells and embryonic HSPCs, confirming that *EPC1* and *EPC2* contribute to H3 histone acetylation and the subsequent development of hematopoietic cells. *Epl1* (yeast homologous gene of *EPC1*) depletion has been shown to induce the global loss of acetylated histone H4 and H2A in yeast.^26^ In this study, enriched GO terms of histone H4 acetylation and decreased H4K5/K8/K12/K16Ac level were observed in *epc1a*^−/−^ or *epc2*^−/−^ mutant, and numerous genes regulated histone acetylation, such as *ep300/crbbpa*, *ncoa1*, *clocka*, and *kat2b*, which are capable of acetylating histone H2A/B or H4,^55^ had a remarkable decrease in *epc1a-* and *epc2-*deficient HSPCs. Moreover, *epc1a-* and *epc2*-deficient embryonic cells show increased H3K27Me3 protein and normal H3K4Me1, but with significant reduction in the levels of H3K9Ac, H3K27Ac, and H3K56Ac which also exhibit significantly reduced expression in *EPC1-* and *EPC2*-deficient K562 cells, further supporting that histone H3 acetylation and methylation are regulated in a seesaw manner in cells,^56^^,^^57^ and demonstrating the important roles of both EPC1 and EPC2 in histone H3 acetylation in HSPCs apart from their roles in H2 or H4 acetylation.

Here, we show that (1) functional deficiency of *EPC1* and *EPC2* induces the downregulation of *DLST* specifically in zebrafish embryonic HSPCs and K562 cells; (2) *dlst* morphants and mutants also show reduced HSPC emergence and proliferation; and (3) ectopic expression of *dlst* could effectively recover impaired HSPC proliferation in *epc1a*^*−/−*^ and *epc2*^*−/−*^ mutants. These suggest the specific role of *DLST* as a downstream target of *EPC1* and *EPC2* in regulating HSPC proliferation, which may widen the role of *DLST* besides its role in MYC-mediated leukemogenesis^32^ and neuroblastoma,^33^ and also widen the roles of *EPC1* and *EPC2* in regulating HSPC development via *DLST* related mitochondria metabolism. Meanwhile, we show that VPA could effectively recover the reduced *DLST* expression in embryonic HSPCs and mammalian K562 cells induced by the functional deficiency of either *EPC1* or *EPC2*, suggesting *EPC1* and *EPC2* may regulate *DLST* via histone acetylation machinery to cooperate with chromatin factor complexes.

Moreover, we demonstrate that writers in histone acetylation, such as GNAT family members, KAT2A and KAT9, MYST family members, KAT6A and KAT7, NCOA1 family member KAT13A, other HAT family member KAT12A and reader BRPF1, could significantly elevate the levels of acetylated H3 proteins and the transcriptional activities of *DLST* by co-transfection with *EPC1* or *EPC2*. These findings not only support that *EPC1* and *EPC2* could positively regulate *DLST* via histone acetylation machinery in HSPCs specifically, but also suggest that the aforementioned proteins might be the potential co-components in EPC1 or EPC2 histone acetylation machinery. Meanwhile, these results are supported by the previous studies about the functions of KAT2A,^58^^,^^59^ ELP3,^60^ KAT6A,^61^ KAT7,^62^ KAT8,^63^^,^^64^ NCOA1,^65^ and BRPF1^66^^,^^67^ in hematopoiesis. However, it is still unknown regarding the specific containing EPC1 and EPC2 histone acetylation complex in HSPCs, especially the specific writers of histone acetylation and HSPC factors for EPC1 and EPC2 histone acetylation machinery in HSPC proliferation.

Moreover, we show that deficiency of *epc1a* and *epc2* induces reduced *dlst* specifically in definitive HSPCs while increased *dlst* in the whole embryos and trunk muscle, with increased *meox1* in the whole *epc1a*^*−/−*^ embryos. *Meox1* is well-known in regulating somite-derived endothelial cells and the subsequent hematopoietic stem cell induction via restricting endotome which contributes to dorsal aorta colonization.^68^ We speculate that increased *meox1* might expand other somite cell types at the expense of definitive HSPC contributor endotome, resulting in the increased *dlst* in somites while reduced *dlst* in definitive HSPCs. Similarly, knockdown of EPC1 or EPC2 results in downregulation of DLST protein level and transcriptional level in K562 cells (as an *in vitro* model of hematopoietic development^23^^,^^39^), further confirming the function of DLST specifically in the development of hematopoietic cells, and EPC1/EPC2 positively regulate DLST expression specifically in hematopoiesis.

### *EPC1* and *EPC2* bind with SRF or FOXR2 to regulate *DLST* transcriptionally

EPC1 and EPC2 have been reported to interact with various complexes^69^^,^^70^ or bind with FOXO (forkhead box protein O) to trigger the differentiation of multipotent hematopoietic progenitors in *Drosophila*.^46^ EPC1 has been shown to regulate neointima formation after arterial balloon injury by interacting with Myocd (myocardin) and SRF.^71^ Here, we show that EPC1 and EPC2 could significantly enhance *DLST* transcriptional activity by co-transfection with SRF or FOXR2, and the direct interactions are observed between EPC1/EPC2 and SRF or FOXR2, suggesting the potential role of transcriptional complex of EPC1/EPC2 with either SRF or FOXR2 in *DLST* expression and the subsequent HSPC proliferation. It has been reported that as DNA-binding transcriptional factors, SRF is involved in the development of megakaryocytes,^72^ neutrophils,^73^ and HSPCs,^74^ and FOXR2 is associated with tumorigenesis.^75^^,^^76^ In this study, we identify the binding sites of SRF or FOXR2 at *DLST* promoter by dual-luciferase assays and ChIP-qPCR, and find that EPC1/EPC2 contact with SRF to effectively recognize the predict SRF binding sequence CGTAAAAAGG in *DLST* promoter to active its expression. Additionally, F_0_ mutants carrying an 8 bp insertion with an increasing number of possible SRF motif (CArG) on gene *dlst* promoter exhibit increased *cmyb* expression and *runx1*^+^BrdU^+^ cells at 33 hpf, further demonstrating that Epc1/2 might cooperate with SRF to regulate Dlst expression and the subsequent HSPC emergence. Meanwhile, EPC1/EPC2 contact with FOXR2 to recognize the predict FOXR2 binding sequence in *DLST* promoter effectively in HSPCs while not in the whole embryos and in 293T cells, suggesting the difference and complex of EPC1/EPC2 and FOXR2 interaction in regulating *DLST*.

Based on the aforementioned findings, we proposed a model for EPC1 and EPC2 regulation of HSPC proliferation through direct modulation of histone acetylation machinery by interacting with KAT2A/KAT6A/KAT12/KAT13A, and regulation of *DLST* by recruiting SRF/FOXR2 and modulating histone acetylation machinery, with *DLST* as a downstream target of *EPC1* and *EPC2* to facilitate HSPC emergence and proliferation. Numerous studies have proved that metabolic pathways, such as fatty acid oxidation,^77^^,^^78^ glycolysis,^79^^,^^80^ oxidative phosphorylation (OXPHOS),^81^^,^^82^^,^^83^ and glutamine metabolism,^34^^,^^84^^,^^85^ play key roles in HSPC commitment and erythroid differentiation.^86^ The mitochondrial citrate metabolism (TCA cycle), has also been reported to regulate hematopoietic development through OXPHOS^36^ and reactive oxygen species (ROS).^87^ DLST, as one of α-KGDH enzymatic complex components, its inactivation could lead to significant alterations in TCA cycle and ROS in triple-negative breast cancer,^88^ and suppressed NADH production and impaired OXPHOS in high-risk neuroblastoma.^33^ Hence, we speculate the downregulation of DLST resulted from loss of EPC1 and EPC2 may cause HSPC defect by disturbing OXPHOS and ROS generation controlled by TCA cycle. In addition, acetyl-CoA, as one of metabolite in TCA cycle, play important roles in cell proliferation,^89^ cell cycle progression,^90^ and cell differentiation^91^ via regulation of histone acetylation. In this study, GO terms related to acetyl-CoA were remarked enrichment in ShEPC1, ShEPC2, and ShEPC1/2 cells based on RNA-seq data, with the decreased expression of *DLST*. Therefore, in this study, the positive feedback loop between DLST and histone acetylation may be formed to regulate HSPC development, and DLST might function to be a pivotal linkage between EPC1/EPC2 with mitochondrion metabolism in HSPC development.

In this study, an 18 bp insertion in exon 8 in *dlst* gene induces almost no Dlst protein could be detected in *dlst*^−/−^ mutants, and we predict that non-frameshifting (NFS, an insertion or deletion of a multiple of three nucleotides) might occur in the mutants. NFS in coding regions has been reported to affect protein function, and cause a series of diseases and developmental defects in human,^89^^,^^92^^,^^93^^,^^94^^,^^95^ in mice,^94^ in rabbit,^96^ in horses,^97^ in quail,^98^ and so on. In this study, the NFS insertion of 18 bp fragment in the exon 8 of gene *dlst* in the mutants resulted in alteration of DLST protein structure predicted by SWISSMODEL,^99^ which may change its protein enzyme activity and stability, and then affect TCA cycle.

In this study, loss of *EPC1* and *EPC2* are unveiled to impair the development of hematopoietic cells, which are conserved from fish to mammalian. Meanwhile, EPC1 and EPC2 are unveiled to function in histone H3 acetylation apart from their roles in H2 or H4 acetylation in HSPCs via co-operating with histone acetylation writers KAT2A, KAT9, KAT6A, KAT7, KAT13A, KAT12A and reader BRPF1, but the HSPC-specific histone acetylation writer and reader in EPC1 and EPC2 acetyltransferase complexes still wait for discovery. Moreover, EPC1 and EPC2 are unveiled to positively regulate *DLST* expression specifically in HSPCs, and *DLST* links EPC1 and EPC2 with mitochondrial metabolism in HSPC proliferation.

### Limitations of the study

In this study, we show that EPC1 and EPC2 regulate hematopoietic development by histone H3 acetylation machinery and DLST. However, there still are some unanswered questions regarding the HSPC-specific histone acetylation writers or readers in EPC1 and EPC2 acetyltransferase complexes, and the different molecular mechanisms between EPC1 and EPC2 in regulating hematopoietic development, these questions need to be addressed in future studies.

## STAR★Methods

### Key resources table

**Table:** 

REAGENT or RESOURCE	SOURCE	IDENTIFIER
**Antibodies**		

Mouse polyclonal anti-EPC1	Biodragon	Cat# BD-PB3199;
Rabbit polyclonal anti-EPC2	ABclonal	Cat# A18447; RRID: AB_2862214
Rabbit polyclonal anti-Acetyl-Histone H3-K9	ABclonal	Cat# A7255; RRID: AB_2737400
Rabbit polyclonal anti-Acetyl-Histone H3-K27	ABclonal	Cat# A7253; RRID: AB_2767797
Rabbit polyclonal anti-Acetyl-Histone H3-K56	ABclonal	Cat# A7256; RRID: AB_2767800
Rabbit monoclonal anti-Methyl-Histone H3 (Lys4)/H3K4me1	Affinity Biosciences	Cat# DF6933; RRID: AB_2838892
Rabbit polyclonal anti-Tri-Methyl-Histone H3 (Lys27)/H3K27me3	Affinity Biosciences	Cat# DF6941; RRID: AB_2838900
Rabbit polyclonal anti-Acetyl-Histone H4K5/K8/K12/K16	ABclonal	Cat# A20764;
Rabbit polyclonal anti-DLST	ABclonal	Cat# A13297; RRID: AB_2760156
Rabbit polyclonal anti-SRF	Proteintech	Cat# 16821-1-AP; RRID: AB_2194384
Rabbit polyclonal anti-FOXR2	Proteintech	Cat# 14111-1-AP; RRID: AB_2878015
Rabbit monoclonal anti-Myc-Tag	ABclonal	Cat# AE070; RRID: AB_2863795
Mouse monoclonal anti-HA-Tag	Covance	Cat# MMS-101P; RRID: AB_2770404
Rabbit polyclonal anti-Histone H3	ABclonal	Cat# A2348; RRID: AB_2631273
Rabbit monoclonal anti-beta Actin	ABclonal	Cat# AC026; RRID: AB_2768234
Rabbit polyclonal anti-PCNA	ABclonal	Cat# A0264; RRID: AB_2757077
Rabbit polyclonal anti-GFP-Tag	ABclonal	Cat# AE011; RRID: AB_2771922
Mouse monoclonal anti-alpha Tubulin	GeneTex	Cat#GT114, RRID: AB_2716636
Mouse monoclonal anti-BrdU	ABclonal	Cat# A1482; RRID: AB_2756438
HRP-labeled goat anti-rabbit IgG	Beyotime	Cat# A0208; RRID: AB_2892644
HRP-labeled goat anti-rat IgG	Beyotime	Cat# A0192; RRID: AB_2939016
555-conjugated Goat Anti-Mouse IgG (H + L) secondary antibody	ABclonal	Cat# AS057; RRID: AB_2768321
Goat Anti-Rabbit IgG FITC (H + L) secondary antibody	Biosharp	Cat# BL033A; RRID: AB_2769478

**Bacterial and virus strains**		

DH5a competent *E. coli*	This paper	N/A

**Chemicals, peptides, and recombinant proteins**		

Puromycin	Beyotime	Cat# ST551
Valproic acid	Aladdin	Cat# V298968
TRIzol reagent	TaKaRa	Cat# 9018
RIPA lysis buffer	Biosharp	Cat# BL509A
Proteinase inhibitor	Invitrogen	Cat# 89900
Collagenase	Life-iLab Biotech	Cat# AC15L141
DAPI	Beyotime	Cat# C1005
BrdU	Beyotime	Cat# ST105
RNase A	Roche	Cat# 10109142001
Lipofectamine™ 2000	Invitrogen	Cat# 11668
BSA	Sigma	Cat# V900933

**Critical commercial assays**		

M-MLV Reverse-Transcript Kit	Applied Biological Materials	Cat# G492
Cell DirectTM One-Step qRT-PCR Kit	Invitrogen	Cat# 11753-100
Ambion MAXIscript T7 Kit	Invitrogen	Cat# AM1344
Transcript T7 High Yield Transcription kit	Invitrogen	Cat# K0441
Annexin V-PE Apoptosis Detection Kit	Beyotime	Cat# C1065S
Calcein AM Cell Viability Assay Kit	Beyotime	Cat# C2013FT
SuperScript™ IV First-Strand Synthesis System	Invitrogen	Cat# 18091050
2× MultiF Seamless Assembly Mix kit	ABclonal	Cat# RK21020
Dual-Luciferase Reporter Assay System	Promega	Cat# E1910

**Deposited data**		

RNA sequencing (zebrafish)	This paper	https://ngdc.cncb.ac.cn/search/?dbId=gsa&q=CRA010982
RNA sequencing (human cell lines)	This paper	https://bigd.big.ac.cn/gsa-human/browse/HRA004692

**Experimental models: Cell lines**		

Human leukemia cell line K562	CCTCC	Cat# GDC0037
Human embryonic kidney (HEK) 293 cell	ATCC	Cat# CRL-3216
ShEPC1, ShEPC2 and ShEPC1/2 K562 cells	This paper	N/A

**Experimental models: Organisms/strains**		

Zebrafish AB line (WT)	This paper	N/A
Zebrafish：*Tg* (*rag2*: dsRed)	Zhang et al.^100^	N/A
Zebrafish：*Tg* (*flk1*: mCherry/*runx1*: GFP)	Zhang et al.^101^	N/A
Zebrafish：*Tg* (*runx1*: GFP)	Zhang et al.^101^	N/A
Zebrafish：*epc1a*^−/−^	This paper	N/A
Zebrafish：*epc2*^−/−^	This paper	N/A
Zebrafish：*epc1a*^−/−^*epc2*^−/−^	This paper	N/A
Zebrafish：*dlst*^−/−^	This paper	N/A

**Oligonucleotides**		

gRNA targeting sequences, see Table S2	This paper	N/A
Primers sequences used for screening homozygous mutants Table S3	This paper	N/A
Sequences of human for ShRNA, see Table S4	This paper	N/A
Sequences of zebrafish for Morpholinos, see Table S5	Gene Tools	N/A
Sequences of primers for mRNA synthesis, see Table S6	This paper	N/A
Zebrafish primer sequences used for qRT-PCR, see Table S7	This paper	N/A
Human primer sequences used for qRT-PCR, see Table S8	This paper	N/A
Sequences of primer used for amplifying probes for WISH, see Table S9	This paper	N/A
Sequences of primer used for luciferase reporter assay and CoIP, see Table S10	This paper	N/A
Sequences of primer used for ChIP-qPCR, see Table S11	This paper	N/A

**Software and algorithms**		

ImageJ	ImageJ	https://imagej.net/software/imagej/
GraphPad Prism 8.00	Graphpad	https://www.graphpad.com/
Statistic Package for Social Science (SPSS) 19.0	Spss	https://www.concordia.ca/it/services/spss.html
ClustalX 2.0	ClustalX	http://www.clustal.org
GeneDoc 2.7	GeneDoc	https://genedoc.software.informer.com/2.7

### Resource availability

#### Lead contact

Further information and requests for resources should be directed to and will be fulfilled by the lead contact, Jing-Xia Liu (ichliu@mail.hzau.edu.cn).

#### Materials availability

All materials generated in this study are available from the lead contact without restriction.

#### Data and code availability

• RNA-seq data have been deposited at Genome Sequence Archive (GSA) and are publicly available as of the date of publication. Accession numbers are listed in the key resources table.
• This paper does not report original code.
• Any additional information required to reanalyze the data reported in this paper is available from the lead contact upon request.

### Experimental model and study participant details

#### Zebrafish husbandry and lines

All zebrafish in this study were bred and maintained in the Zebrafish Aquaculture Core Facility at College of Fisheries (Huazhong Agricultural University) with circulating filtration system on a 14-h light/10-h dark cycle at 28°C, and fed with fairy shrimp twice one day.^102^ The following fish lines were used in this study: wild type (AB line), *Tg* (*rag2*: dsRed),^100^ *Tg* (*flk1*: mCherry/*runx1*: GFP),^101^ and *Tg* (*runx1*: GFP),^101^ as well as mutants of *epc1a*^−/−^, *epc2*^−/−^, *epc1a*^−/−^*epc2*^−/−^, *dlst*^−/−^, *Tg* (*epc1a*^−/−^; *flk1*: mCherry/*runx1*: GFP), *Tg* (*epc2*^−/−^; *flk1*: mCherry/*runx1*: GFP), *Tg* (*epc1a*^−/−^; *runx1*: GFP) and *Tg* (*epc2*^−/−^; *runx1*: GFP). The age of the embryos or larvae was expressed in hours postfertilization (hpf) or days post fertilization (dpf). The embryos or larvae of age from 8 to 96 hpf were used in this study, and they could not be classified as males or females at the early stages, since the sex was regulated by many internal and external factors, and was not definitively determined until reaching near adulthood.^103^ All animals and experiments were conducted in accordance with the “Guidelines for Experimental Animals” approved by the Institutional Animal Care and Use Ethics Committee of Huazhong Agricultural University (HZAUFI-2016-007).

#### Cell lines

HEK293T cells (human embryonic kidney cell line) were purchased from American Type Culture Collection (Manassas, USA), and were maintained in Dulbecco’s modified Eagle’s medium (DMEM, Cat# C11875500BT, Gibco, USA) supplemented with 10% heat-inactivated fetal bovine serum (FBS, Cat# SE200-ES, HyClone, USA) in a humidified atmosphere with 5% CO_2_ in a 37°C incubator. Human leukemia cell line K562^104^ was obtained from cell bank of China Center for Type Culture Collection (CCTCC, Wuhan, China), and were cultured in RPMI 1640 medium (Cat# C11995500BT, Gibco, USA) supplemented with 10% heat-inactivated FBS at 37°C and 5% CO_2_.

### Method details

The full names and their abbreviations for the genes mentioned in this study are listed in Table S1.

#### Generation of zebrafish mutants using CRISPR/Cas9

*Epc1a*, *epc2*, and *dlst* in zebrafish (*Danio rerio*) were knocked out using CRISPR/Cas9 technolog.^22^^,^^23^ The guide RNAs (gRNAs) were designed using the CRISPR design tool (http://crispr.mit.edu) and synthesized by *in vitro* transcription using a Thermo Transcript Aid T7 High Yield Transcription Kit (Cat# K0441, Invitrogen, USA) with the primer sequences listed in Table S2. Cas9 mRNAs were obtained by the Ambion MAXIscript T7 Kit (Cat# AM1344, Invitrogen, USA). Mutants were generated by co-injecting the gRNAs (80 ng/μL) and Cas9 mRNA (500 ng/μL) into wild-type embryos at the one-cell stage, and homozygous mutants were screened by amplifying the genomic region flanking the gRNA target sites of each gene using the specific primers listed in Table S3. Finally, the following homozygous mutants were acquired: *epc1a*^*−/−*^ (with an 11 bp deletion in exon 1), *epc2*^*−/−*^ (with a 4 bp deletion in exon 1), and *dlst*^*−/−*^ (with an 18 bp insertion in exon 8). *epc1a*^*−/−*^*epc2*^*−/−*^ double mutant line was obtained by mating adult *epc1a*^*−/−*^ mutants with *epc2*^*−/−*^ adult mutants and screening the offspring of incrossed *epc1a*^*+/−*^ *epc2*^*+/−*^ heterozygous mutants.

#### RNA interference

Four ShRNAs specific to human *EPC1* and three ShRNAs specific to human *EPC2* were synthesized and cloned into the pLKO.1-puro vector using the primer sequences shown in Table S4, and were used for screen effective ShRNAs in knocking down expression of *EPC1* or *EPC2* in K562 cells in this study. Lentiviruses for different ShEPC1, ShEPC2, or ShGFP controls were generated by co-transfecting the packaging plasmid pCMV-dR8.91, envelope plasmid VSV-G/pMD2G and hairpin-pLKO.1 into 293T cells. K562 cells were infected with the lentivirus and then screened by puromycin (2–5 μg/mL) for at least 48 h. The efficiency of *EPC1* or *EPC2* knockdown was verified by qRT-PCR, with the primers for qRT-PCR listed in Table S8 and the ShGFP cells as the controls.^40^ Based on the data of knockdown efficiency of different ShEPC1 or ShEPC2, shEPC1-2 (abbreviated as shEPC1), shEPC2-3 (abbreviated as shEPC2) and shEPC1/2 (knockdown of both *EPC1* and *EPC2* with shEPC1-2 and shEPC2-3) were screened and further maintained as stable knock-down cell lines and were used for the next tests in this study.

#### Drug treatment

Zebrafish embryos from the control, *epc1a*^−/−^ and *epc2*^−/−^ mutants were treated with 0.2 mM valproic acid (VPA) (Cat# V298968, aladdin, China) beginning at 11 hpf. Next, the treated embryos were collected at 33 hpf or 72 hpf. Meanwhile, the cultured cells from control, ShEPC1, ShEPC2 and ShEPC1/2 cells were incubated with the similar deacetylase inhibitors VPA at 37°C for 16–18 h, followed by collecting the cell samples for the following experiments. In this study, the zebrafish embryos or larvae were collected at the indicated stages for experiments as shown in Schema 1.

#### Morpholino (MO) and mRNA injection

The morpholinos of *epc1a*, *epc2* and *dlst* were purchased from Gene Tools LLC (Philomath, Oregon, USA) and dissolved in ddH_2_O at 3 mM concentration (stock solution). The sequences of MOs are listed in Table S5. The *dlst* MO sequence has been reported previously.^21^ The full-length *epc1a*, *epc2* and *dlst* were amplified with the specific primers shown in Table S6, and synthesized using the Ambion MAXIscript T7 Kit (Cat# AM1344, Invitrogen, USA) as instructed by the manufacturer. In all experiments, the MOs and mRNAs were injected into one-cell stage embryos, with the MO doses of *epc1a*, *epc2* and *dlst* at 0.6 mM, 0.9 mM and 0.6 mM, and the mRNA concentrations of *epc1a*, *epc2*, *dlst*, *srf* and *foxr2* at 150 ng/μL, 150 ng/μL, 200 ng/μL, 200 ng/μL and 200 ng/μL, respectively.

#### RNA-sequencing (RNA-Seq) and analysis

In this study, fifty embryos of control and *epc1a*^−/−^ mutants at 28 hpf, and K562 cells named control, ShEPC1 (*EPC1* knockdown), ShEPC2 (*EPC2* knockdown), and ShEPC1/2 (both *EPC1* and *EPC2* knockdown), were collected separately and used for mRNA extraction. The cDNA library construction and the next RNA sequencing (RNA-Seq) was performed and run on a BGISEQ-500 and Illumina NovaSeq 5000 platforms, respectively. Sequenced reads were 150 bp long with paired-ends. After filtering the raw reads by SOAPnuke soft (v1.5.2),^105^ the sequencing data were mapped to zebrafish reference genome (GRCz11) using the HISAT (v2.0.4) and Bowtie2 (v2.2.5) tool.^25^^,^^106^^,^^107^ mRNA abundance was expressed as the number of FPKM (fragments per kilobase of transcript per million). DEGs were defined using the DEGSeq method following the criteria: |fold-change| ≥ 2 and adjusted p value<= 0.001.^108^ Enriched Kyoto Encyclopedia of Genes and Genomes pathway analysis was conducted for each sample using KOBAS v.2.0 based on the lists of DEGs. GO analysis was conducted using the lists of DEGs by GOseq Release 2.12. Hierarchical clustering was performed by TIGR Multi Experiment Viewer to generate different heatmaps. The sequencing data were deposited in GSA database (https://ngdc.cncb.ac.cn/gsa/),^109^^,^^110^ and accession numbers are listed in the key resources table.

#### Quantitative real-time PCR

In this study, total RNAs were extracted from the control, *epc1a*^−/−^ and *epc2*^−/−^ embryos, and *EPC1/2*-knockdown K562 cells by TRIzol reagent (Cat# 9018, TaKaRa, Japan), and reverse-transcribed to complementary DNA (cDNA) using an M-MLV Reverse-Transcript Kit (Cat# G492, Applied Biological Materials Inc., Canada). Zebrafish genes (*epc1a*, *epc1b* and *epc2*) and human genes (*EPC1*, *EPC2*, *RUNX1*, *CMYB*, *FLI1*, *FLK1*, *GATA1*, *GATA2*, *CD41*, *CD45*, *SRF* and *FOXR2*) were selected for qRT-PCR, and the primer sequences are available in Table S7 and Table S8, respectively. Each sample was run in triplicate and repeated at least three times. Differences were calculated by the ΔΔCt comparative quantization method using *β-actin* or *GAPDH* as an internal control.

#### One step cell-direct qRT-PCR

One Step Cell-Direct qRT-PCR followed the protocol of the manufacturer. In this study, *Tg* (*runx1*: GFP) (as control group), *Tg* (*epc1a*^−/−^; *runx1*: GFP), and *Tg* (*epc2*^−/−^; *runx1*: GFP) embryos were collected and homogenized in PBS supplemented with 5% FBS, and then centrifuged at 1000 × g for 10 min at 4°C. Next, the precipitate was resuspended in PBS and subsequently filtered through a 0.2 mm filter to make single-cell suspension. The GFP-positive cells (5000–10000 sorted cells/sample) were sorted into the lysis solution provided by the CellsDirect One-Step qRT-PCR Kit (Cat# 11753-100, Invitrogen, USA) using fluorescence-activated cell sorting based flow cytometry (FACS) (BD FacsAria SORP, 650110M3, BioDot, USA). The cultured cells were collected into the lysis solution from control, ShEPC1, ShEPC2 and ShEPC1/2 K562 cells at a cell density of less than 10^4^ cells/μL. The lysates were used as template for one-step qRT-PCR. Primer sequences for the tested genes in this study are shown in Table S7 and Table S8, including zebrafish genes *epc1a*, *epc1b*, *epc2*, *cmyb*, *runx1*, *myod*, *dlst*, *kat2b*, *kat8*, *kat7b*, *kat6a*, *ncoa1*, *ncoa2*, *clocka*, *crebbpa*, *ep300a*, *elp3*, *gtf3c4*, *taf1*, *mettl8*, *oga*, *cdyl*, *brpf1*, *kdm2ba*, *cbx6b*, *cbx8b*, *hdac1*, *gfi1b*, *gfi1aa*, *cdc25b*, *atm*, *ccnb1*, *ccna1*, *ccna2*, *ccnd1*, *ccng2*, *cenpf*, *srf* and *foxr2*, and human genes *PSME4*, *LDB1*, *ADAM8*, *CSF1*, *NOTCH1*, *JUN*, *ZMYND8*, *BRD8*, *PHIP*, *BRCA2*, *MLLT3*, *EID1*, *JADE1*, *MECP2*, and *LIF.*

#### Whole-mount in situ hybridization

In this study, the probes used include the antisense RNA probes (*c-myb*, *runx1*, *rag1*,^111^ *flk1*, *fli1*; ^112^ *pax2a*, *gata5*, *six6b*; ^40^ and *myoD*^113^ and the other hematopoiesis-related probes (*haus3*, *meox1*, *adnpb*, *lsm8*, *hspa9*, *ptena*, *ncor1*, *flvcr1*, *idh1*, *wasla*, *atpif1b*, *poln*, *sfxn4*, *kat8*, *klf3*, *dlst*, *mbd3b*, *nap1l4a*, *epc1a* and *epc2*)*,* which were synthesized using the specific primers listed in Table S9. WISH embryos in each group were observed and photographed using a stereoscopic microscope (Leica. M205FA, Germany). Data quantification and visualization were carried out using ImageJ software (NIH, Bethesda, Maryland)^114^ and GraphPad Prism 8.0 (GraphPad Software, USA), respectively. A minimum of 10 embryos per group were used for WISH analysis, and three independent experiments were performed. A representative image in each group is shown.

#### Western blot

In this study, the protein samples were extracted from zebrafish embryos of the control, *epc1a*^−/−^, *epc2*^−/−^ and *dlst*^−/−^ mutants, and K562 cells (control, ShEPC1, ShEPC1 and ShEPC1/2), respectively, using RIPA lysis buffer (Cat# BL509A, Biosharp, China) with proteinase inhibitor (Cat#89900, Thermo Fisher Scientific, USA). After adding SDS-PAGE loading buffer, the protein samples were denatured for 15 min in boiling water. Next, the samples were separated by 8% or 12% separation gels, and then transferred to PVDF (polyvinylidene fluoride) membrane (Bio-Rad Laboratories, Hercules, CA, USA). The membranes were blocked with 5% skim milk for 1 h, then followed by incubation with primary antibodies at 4°C overnight. The primary antibodies in this study were listed in key resources table. Next, the membranes were incubated with the secondary antibodies (HRP-labeled goat anti-rabbit IgG (Cat# A0208, Beyotime) or HRP-labeled goat anti-rat IgG (Cat# A0192, Beyotime)) for 1–2 h at room temperature (RT), and the blots were photographed using the Amersham Imager 600 analyzer system (GE Healthcare Life Sciences, USA). ImageJ software (NIH, Bethesda, Maryland) was used for quantifying the protein levels based on the band density obtained in the WB analysis.

#### Immunofluorescence

In this study, immunofluorescence of whole-mount zebrafish embryos was performed as follow. Firstly, the embryos or larvae from *Tg* (*runx1*: GFP) (control), *Tg* (*epc1a*^−/−^; *runx1*: GFP) and *Tg* (*epc2*^−/−^; *runx1*: GFP) at 33 hpf or 72 hpf were collected and fixed in 4% paraformaldehyde overnight, followed by permeabilizing the fixed samples with 1 mg/mL collagenase (Cat# AC15L141, Life-iLab Biotech, China) in PBST (PBS with 1% Triton X-100) for 45 or 75 min, and then blocking in 3% BSA for 1 h. The primary antibodies used for immunofluorescence included anti-GFP (Cat# AE011, ABclonal), anti-H3K9Ac, anti-H3K27Ac, anti-H3K56Ac, anti-α-Tubulin (Cat# GT114, GeneTex), and anti-Dlst antibodies, respectively. Alexa Fluor 555-conjugated anti-mouse (Cat# AS057, ABclonal) and FITC-conjugated anti-rabbit antibodies (Cat# BL033A, Biosharp, China) were used as secondary antibodies to visualize the fluorescence signals. DAPI (40, 6-diamidino-2-phenylendole) was used to label nuclei. Images were captured using a Leica TCS SP8 confocal laser microscope (Wetzlar, Germany).

Immunofluorescence for K562 cells was performed as follow. Firstly, the K562 suspension cells were transferred to slides, followed by fixation in 4% PFA for 10 min, permeabilization with 0.5% PBST (PBS with 0.5% Triton X-100) for 20 min and blocking in 5% BSA for 1 h. Finally, the cells were stained with the respective antibodies, and DAPI was used to label nuclei. Images were acquired by super resolution microscopy (STORM-A1R) in the State Key Laboratory of Agricultural Microbiology of Huazhong Agricultural University (Wuhan, China), and analyzed using NIS-Elements Viewer 4.50 (Nikon, Japan).

#### BrdU (5-bromodeoxyuridine) assays

The 5-bromo-20-deoxy-uridine (BrdU) labeling assay was performed by microinjecting BrdU (10 mM) (Cat# ST1056, Beyotime, China) into the yolk sac of embryos of *Tg* (*runx1*: GFP) (control), *Tg* (*epc1a*^−/−^; *runx1*: GFP) and *Tg* (*epc2*^−/−^; *runx1*: GFP), separately, followed by incubation for 2–3 h at 28°C, fixation in 4% PFA overnight, and permeabilization with 1 mg/mL collagenase. After washing with PBST, the embryos were incubated with 2 N HCl for 1 h at RT and neutralized with sodium borate (1 M, pH 8.5) for 20 min. After 3 washes with 1× PBST (with 1% Triton X-100), the embryos were blocked with 4% BSA for 1 h, followed by adding the anti-BrdU antibody (Cat# A1482, Beyotime, China) (diluted in 4% BSA) and Rabbit anti GFP-Tag pAb (1: 200) antibodies to the embryos according to the manufacturer’s protocol, then, incubation at 4°C overnight. After washing with 1× PBST, the embryos were incubated with Alexa Fluor 555 goat anti-mouse IgG (H + L) antibody and Goat Anti-Rabbit IgG FITC (H + L) (1:200) secondary antibodies. Images were captured using a Leica TCS SP8 confocal laser microscope (Wetzlar, Germany).

#### Confocal microscopy

For *in vivo* observation, the embryos from *Tg* (*flk1*: mCherry/*runx1*: GFP) at 33 and 72 hpf were anesthetized using 0.168 mg/mL tricaine (Sigma–Aldrich, USA), followed by observation and photographing under a confocal microscope (Leica M205FA, Germany). The number of *flk1*^*+*^*runx*^*+*^ cells was counted based on overlapping particles of red and green fluorescence in cells. Immunofluorescent signals of whole embryos at 33 and 72 hpf and K562 cells were scanned using a super resolution microscope (STORM-A1R) in the State Key Laboratory of Agricultural Microbiology of Huazhong Agricultural University (Wuhan, China). The images were processed and quantified using NIS-Elements Viewer 4.50 (Nikon, Japan).

#### Cell cycle, cell proliferation, and apoptosis analysis

For cell cycle analysis, the GFP-positive cells from *Tg* (*runx1*: GFP) (control), *Tg* (*epc1a*^−/−^; *runx1*: GFP), *Tg* (*epc2*^−/−^; *runx1*: GFP) embryos were sorted by FACS (BD FacsAria SORP 650110M3 BioDot, USA) and fixed in precooled 75% absolute ethanol for 2 h. Then, RNase A (10 mg/mL) was added to the cells and incubated for 30 min at 28°C. The nuclei were labeled with propidium iodide (PI, Invitrogen, USA). Flow cytometry was performed to assess the cellular DNA content using a CytoFLEX Flow Cytometer (Beckman Coulter, USA), and followed by analyzing the percentage of cells in the G1 phase, S phase, and G2/M phase.

To analyze the apoptosis of zebrafish HSPCs and K562 cells, embryos from *Tg* (*runx1*: GFP) (control), *Tg* (*epc1a*^−/−^; *runx1*: GFP) and *Tg* (*epc2*^−/−^; *runx1*: GFP) (100 embryos/sample) at 33 hpf, and *EPC1*/*EPC2* knockdown cells with their control, were harvested and dissociated separately into single cells in phosphate-buffered saline (PBS). Next, the cells were co-stained separately with Annexin V-PE and DAPI as instructed for the Annexin V-PE Apoptosis Detection Kit (Cat# C1065S, Beyotime, China), followed by analyzing Annexin V-PE apoptosis in GFP-positive cells using FACS (CytoFLEX S, Beckman Coulter, USA).

Cell proliferation in K562 cells was measured using a Calcein AM Cell Viability Assay Kit (Cat#C2013FT, Beyotime, China) following the protocol of the manufacturer. and then run on a flow cytometer (CytoFLEX S, Beckman Coulter, USA), where the cells were assessed for characteristics of light scatter properties (forward and side scatter) to determine the percentage of proliferating cells.

#### Bioinformatics analysis

Genecards (https://www.genecards.org/)^115^ was used to predict the proteins interacted with EPC1 and EPC2 based on STRING (Search Tool for the Retrieval of Interaction Gene/Proteins) and GPS-Prot interaction network analysis. The JASPAR database (http://jaspar.genereg.net/)^51^ was consulted to predict the potential transcription factors and their binding sites in *DLST* promoter region. ClustalX 2.0^116^ and GeneDoc^117^ were used for multiple sequence alignment. The 3D structures of WT DLST protein and mutant DLST protein were predicted using SWISSMODEL (https://swissmodel.expasy.org/).^99^

#### Plasmid construction

Total RNA and genomic DNA were extracted from 50 to 60 wild-type embryos or 293T cells followed by quantification using Nanodrop spectrophotometry (Thermo Fisher) and synthesizing cDNA through the SuperScript IV First-Strand Synthesis System (Cat#18091050, Invitrogen, USA). Full-length *EPC1*, *EPC2*, *SRF*, *FOXR2*, *KAT2A/GCN5*, *KAT6A/MYST3/MOZ*, *KAT7/MYST2/HBO1*, *KAT8/MYST1*, *KAT9/ELP3*, *KAT12/GTF3C4*, *KAT13A/NCOA1*, *BRPF1*, *CDYL* and *GFI1B* were amplified from cDNA pools using the primer sets listed in Table S10, and subcloned into the vectors of pCMV-Myc (Clontech) or pCGN-HAM (provided by William Tansey, Cold Spring Harbor Laboratory, Cold Spring Harbor, NY, USA). The *DLST* promoter were amplified from the genomic DNA using the primers shown in Table S10, followed by ligation into the pGL3 vector (Promega) using T4 DNA Ligase (Cat#C301-01, Vazyme, China). Mutations of *DLST* promoter were made by ligating two fragments without the predicted SRF or FOXR2 binding sites into pGL3 vector using 2× MultiF Seamless Assembly Mix kit (Cat# RK21020, ABclonal, China), with the primer sequences listed in Table S10. All constructs were verified by sequencing.

#### Luciferase reporter assay

In this study, the expression vectors (Myc-EPC1, Myc-EPC2, HA-SRF, HA-FOXR2, HA-KAT2A/GCN5, HA-KAT6A, HA-KAT7, HA-KAT8/MYST1, HA-KAT9/ELP3, HA-KAT12/GTF3C4, HA-KAT13A/NCOA1, HA-BRPF1, HA-CDYL and HA-GFI1B) and a reporter vector of *DLST* promoter were used for dual-luciferase reporter assay. 293T cells were cultured in DMEM supplemented with 10% FBS. After growing to a density of 70–75% in 24-well plates, Lipofectamine 2000 (Cat#11668, Invitrogen, USA) was used to transfect the cells with the indicated amounts of vectors, with Renilla as an internal control. At 48 h post-transfection, the luciferase activity was measured using the Dual-Luciferase Reporter Assay System (Cat# E1910, Promega, USA) following the protocol of the manufacturer. Each experiment was repeated three times. The data were normalized by Renilla luciferase activity and analyzed using GraphPad Prism 8.0.

#### Immunoprecipitation assays

For transient transfection, 293T cells were co-transfected with HA-FOXR2/SRF and Myc-EPC1/EPC2 expression vectors. At 36 to 48 h post transfection, cells were collected for immunoprecipitation assays. The primary antibodies anti-Myc-Tag, anti-HA-Tag and anti-β-Actin, and secondary antibodies HRP-labeled goat anti-rabbit IgG (H + L) (Cat# A0208, Beyotime) and HRP-labeled goat anti-rat IgG (H + L) (Cat# A0192, Beyotime) were used. Anti-HA-Tag antibody-conjugated agarose beads were purchased from Sigma (Cat# A2095). After addition of cell extracts (transfected with Myc tagged EPC1/EPC2 and HA tagged FOXR2/SRF) and incubation for 4 h at 4°C, the bound beads were washed with PBS and analyzed by WB analysis. The Amersham Imager 600 analyzer was used for photographing the blots. ImageJ software was used for quantifying the protein levels based on the band density obtained in the WB analysis.

#### Chromatin immunoprecipitation (ChIP)-qPCR

In this study, ChIP-qPCR experiments were performed using the embryos from the control, *epc1a*^−/−^ and *epc2*^−/−^ mutants at 33 hpf or cultured cells from control, ShEPC1, ShEPC2 and ShEPC1/2 K562 cells. Firstly, the cells were fixed in 1% formaldehyde to crosslink the protein and DNA, and then the crosslinking reaction was terminated with 0.125 M glycine. After two washes with PBS (with 1×protein inhibitor), the cells were centrifuged at 4°C, and the supernatant was discarded. The deposited cells were lysed successively with lysis buffer 1 (50 mM HEPES-KOH pH 7.5, 140 mM NaCl, 1 mM EDTA, 10% glycerol, 0.5% NP-40, 0.25% Triton X-100), lysis buffer 2 (10 mM Tris-HCl pH 8.0, 200 mM NaCl, 1 mM EDTA, 0.5 mM EGTA). and lysis buffer 3 (10 mM Tris-HCl pH 8, 100 mM NaCl, 1 mM EDTA, 0.5 mM EGTA, 0.1% Na-Deoxycholate, 0.5% N-lauroylsarcosine), followed by ultrasonic fragmentation to obtain ∼200-500bp chromatin DNA fragments. After centrifugation for 10 min at 18,000 g, and a total of 50 μL of the supernatant (totaling 1 mL) was taken as the input group. Next, protein A + G agaroses (Beyotime, Cat#P2055) were co-incubated with primary antibodies (anti-H3K27Ac, anti-EPC1, anti-EPC2, anti-SRF, anti-FOXR2 and negative control rabbit IgG) for at least 6 h at 4°C after the pretreatment of agaroses as instructed by the manufacturer, and then the samples were added into above mixture, followed by incubation at 4°C overnight. The next day, the beads were washed with wash buffer 1 (20 mM Tris-HCl pH8, 150 mM NaCl, 2 mM EDTA, 0.1% SDS, 1% Triton X-100), buffer 2 (20 mM Tris-HCl pH8, 500 mM NaCl, 2 mM EDTA, 0.1% SDS, 1% Triton X-100) and buffer 3 (10 mM Tris-HCl pH8, 250 nM LiCl, 2 mM EDTA, 1% NP40), followed by decrosslinking of all samples (including input groups) at 65°C for at least 6 h. After the DNA purification by purification kit (Cat# DP214, TINAGEN, China), qRT-PCR was performed by using the primers in Table S11. The values of qPCR data were quantified as percent of input DNA.

### Quantification and statistical analysis

#### Statistical analysis

The sample size was larger than 10 embryos (n > 10) for WISH and immunofluorescence, 50 embryos for RNA and protein extraction, and 100–150 embryos for FACS in each group. Two to three biological replicates were performed for each assay. Percentage analysis of the results among different groups was performed using hypergeometric distribution in R-console software.^118^ The signals of WISH, WB and immunofluorescence images were quantified using ImageJ software (NIH, Bethesda, Maryland), and the calculated data were analyzed using *t* test by GraphPad Prism 8.0 software. Each dot represents the signal level of a representative image in an individual embryo in each group. The qPCR data were analyzed by one-way analysis of variance (ANOVA) and post hoc Tukey’s test in the IBM Statistic Package for Social Science Statistics for Windows, Version 22 (Released 2013; IBM Corp., USA) software. Each dot represents one repeat.

The number of HSPCs and HSPC proliferation were quantified from the confocal images of the AGM (470 μm × 470 μm) and CHT (470 μm × 470 μm) with z-stacks spanning the entire trunk thickness, and the number of *flk1*^+^*runx1*^+^ and *runx1*^+^*BrdU*^+^ cells were manually counted using ImageJ software. At least ten randomly selected units were analyzed for each control group and experimental group. Also, the fluorescence intensity of acetylated histone protein in a single *runx1*^+^ cell or K562 cell was also quantified from the confocal images (22.15 μm × 22.15 μm) with z-stacks spanning the entire cell thickness, and the fluorescence intensity of histone protein was measured using ImageJ. The statistical data of the signal area and fluorescence level in different samples were analyzed using *t-test* by GraphPad Prism 8 software, with each dot representing the signal level in an individual embryo or one cell in each group. The statistical analysis of the luciferase reporter assay results was performed using GraphPad Prism 8 software (unpaired *t* test). Statistically significant differences among groups are indicated by p < 0.05 (∗), p < 0.01 (∗∗), and p < 0.001 (∗∗∗). A criterion of ≥1.5-fold change (1.5-fold increase/decrease, p value ≤0.05) was used to define obvious/marked changes when comparing differences between groups in this study.

## Supplemental information

Document S1. Figures S1–S20 and Tables S1–S19

Schema 1. The schema of the experimental program in zebrafish, related to all figures
