# Supplementary material for: EPC1/2 regulate hematopoietic stem and progenitor cell proliferation by modulating H3 acetylation and DLST
Source: iScience. 2024 Feb 17;27(3):109263. doi: 10.1016/j.isci.2024.109263 (PMC10910311; doi:10.1016/j.isci.2024.109263)
Supplement: Document S1. Figures S1–S20 and Tables S1–S19 [file mmc1.pdf]

iScience, Volume *27*

Supplemental information

EPC1/2 regulate hematopoietic stem and progenitor cell proliferation by modulating H3 acetylation and DLST

WenYe Liu, Xi Liu, LingYa Li, ZhiPeng Tai, GuoLiang Li, and Jing-Xia Liu

Supplemental figures 1-20

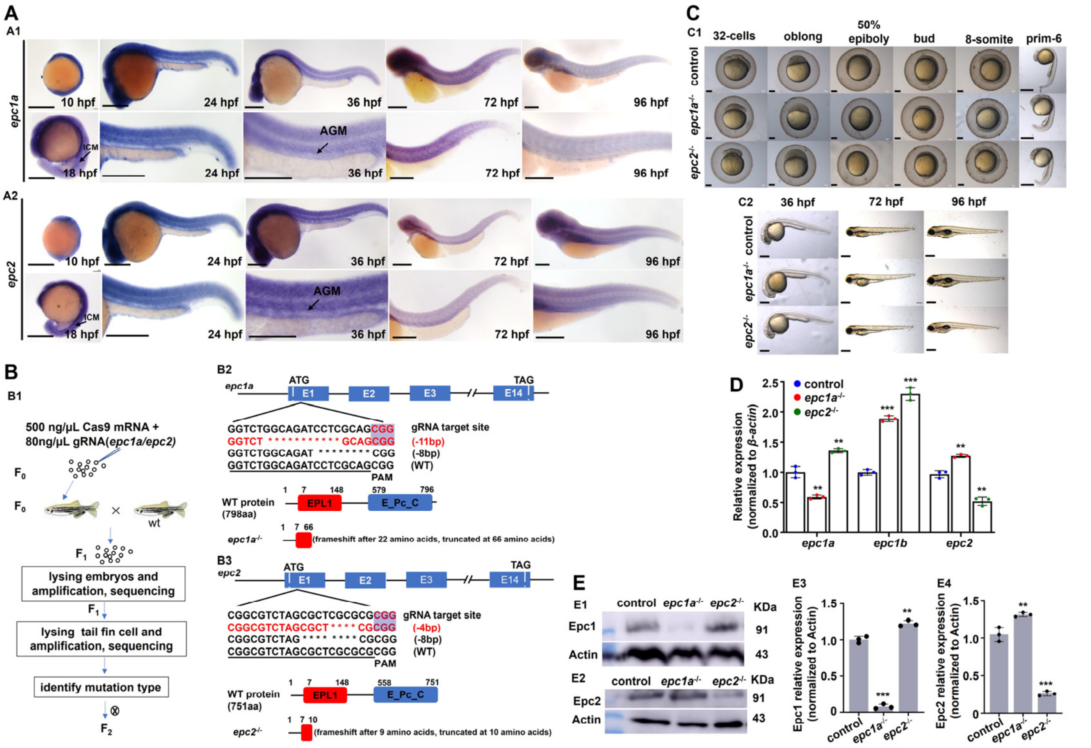

Fig S1

Fig S1. The expression pattern of zebrafish *epc1a* and *epc2*, generation of *epc1a*$^{-/-}$ and *epc2*$^{-/-}$ mutants and the expressions of *epc1a/epc2* mRNA and Epc1/Epc2 protein in mutants, related to Figure 1.

(A) WISH analysis of *epc1a* (**A1**) and *epc2* (**A2**) at 10 hpf, 18 hpf, 24 hpf, 36 hpf, 72 hpf, and 96 hpf, with black arrowheads indicating ICM and AGM regions respectively.

(**B**) Generation of *epc1a*$^{-/-}$ and *epc2*$^{-/-}$ mutants using CRISPR/Cas9. The workflow of zebrafish mutant generation (**B1**), and the schematic diagram of the genomic structure and the genetic mutation of zebrafish *epc1a* (**B2**) and *epc2* (**B3**) used in this study, with ATG for the translation start codon; black lines for introns; blue boxes for exons; asterisks for deletion of 11 bp or 8 bp in *epc1a* gene and 4 bp or 8 bp in *epc2* gene, and mutants of *epc1a* with 11 bp deletion and *epc2* with 4 bp deletion used in this study; numbers for the amino acid positions of domains in Epc1 and Epc2 protein and the length of the *epc1a*$^{-/-}$ and *epc2*$^{-/-}$ mutant proteins.

(C) Images of the control, *epc1a*$^{-/-}$ and *epc2a*$^{-/-}$ embryos or larvae at different developmental stages.

(D) Expression of *epc1a*, *epc1b* and *epc2* in the control, *epc1a*$^{-/-}$ and *epc2*$^{-/-}$ embryos at 33 hpf.

(E) Western blotting analysis of Epc1 (**E1**) and Epc2 (**E2**) protein in the control, *epc1a*$^{-/-}$ and *epc2*$^{-/-}$ embryos, with Actin as the internal control, and quantification results (**E3**, **E4**).

All WISH embryos are shown in lateral view, anterior to the left, and dorsal to the up. Each experiment was repeated three times, and a representative result is shown. Scale bars, 200 μm (**A1, A2**), 500 μm (**C1**, **C2**). Data are presented as mean ± SD (n ≥ 3). t-test, **P* < 0.05, ***P* < 0.01, ****P* < 0.001, NS, not significant.

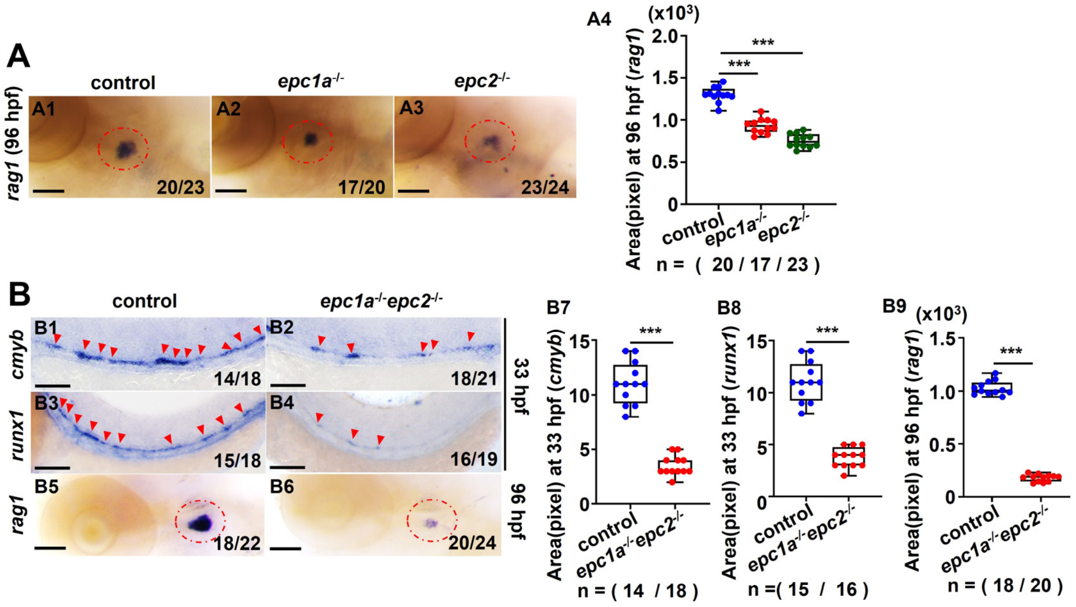

Fig S2

Fig S2. Effects of *epc1a* and *epc2* deficiency on expression of *rag1*, *cmyb* and *runx1*, related to Figure 1.

(A) WISH analysis of *rag1* in the thymus in the control, $epc1a^{-/-}$ and $epc2^{-/-}$ embryos at 96 hpf (**A1-A3**), and quantification of the WISH data (**A4**), with red circles indicating the thymus region.

(B) WISH analysis of *cmyb* (**B1, B2**), *runx1* (**B3, B4**) in the AGM region, and *rag1* (**B5, B6**) in the thymus in control and $epc1a^{-/-}epc2^{-/-}$ mutants. Quantification analysis of the WISH data for *cmyb* (**B7**), *runx1* (**B8**) and *rag1* (**B9**).

All embryos are shown in lateral view, anterior to the left, and dorsal to the up. Each experiment was repeated three times, and a representative result is shown. Data are presented as mean ± SD (n ≥ 3). t-test, Scale bars, 100 μm. **P* < 0.05, ***P* < 0.01, ****P* < 0.001, NS, not significant.

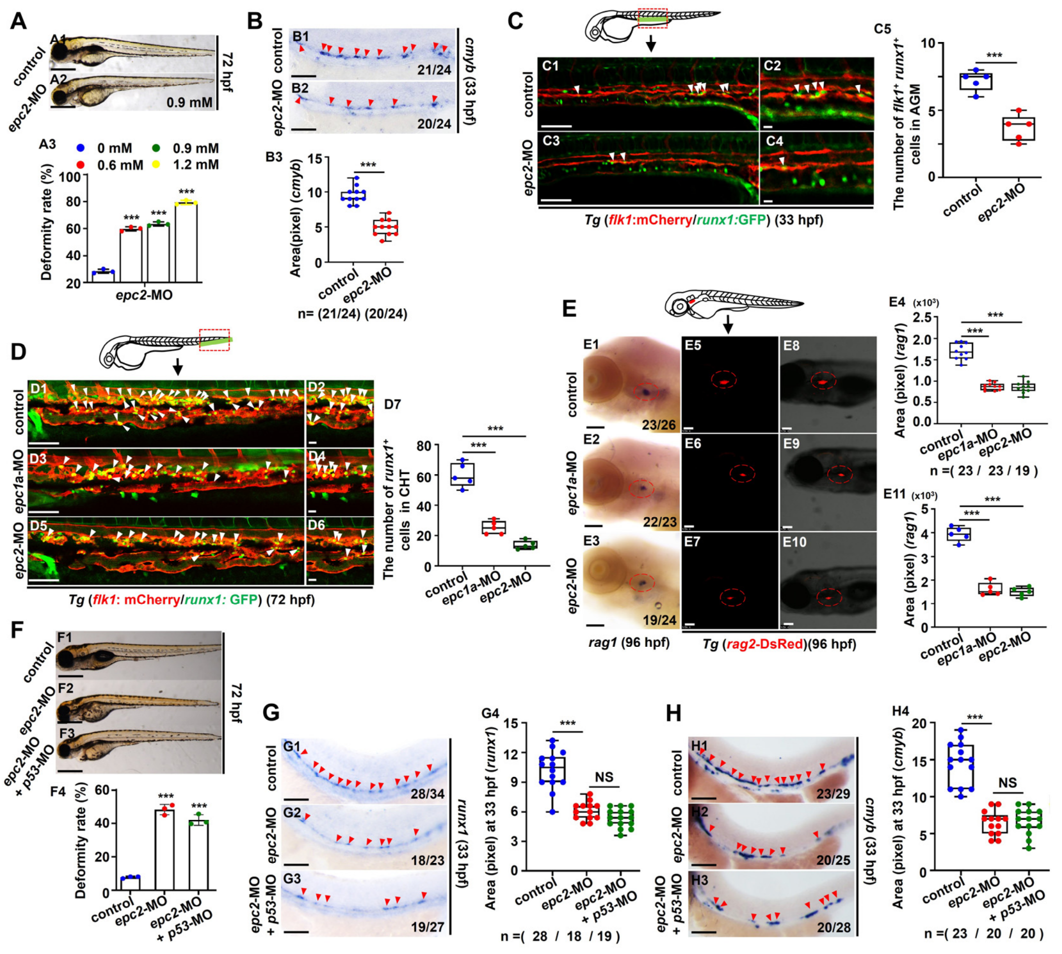

Fig S3

Fig S3. Effects of *epc1a* and *epc2* knockdown on HSPC development, related to Figure 1.

(A) The phenotype of control and *epc2*-MO embryos at 72 dpf (**A1, A2**), and the deformity at different injection concentrations (**A3**).

(B) WISH analysis of *cmyb* in the AGM region in control and *epc2*-MO embryos at 33 hpf (**B1, B2**), and quantification of the WISH data (**B3**), with red arrowheads indicating positive signals.

(C) Confocal images of the AGM region in *Tg* (*flk1*: mCherry/*runx1*: GFP) embryos injected with *epc2*-MO at 33 hpf, with white arrowheads indicating double-positive cells. **C2** and **C4** present the magnified views of **C1** and **C3**, respectively.

(D) Confocal images of the CHT in *Tg* (*flk1*: mCherry/*runx1*: GFP) embryos injected with *epc1a*-MO and *epc2*-MO at 72 hpf, with white arrowheads indicating double-positive cells. **D2**, **D4** and **D6** show the magnified views of **D1**, **D3** and **D5**, respectively.

(E) The expressions of *rag1* and *rag2* in the thymus in control, *epc1a*-MO and *epc2*-MO larvae at 96 hpf by WISH analysis (**E1-E3**) and confocal microscopy (**E5-E10**), and quantification of the WISH data (**E4**) and confocal images (**E11**), with red circles indicating the thymus region.

(F) Images for the control, *epc2* MO-injected, and *epc2* MO plus *p53* MO-coinjected embryos at 72 dpf (**F1-F3**), and the deformity at different groups (**F4**).

(G) WISH analysis of *runx1* in the control, *epc2* MO-injected, and *epc2* MO plus *p53* MO-coinjected embryos (**G1-G3**), and quantification of the WISH data (**G4**), with red arrowheads indicating positive signals.

(H) WISH analysis of *cmyb* in the AGM region in the control, *epc2* MO-injected, and *epc2* MO

plus *p53* MO-coinjected embryos (**H1-H3**). Quantification analysis of the WISH data (**H4**), with red arrowheads indicating positive signals.

Each experiment was repeated three times, and a representative result is shown. All embryos are shown in lateral view, anterior to the left, and dorsal to the up. Data are presented as mean ± SD ($n \geq 3$). Scale bars, 500 μm (**A1-A2, F1-F3**), 100 μm (**B1-B2, C1-C4, D1-D6, E1-E10, G1-G3 and H1-H3**). t-test, **P* < 0.05, ***P* < 0.01, ****P* < 0.001, NS, not significant.

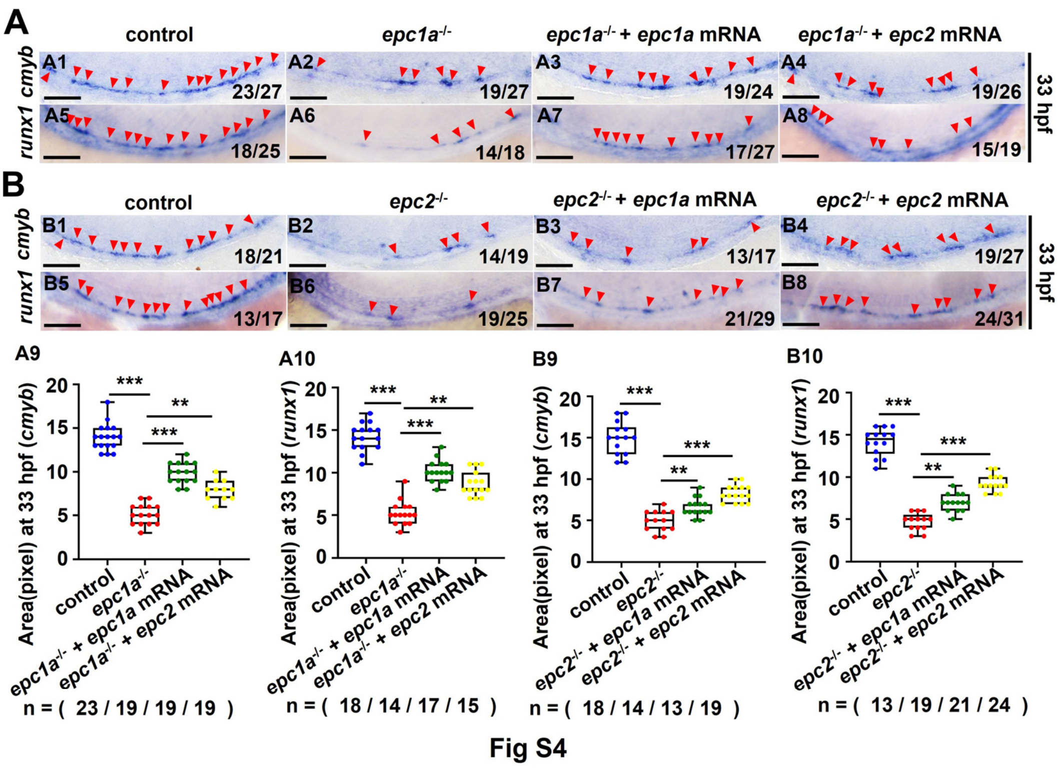

Fig S4. Ectopic expression of *epc1a* or *epc2* mRNA partially restored the decrease of *runx1/cmyb* in the AGM region in mutants, related to Figure 1.

(A) WISH analysis of *cmyb* and *runx1* in the AGM region in the control, *epc1a*$^{-/-}$, *epc1a* mRNA-injected *epc1a*$^{-/-}$, and *epc2* mRNA-injected *epc1a*$^{-/-}$ embryos (**A1-A8**), and quantification of the WISH data (**A9**, **A10**).

(B) WISH analysis of *cmyb* and *runx1* in AGM region in control, *epc2*$^{-/-}$, *epc1a* mRNA-injected *epc2*$^{-/-}$, and *epc2* mRNA-injected *epc2*$^{-/-}$ embryos (**B1-B8**), and quantification of the WISH data (**B9**, **B10**), with red arrowheads indicating positive signals.

Each experiment was repeated three times, and a representative result is shown. All embryos are shown in lateral view, anterior to the left, and dorsal to the up. Data are presented as mean ± SD ($n \geq 3$). t-test, Scale bars, 100 μm. **P* < 0.05, ***P* < 0.01, ****P* < 0.001, NS, not significant.

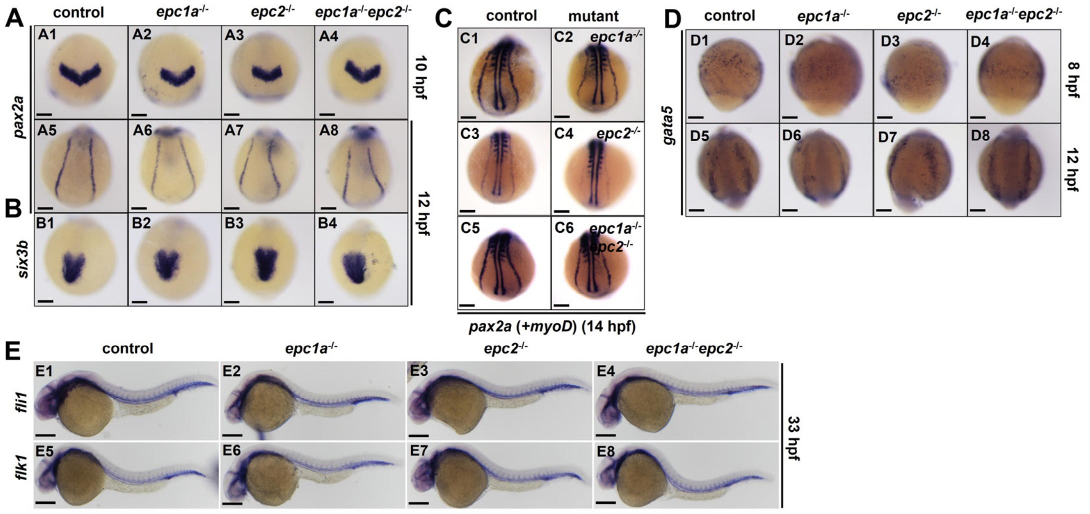

Fig S5

Fig S5. The expression of different maker genes in the control, *epc1a*$^{-/-}$, *epc2*$^{-/-}$, *epc1a*$^{-/-}$*epc2*$^{-/-}$ embryos or larvae, related to Figure 1.

(A) WISH analysis of lateral plate mesoderm marker *pax2a* in the control, *epc1a*$^{-/-}$, *epc2*$^{-/-}$ and *epc1a*$^{-/-}$*epc2*$^{-/-}$ embryos at 10 hpf and 12 hpf.

(B) WISH analysis of neuro-ectoderm marker *six3b* in control, *epc1a*$^{-/-}$, *epc2*$^{-/-}$ and *epc1a*$^{-/-}$*epc2*$^{-/-}$ embryos at 12 hpf.

(C) WISH analysis of *pax2a* (+*myoD*) in the control, *epc1a*$^{-/-}$, *epc2*$^{-/-}$ and *epc1a*$^{-/-}$*epc2*$^{-/-}$embryos at 14 hpf.

(D) WISH analysis of endoderm marker *gata5* in the control, *epc1a*$^{-/-}$, *epc2*$^{-/-}$ and *epc1a*$^{-/-}$*epc2*$^{-/-}$ embryos at 8 hpf and 12 hpf.

(E) WISH analysis of *fli1* and *flk1* in the control, *epc1a*$^{-/-}$, *epc2*$^{-/-}$ and *epc1a*$^{-/-}$*epc2*$^{-/-}$ embryos at 33 hpf.

Each experiment was repeated three times, and a representative result is shown. **A1-A8, B1-B4, C1-C6** and **D1-D8,** dorsal view, anterior to the up; **E1-E8**, lateral view, anterior to the left, Scale bars 100 μm.

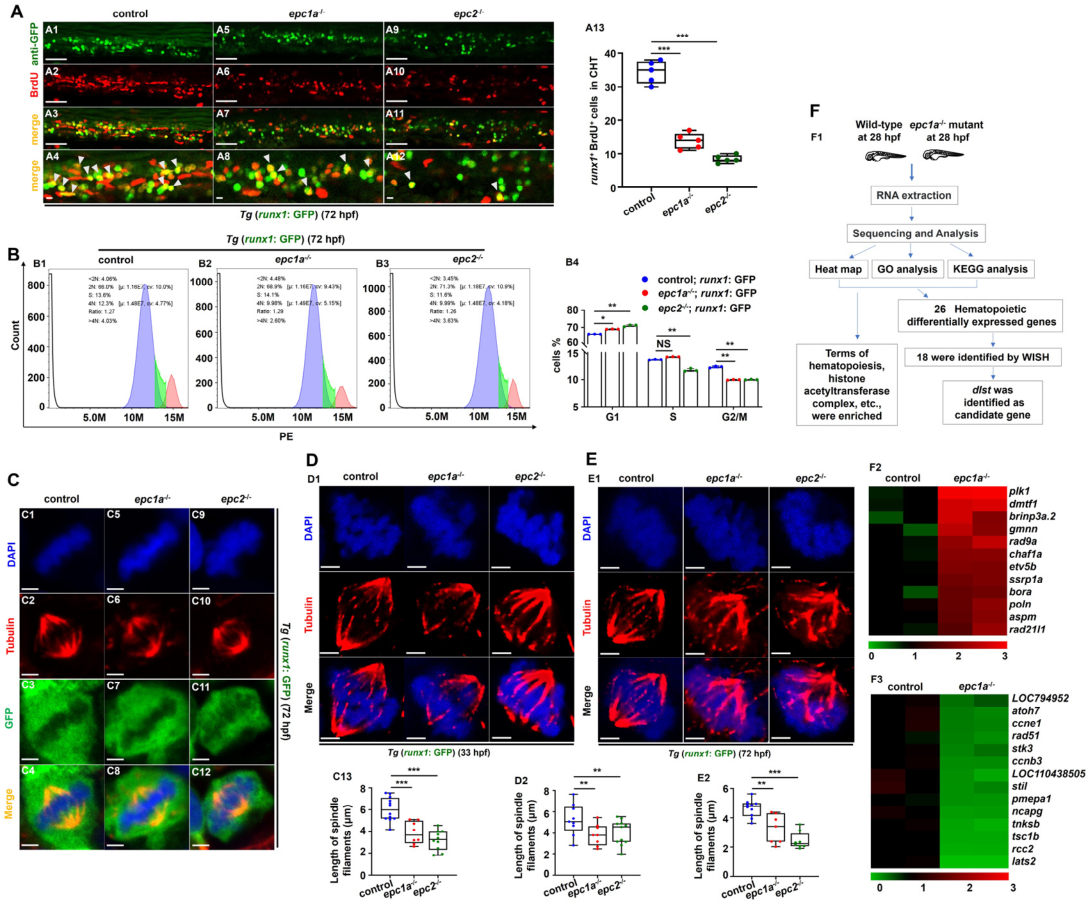

Fig S6. Effects of *epc1a* and *epc2* deficiency on cell proliferation, cell cycle, and mitosis, related to Figure 2.

(A) Double staining of *runx1*$^{+}$(GFP) and BrdU in the CHT in the control, *epc1a*$^{-/-}$ and *epc2*$^{-/-}$ embryos at 72 hpf **(A1-A12)**, and quantification of *runx1*$^{+}$BrdU^{+} cells (**A13**), with white arrowheads indicating double-positive cells. **A4**, **A8** and **A12** present the magnified views of **A3**, **A7** and **A11**, respectively.

(B) Analysis of cell cycle in *runx1*-positive cells sorted from *Tg* (*runx1*: GFP) (control), *Tg* (*epc1a*$^{-/-}$; *runx1*: GFP) and *Tg* (*epc2*$^{-/-}$; *runx1*: GFP) at 72 hpf (**B1-B3**), and the quantification of the percentage of cells in different phases (**B4**).

(C) Double staining of *runx1*$^{+}$ (GFP) and α-Tubulin in the CHT region in the control, *epc1a*$^{-/-}$ and *epc2*$^{-/-}$ embryos at 72 hpf (**C1-C12**), and quantification of length of spindle filaments (**C13**).

(D, E) Mitotic malformation of non-*runx1*$^{+}$ cells in AGM (**D1**) and CHT regions (**E1**) in the control, *epc1a*$^{-/-}$ and *epc2*$^{-/-}$ mutants at 33 hpf and 72 hpf, and quantification of length of spindle filaments (**D2, E2**).

(F) Schematic representation of RNA-Seq analysis workflow (**F1**) and heat map for differentially expressed hematopoietic genes in the control and *epc1a*$^{-/-}$ embryos based on RNA-Seq data (**F2, F3**).

Each experiment was repeated three times, and a representative result is shown. All embryos are shown in lateral view, anterior to the left, and dorsal to the up. Scale bars, 100 µm (**A1-A12**) and 2 µm (**C1-C12, D-E**), Data are presented as mean ± SD (n ≥ 3). t-test, **P* < 0.05, ***P* < 0.01, ****P* < 0.001, NS, not significant.

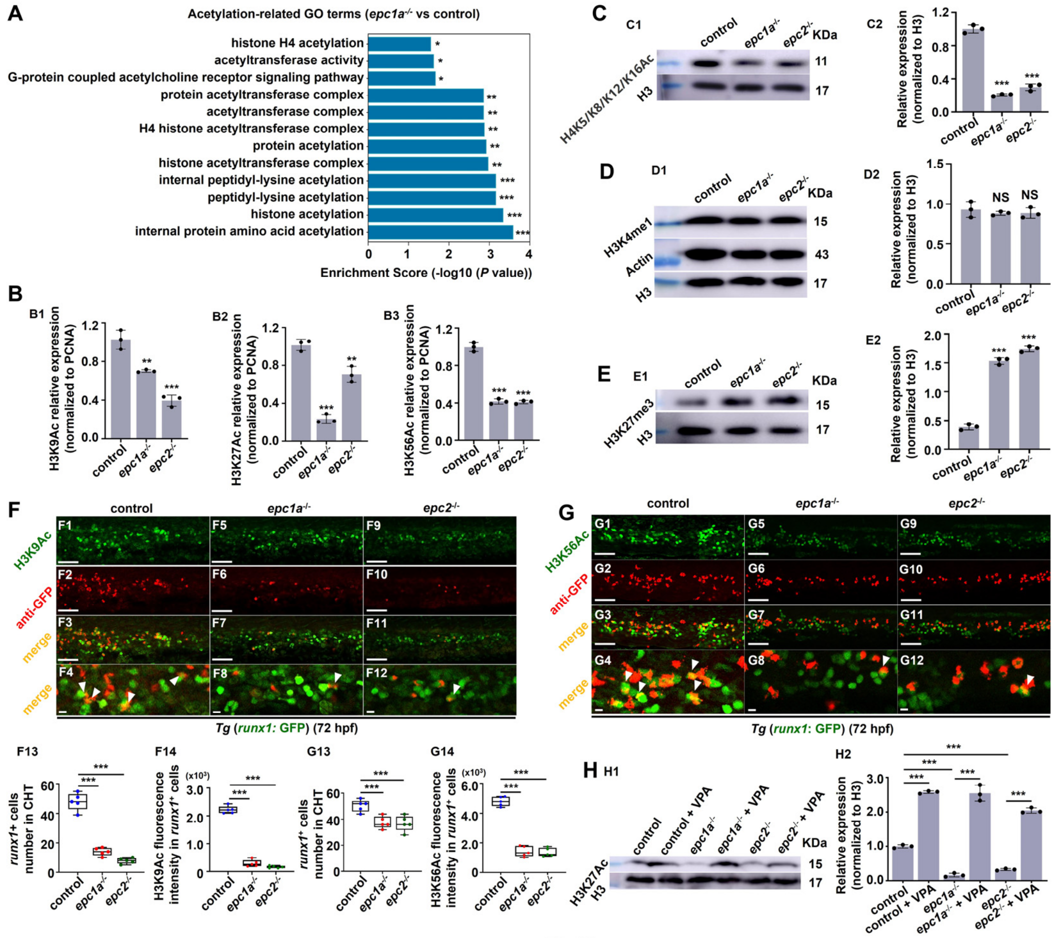

Fig S7

Fig S7. Effects of *epc1a* and *epc2* deficiency on H3 or H4 acetylated protein, related to Figures 2 and 3.

(A) Enriched GO terms for acetylation-related DEGs in *epc1a*$^{-/-}$ mutants.

(B) The quantification of H3K9Ac (**B1**), H3K27Ac (**B2**) and H3K56Ac (**B3**) proteins in control, *epc1a*$^{-/-}$ and *epc2*$^{-/-}$ embryos.

(C) H4K5/K8/K12/K16Ac protein level in the control, *epc1a*$^{-/-}$ and *epc2*$^{-/-}$ embryos at 33 hpf (**C1**), and quantification analysis (**C2**).

(**D, E**) Western blotting analysis of H3K4me1 (**D1**) and H3K27me3 (**E1**) in the control, *epc1a*$^{-/-}$ and *epc2*$^{-/-}$ embryos, with Actin and PCNA as internal controls, and quantification of H3K4me1 (**D2**) and H3K27me3 (**E2**).

(F, G) Double staining of *runx1*-GFP with anti-H3K9Ac (**F1-F12**) and H3K56Ac (**G1-G12**) in the CHT in the control, *epc1a*$^{-/-}$ and *epc2*$^{-/-}$ embryos at 72 hpf, and quantification of *runx1*$^{+}$ cells (**F13, G13**), *runx1*$^{+}$H3K9Ac^{+} (**F14**) and *runx1*$^{+}$H3K56Ac^{+} cells (**G14**), with white arrowheads indicating double-positive cells. **F4, F8**, **F12**, **G4**, **G8** and **G12** show the magnified images of **F3**, **F7**, **F11**, **G3**, **G7** and **G11**, respectively.

(H) Protein level of H3K27Ac in the control, *epc1a*$^{-/-}$ and *epc2*$^{-/-}$ embryos and the corresponding groups treated with VPA (**H1**), and quantification analysis (**H2**). Each experiment was repeated three times, and a representative result is shown. All embryos are shown in lateral view, anterior to the left, and dorsal to the up. Scale bars, 100 µm (**F1-F12, G1-G12**). Data are presented as mean ± SD ($n \geq 3$). t-test, *$P < 0.05$, **$P < 0.01$, ***$P < 0.001$, NS, not significant.

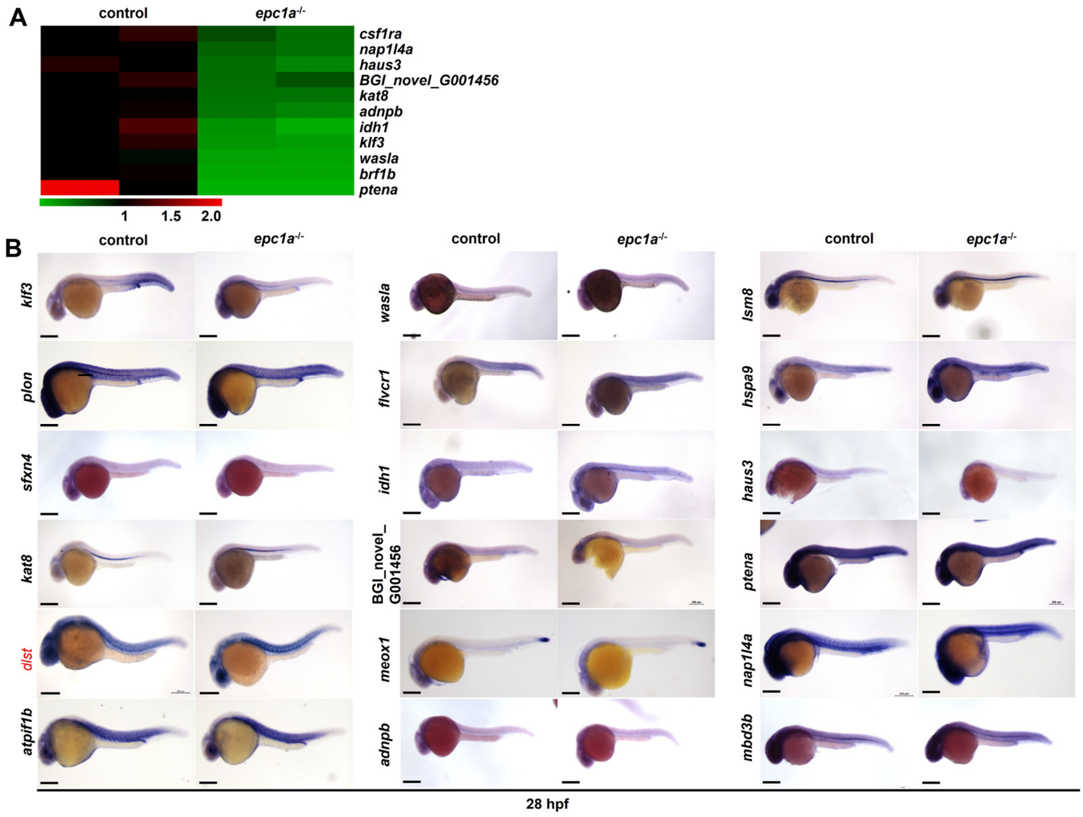

Fig S8

Fig S8. Heat map and WISH analysis of hematopoietic genes in the control and *epc1a*$^{-/-}$ embryos, related to Figure 3.

(A) Heat map for down-regulated hematopoietic genes in the control and *epc1a*$^{-/-}$ embryos based on RNA-Seq data.

(B) WISH expression analysis of DEGs related to hematopoietic development in the AGM region in the control and *epc1a*$^{-/-}$ embryos at 28 hpf. All embryos are shown in lateral view, anterior to the left, and dorsal to the up. Scale bars, 200 μm.

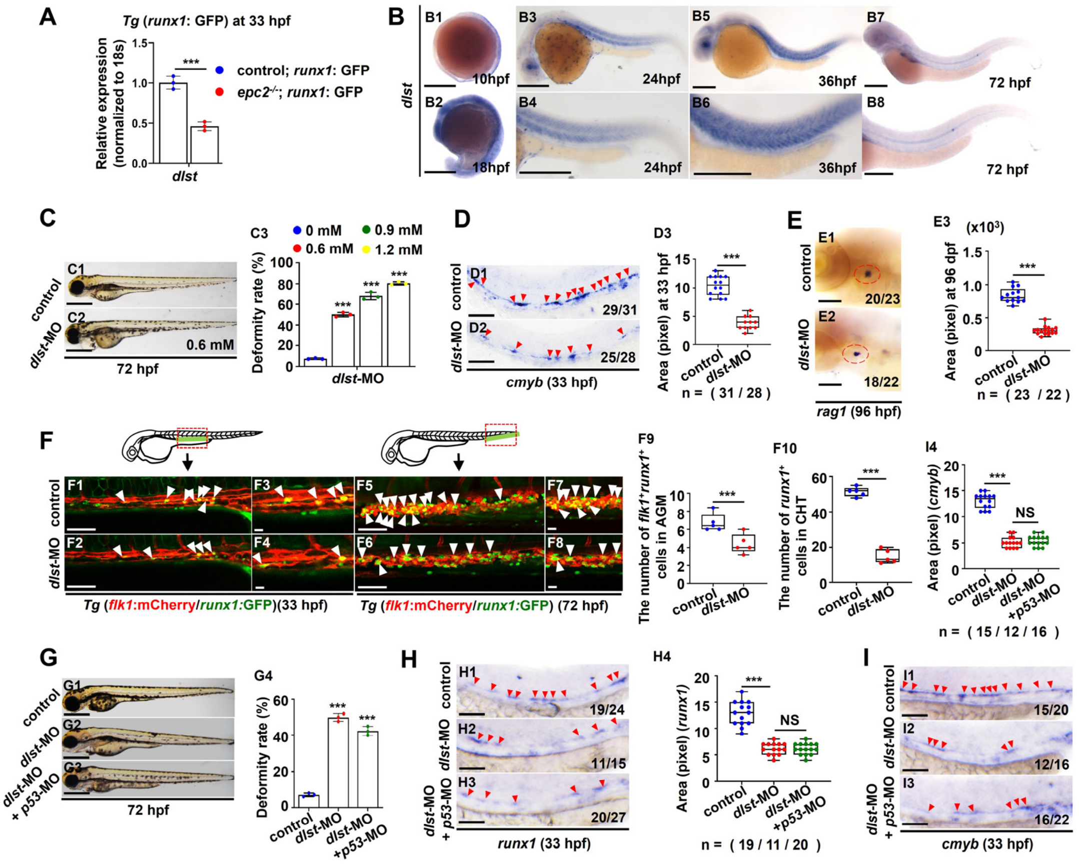

Fig S9

Fig S9. Effects of *dlst* knockdown on HSPC development, related to Figure 4.

(A) The expression of *dlst* in *runx1*$^{+}$ cells sorted from *Tg* (*runx1*: GFP) and *Tg* (*epc2*$^{-/-}$; *runx1*: GFP) embryos at 33 hpf.

(B) WISH analysis for *dlst* expression at 10 hpf, 18 hpf, 24 hpf, 36 hpf, and 96 hpf.

(C) The phenotype of the control and *dlst*-MO embryos at 72 dpf (**C1**, **C2**), and the deformity at different injection concentrations (**C3**).

(D, E) WISH analysis of *rag1* (**D1, D2**) and *cmyb* (**E1, E2**) in control and *dlst*-MO embryos, and quantification of the WISH data for *rag1* (**D3**) and *cmyb* (**E3**), with red arrowheads indicating positive signals and red circles indicating the thymus region.

(F) Confocal images of the AGM in *Tg* (*flk1:* mCherry/*runx1:* GFP) embryos injected with *dlst*-MO at 33 hpf and 72 hpf, and quantification of *flk1*$^{+}$*runx1*$^{+}$ (**F9**) and *runx1*$^{+}$ cells (**F10**), with white arrowheads indicating double-positive cells. **F3**, **F4** and **F7**, **F8** present the magnified views of **F1**, **F2** and **F5**, **F6**, respectively.

(G) The phenotype of the control, *dlst* MO-injected, and *dlst* MO and *p53* MO-coinjected embryos at 72 dpf.

(H, I) WISH analysis of *runx1* (**H1-H3**) and *cmyb* (**I1-I3**) in the control, *dlst* MO-injected, and *dlst* MO plus *p53* MO-coinjected embryos at 33 hpf, and quantification of the WISH data for *runx1*(**H4**) and *cmyb* (**I4**), respectively, with red arrowheads indicating double-positive cells.

All embryos are shown in lateral view, anterior to the left, and dorsal to the up. Scale bars,500 µm (**C1-C2, G1-G3**), 200 µm (**B1-B8**) and 100 µm (**D1-D2, E1-E2, F1-F8, H1-H3, I1-I**). Data are presented as mean ± SD (n ≥ 3). t-test, *$P < 0.05$, **$P < 0.01$, ***$P < 0.001$. NS, not significant.

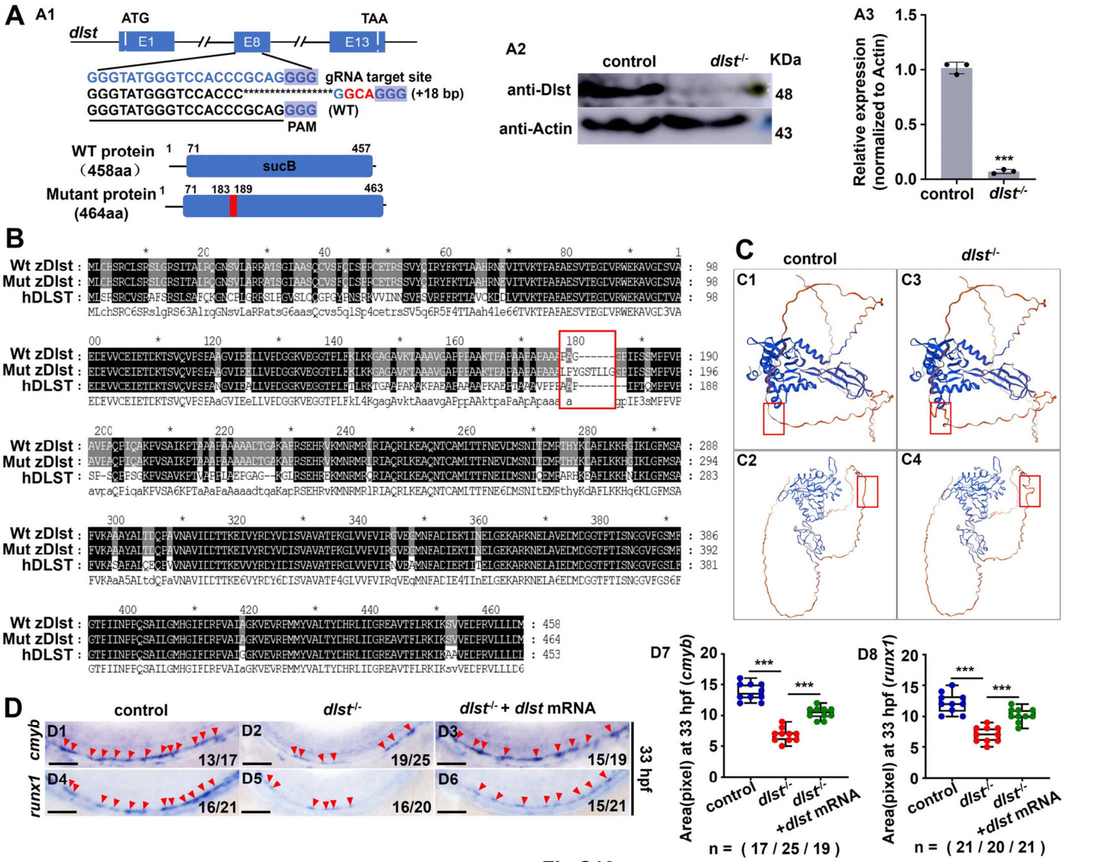

Fig S10

Fig S10. The generation of *dlst*$^{-/-}$ mutant and rescue of HSPC defect by *dlst* mRNA in *dlst*$^{-/-}$ embryos, related to Figure 4.

(A) Schematic diagram for the genomic structure and the genetic mutation of zebrafish *dlst* gene, with ATG for the translation start codon; black lines for introns; blue boxes for exons; asterisks for insertion of 18 bp in *dlst* gene; numbers for the amino acid positions of domains in Dlst protein and the length of the *dlst*$^{-/-}$ mutant protein (**A1**). Western blotting analysis of Dlst protein in the control and *dlst*$^{-/-}$ embryos, with Actin as the internal control (**A2**), and quantification for Dlst protein (**A3**).

(**B**) Multiple sequence alignment of WT Dlst and mutant Dlst with human DLS, and the mutant amino acid is shown in red box.

(**C**) Three-dimensional (3D) models of WT Dlst (**C1-C2**) and mutant Dlst protein (**C3-C4**) predicted by SWISS Model.

(D) WISH analysis of *cmyb* (**D1-D3**) and *runx1* **(D4-D6)** in the AGM region in the control, *dlst*$^{-/-}$ and *dlst* mRNA-injected *dlst*$^{-/-}$ embryos, and quantification of the WISH data for *cmyb* (**D7**) and *runx1* (**D8**), with red arrowheads indicating positive signals.

Each experiment was repeated three times, and a representative result is shown. All embryos are shown in lateral view, anterior to the left, and dorsal to the up. Scale bars, 100 µm. Data are presented as mean ± SD ($n \geq 3$). t-test, *$P < 0.05$, **$P < 0.01$, ***$P < 0.001$, NS, not significant.

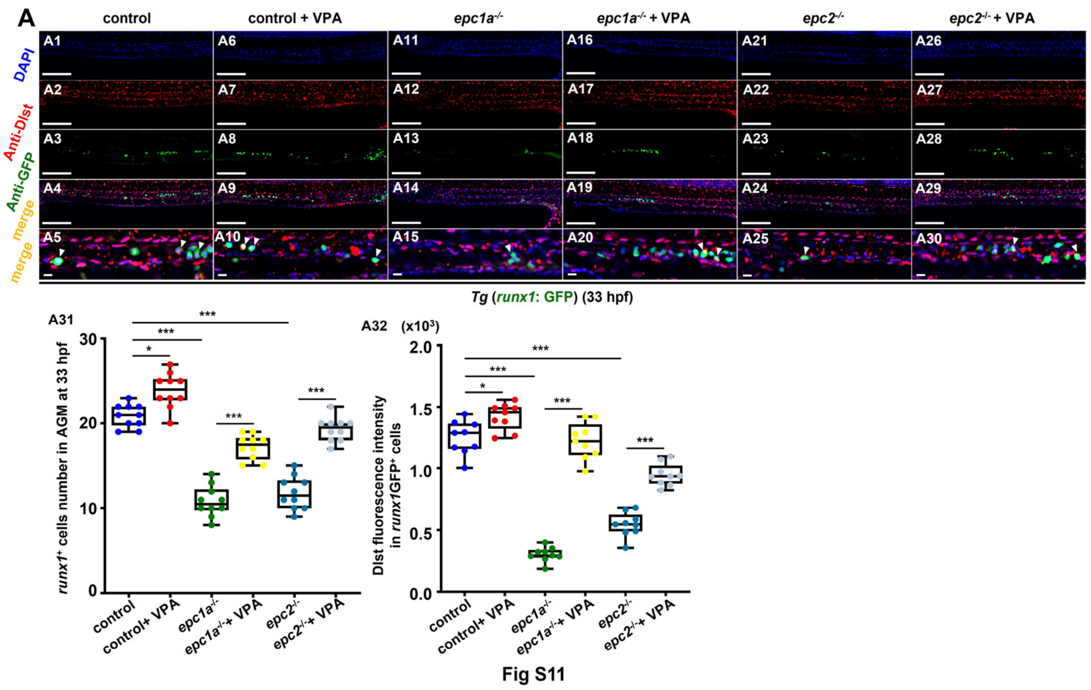

Fig S11. Effects of VPA treatment on Dlst expression in *runx1*GFP$^+$ cells in the control, *epc1a*$^{-/-}$, *epc2*$^{-/-}$ embryos at 33 hpf, related to Figure 4.

(A) Double staining of *runx1*$^+$ (GFP) and Dlst in the AGM region in the control, *epc1a*$^{-/-}$, *epc2*$^{-/-}$ embryos and the corresponding groups treated with VPA at 33 hpf (**A1-A30**), and quantification of *runx1*$^+$ cells (**A31**) *runx1*$^+$Dlst$^+$ cells (**A32**), with white arrowheads indicating double-positive cells. **A5, A10, A15, A20 A25** and **A30** show the magnified views of **A4, A9, A14, A24** and **A29**, respectively. Each experiment was repeated three times, and a representative result is shown. All embryos are shown in lateral view, anterior to the left, and dorsal to the up. Scale bars, 100 μm. Data are presented as mean ± SD (n ≥ 3). t-test, *P < 0.05, **P < 0.01, ***P < 0.001, NS, not significant.

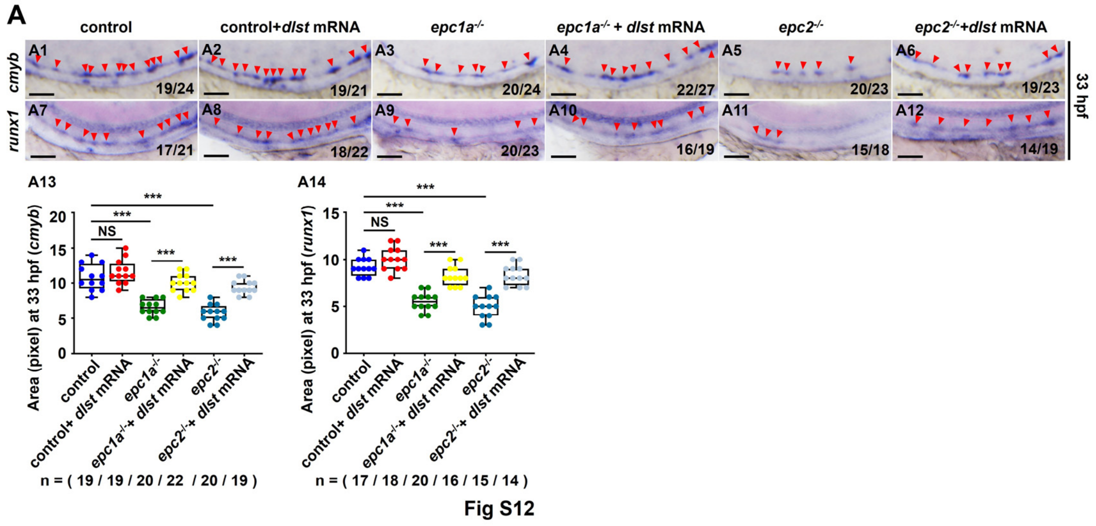

Fig S12 Ectopic expression of *dlst* mRNA partially restored the decrease of *cmyb/runx1* in *epc1a*$^{-/-}$ and *epc2*$^{-/-}$ mutants, related to Figure 4.

(A) WISH analysis of *cmyb* and *runx1* in the AGM region in the control, *epc1a*$^{-/-}$, *epc2*$^{-/-}$ and the corresponding groups injected with *dlst* mRNA, and quantification of the WISH data (**A13, A14**), with red arrowheads indicating positive signals. Each experiment was repeated three times, and a representative result is shown. All embryos are shown in lateral view, anterior to the left, and dorsal to the up. Scale bars, 100 μm. Data are presented as mean ± SD (n ≥ 3). t-test, **P* < 0.05, ***P* < 0.01, ****P* < 0.001, NS, not significant.

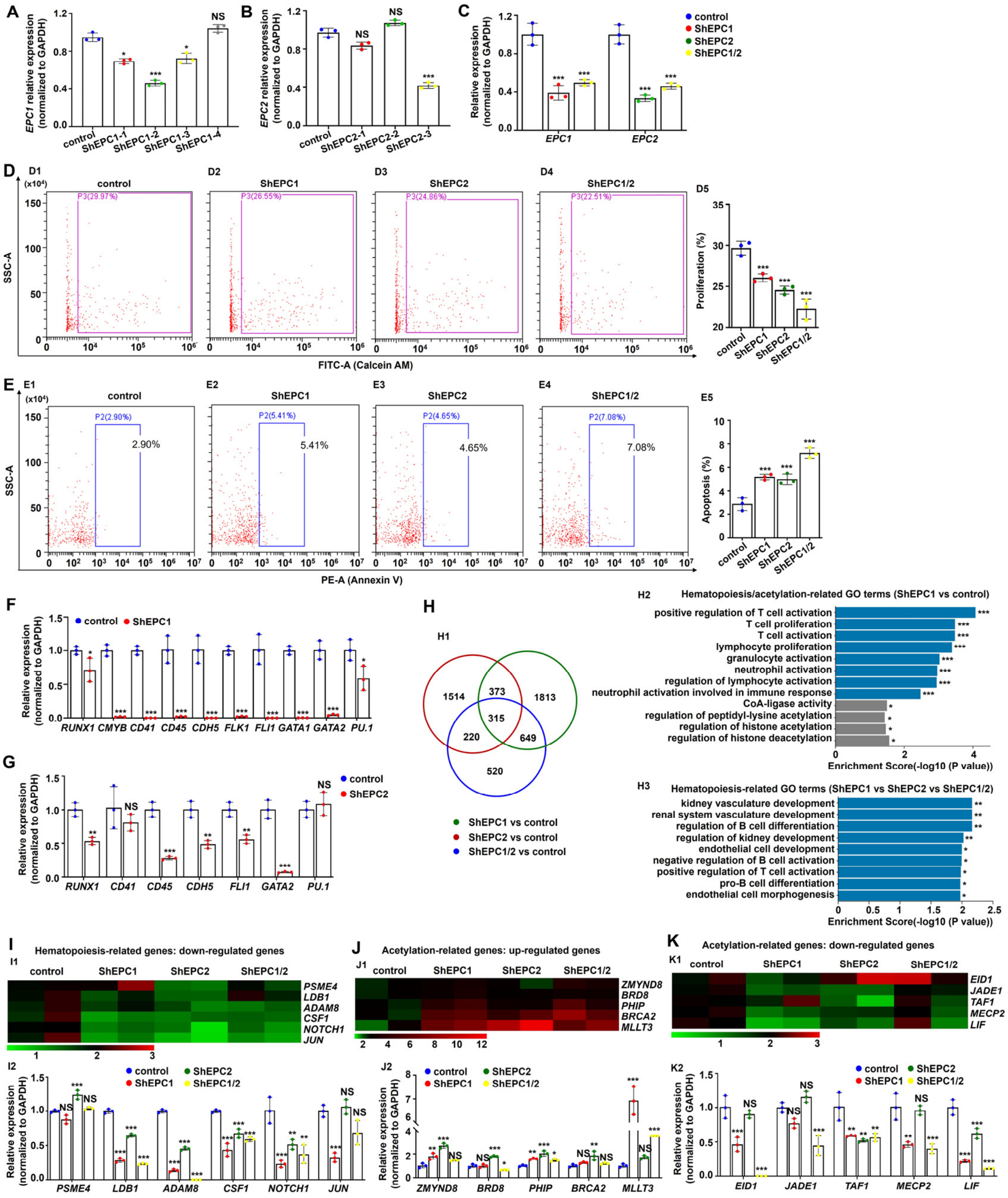

Fig.S13

Fig S13 Roles of EPC1 and EPC2 are conserved in human hematopoietic cells, related to Figure 5.

(A, B) The small RNAi screen for effective ShRNAs for targeting *EPC1* (**A**) or *EPC2* (**B**), respectively, and ShEPC1-2 targeting *EPC1* and ShEPC2-3 targeting *EPC2* as stable EPC1//EPC2 deficient cells were named ShEPC1 and ShEPC2 in this study respectively**.**

(C) qRT–PCR expression analysis of *EPC1* and *EPC2* in control, ShEPC1, ShEPC1 and ShEPC1/2 K562 cells, respectively.

(D, E) The percentage of proliferation (**D**) and apoptosis (**E**) in control, ShEPC1, ShEPC2, and ShEPC1/2 cells.

(F) qRT-PCR expression analysis of *RUNX1*, *CMYB*, *CD41*, *CD45*, *CDH5*, *FLK1*, *FLI1*, *GATA1*, *GATA2* and *PU.1* in control and ShEPC1.

(G) qRT-PCR expression analysis of *Runx1*, *CD41*, *CD45*, *CDH5*, *FLI1*, *GATA2* and *PU.1* in control and ShEPC2.

(H) Venn plot analysis of the overlap of genes between control, ShEPC1, ShEPC1 and ShEPC1/2 K562 cells based on RNA-Seq data (**H1**), GO analysis of DEGs involved in acetylation and hematopoiesis in control and ShEPC1 K562 cells (**H2**), and GO analysis ofoverlapping DEGs involved in hematopoiesis (**H3**).

(I-K) Heat map for differentially expressed hematopoietic genes (*PSME4*, *LDB1*, *ADAM8*, *CSF1*, *NOTCH1* and *JUN*) (**I1**), acetylation-related genes (*ZMYND8*, *BRD8*, *PHIP*, *BRCA2*, *MLLT3*, *EID1*, *JADE1*, *TAF1*, *MECP2* and *LIF*) (**J1-K1**) in control, ShEPC1, ShEPC2 and ShEPC1/2 cells based on RNA-Seq data, and qRT-PCR verification (**I2, J2, K2**).

Data are presented as mean ± SD ($n \geq 3$). t-test, **P* < 0.05, ***P* < 0.01, ****P* < 0.001. NS, not significant.

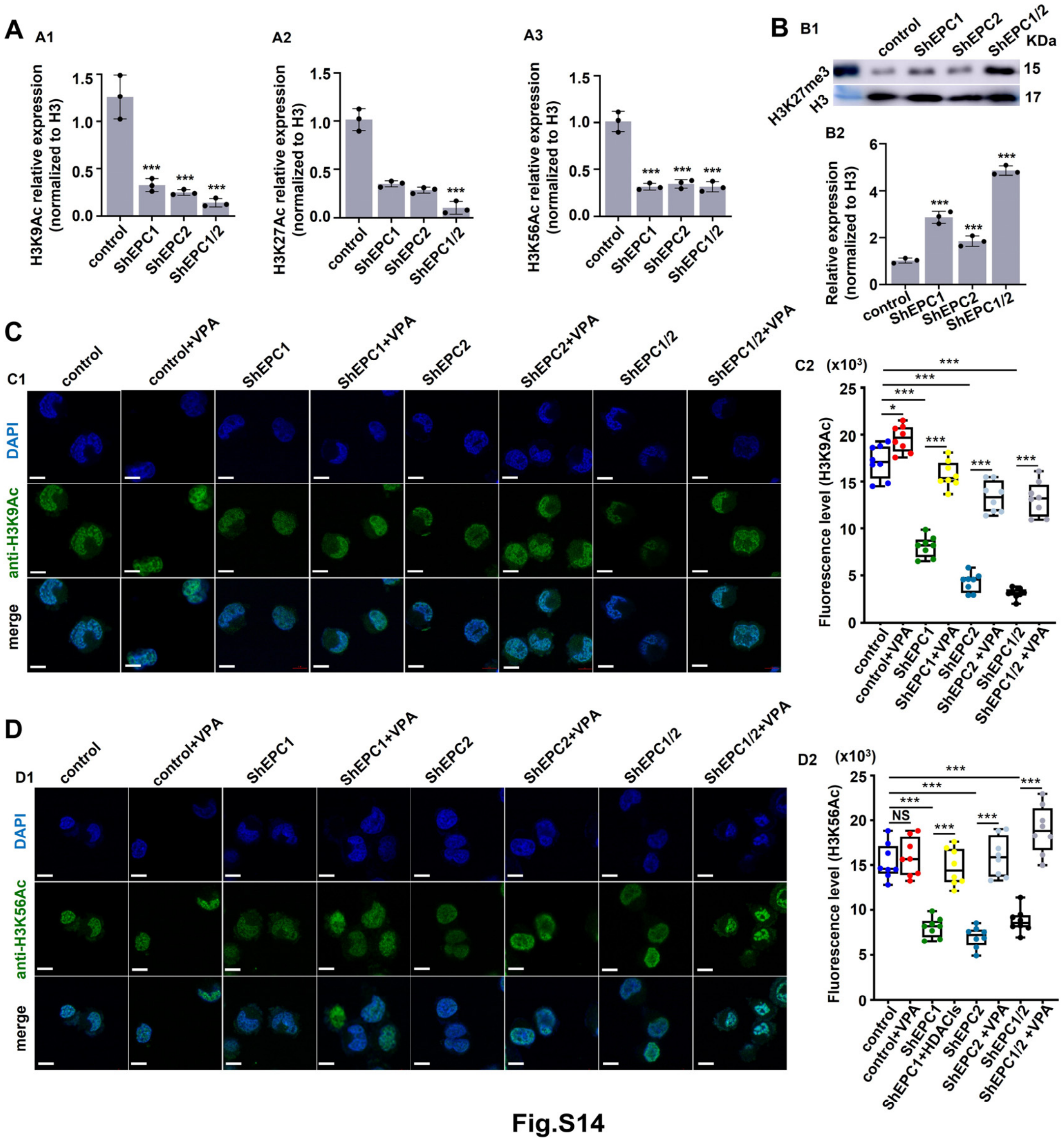

Fig S14 Effects of *EPC1* and *EPC2* knockdown on H3 acetylation and methylation, related to Figure 5.

(A) The quantification of H3K9Ac (**A1**), H3K27Ac (**A2**) and H3K56Ac (**A3**) proteins in control, ShEPC1, ShEPC2 and ShEPC1/2 K562 cells.

(**B**) Western blotting analysis of H3K27me3 protein in control, ShEPC1, ShEPC2 and ShEPC1/2 cells, with H3 as internal control, and quantification of H3K27me3 (**B2**).

(C, D) Immunofluorescence analysis of control, ShEPC1, ShEPC2 and ShEPC1/2 K562 cells, as well as the corresponding groups treated with HDACIs using the anti-H3K9Ac (**C1**) and anti-H2K56Ac (**D1**) antibodies. Quantitative analysis for the fluorescence intensity of H3K9Ac (**C2**) and H3K56Ac (**D2**).

Each experiment was repeated three times, and a representative result is shown. Scale bars, 5 μm. Data are presented as mean ± SD (n ≥ 3). t-test, *$P < 0.05$, **$P < 0.01$, ***$P < 0.001$. NS, not significant.

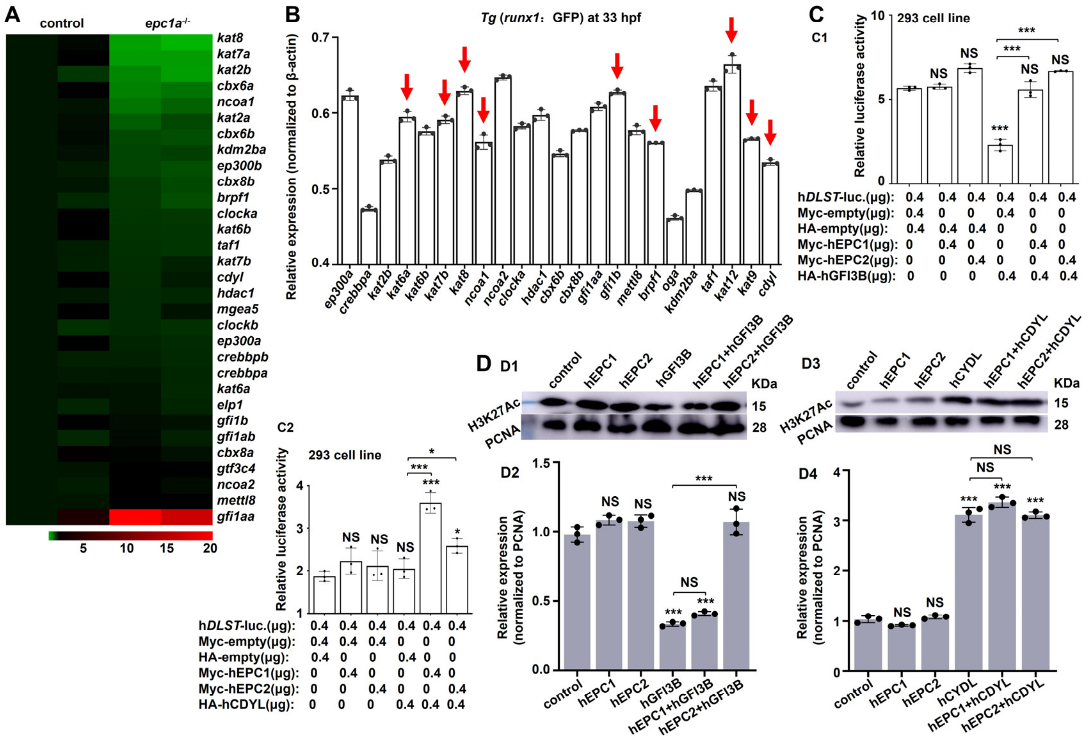

Fig S15

Fig S15. EPC1 and EPC2 modulate histone acetylation, related to Figure 6.

(A) Heat map for differentially expressed acetylation-related genes in the control and *epc1a*$^{-/-}$ embryos based on RNA-Seq data.

(B) qRT-PCR expression analysis of acetylation-related genes in *runx1*$^{+}$ cells sorted from *Tg* (*runx1*: GFP) at 33 hpf, with red arrowheads indicating the genes verified by luciferase reporter assays in this study.

(C) The transcriptional activities of the *DLST* promoter through co-transfection of EPC1 and EPC2 with hGFI3B (**C1**), and hCDYL (**C2**) in 293T cells, respectively.

(D) Western blotting analysis of hGFI3B (**D1**) and hCDYL (**D3**) in the co-transfected cells, with PCNA as the internal controls, and quantification of corresponding protein (**D2** and **D4**).

Each experiment was repeated three times, and a representative result is shown. Data are presented as mean ± SD (n ≥ 3). t-test, **P* < 0.05, ***P* < 0.01, ****P* < 0.001. NS, not significant.

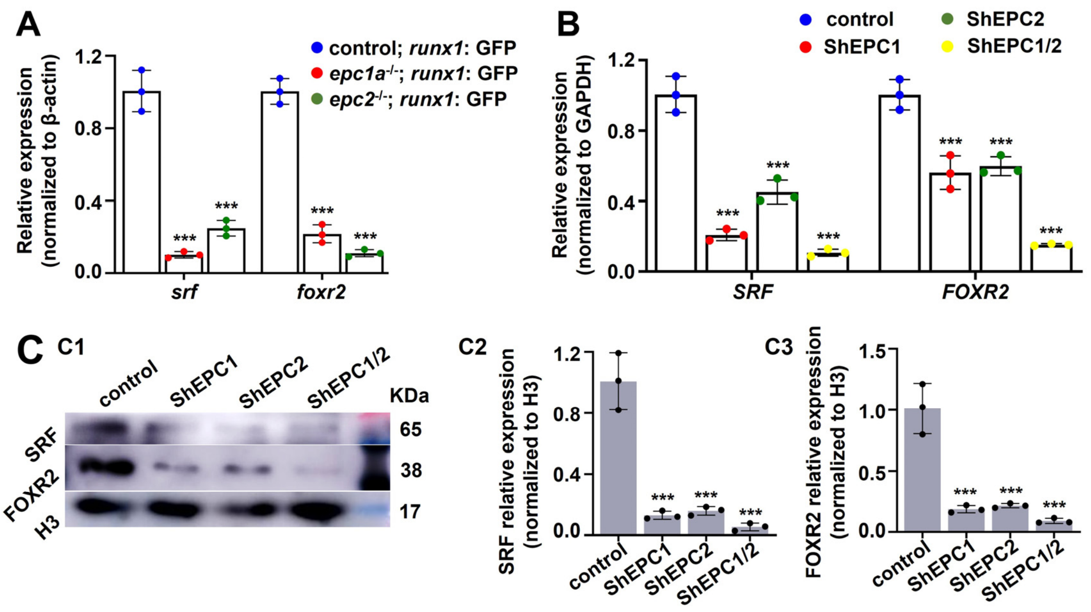

Fig. S16

Fig S16. Decreased expressions of SRF and FOXR2 in HSPCs and K562 cells with EPC1 or EPC2-deficiency, related to Figure 7.

(**A**) Expression of *srf* and *foxr2* in *runx1*$^{+}$ cells sorted from *Tg* (*runx1*: GFP) (control), *Tg* (*epc1a*$^{-/-}$; *runx1*: GFP) and *Tg* (*epc2*$^{-/-}$; *runx1*: GFP) at 33 hpf by One Step Cell-Direct qRT-PCR.

(**B**) Expression of SRF and FOXR2 in control, ShEPC1, ShEPC2, and ShEPC1/2 cells by qRT-PCR.

(**C**) Western blotting analysis of SRF and FOXR2 proteins in control, ShEPC1, ShEPC2 and ShEPC1/2 cells (**C1**), with H3 as internal control, and quantification of SRF (**C2**) and FOXR2 (**C2**). Each experiment was repeated three times, and a representative result is shown. Data are presented as mean ± SD (n ≥ 3). t-test, **P* < 0.05, ***P* < 0.01, ****P* < 0.001. NS, not significant.

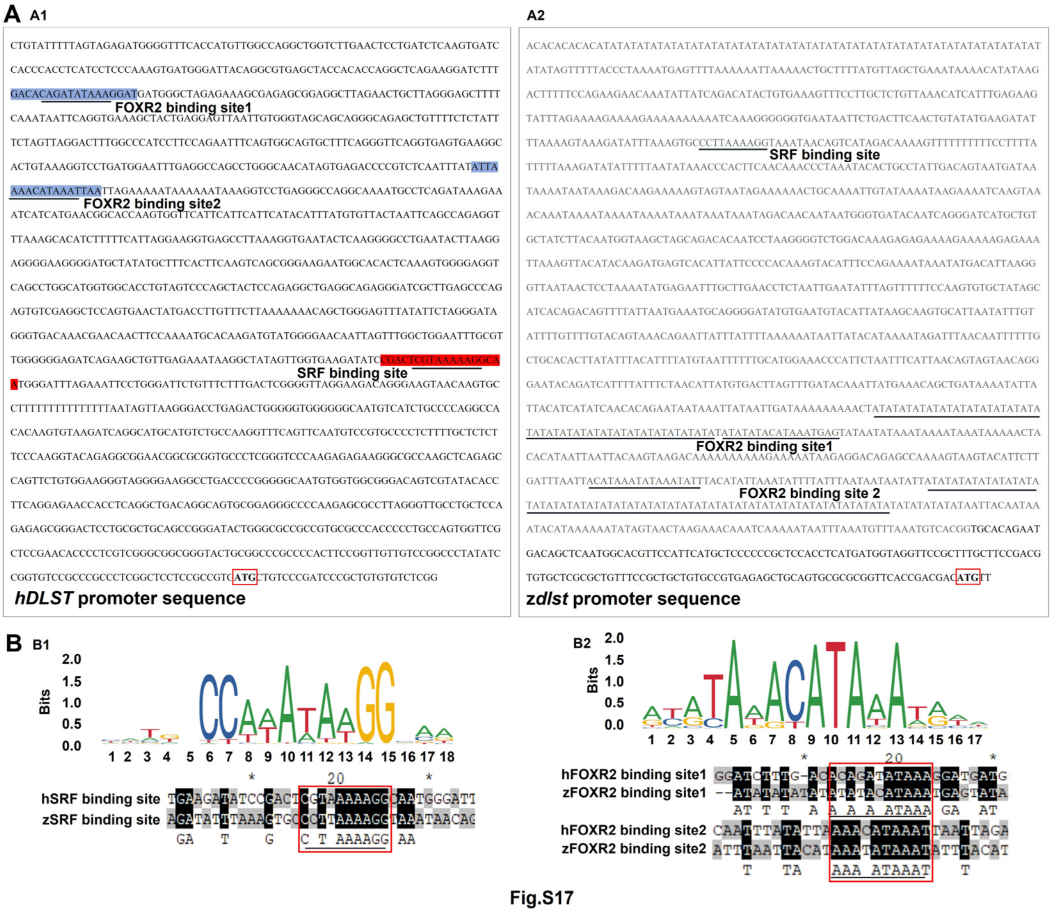

Fig S17. **Detailed information of predicted SRF/FOXR2 binding sites at *hDLST/zdlst* promoter, related to Figure 7.**

(A) Bases of human *DLST* (*hDLST*) promoter sequence (**A1**) and zebrafish *dlst* (z*dlst*) promoter sequence (**A2**), the SRF and FOXR2 binding sites at *hDLST* and z*dlst* promoters are marked with red and blue backgrounds respectively, and the ATG initiation codon are marked with red boxes.

(B) The matrix profiles of SRF/FOXR2 binding sites at *DLST* promoter predicted by JASPAR database. Sequence alignments of SRF/FOXR2 binding sites in human and zebrafish, the conservations of binding sites are marked with red boxes.

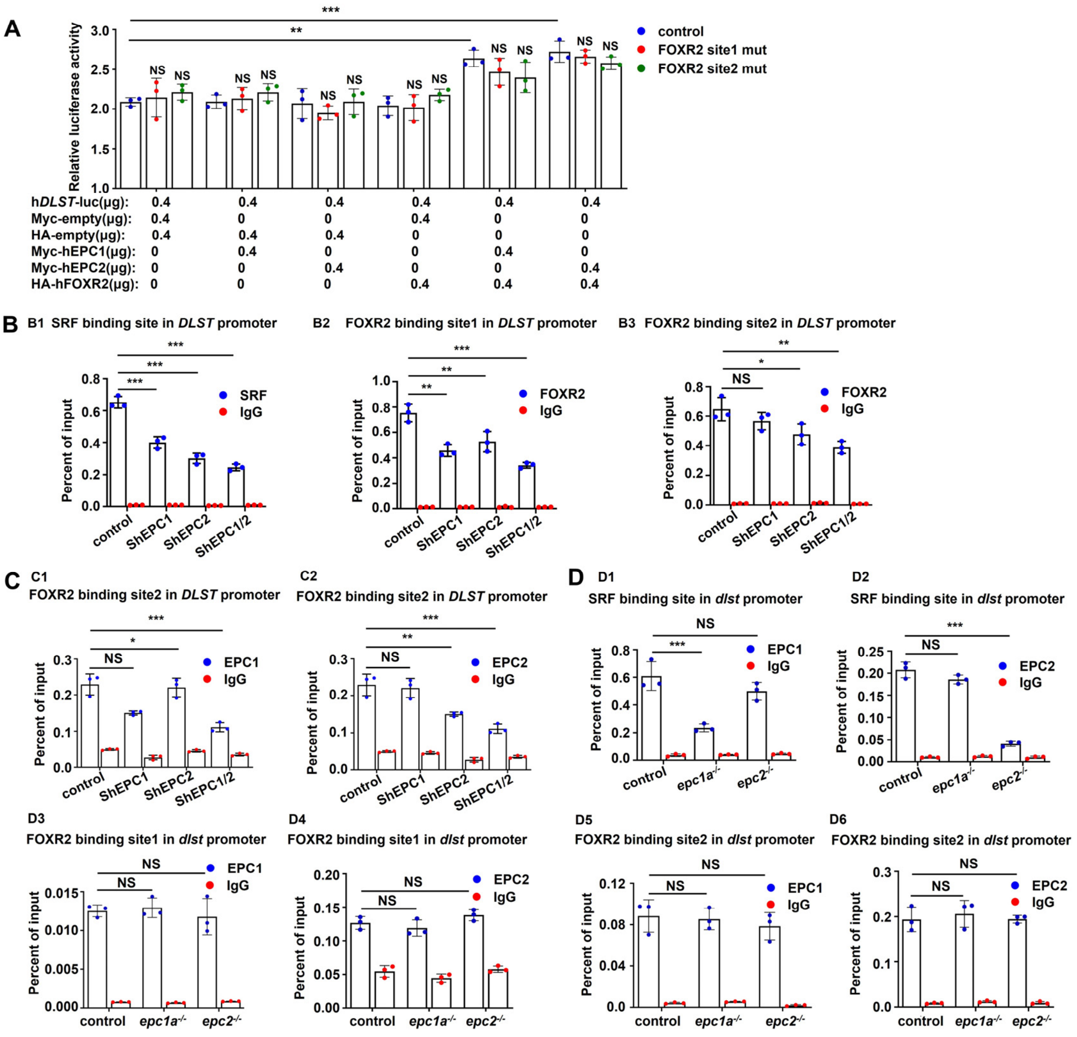

Fig. S18

Fig S18. Luciferase reporter assays of *DLST* promoter with mutated FOXR2 binding site and ChIP-qPCR analysis of EPC1/EPC2 and SRF/FOXR2 occupancies at the predicted FOXR2/SRF binding site in *dlst/DLST* promoter in the control, *epc1a*$^{-/-}$, and *epc2*$^{-/-}$ embryos at 33 hpf, and the control, ShEPC1, ShEPC2 and ShEPC1/2 K562 cells, related to Figure 7.

(**A**) Luciferase reporter assay of the interaction between EPC1/EPC2 and FOXR2 upon *DLST* promoter with mutations of predicted FOXR2 binding site1 or predicted FOXR2 binding site2 in 293T cells.

(**B**) ChIP-qPCR analysis of SRF and FOXR2 occupancies at the predicted SRF and FOXR2 binding sites in *DLST* promoter in the control, ShEPC1, ShEPC2 and ShEPC1/2 K562 cells.

(**C**) ChIP-qPCR analysis of EPC1 and EPC2 occupancies at the predicted FOXR2 binding site1 in *DLST* promoter in the control, ShEPC1, ShEPC2 and ShEPC1/2 K562 cells.

(**D**) ChIP-qPCR analysis of EPC1 and EPC2 occupancies at the predicted SRF binding site (**D1, D2**), FOXR2 binding site1 (**D3, D4**) and FOXR2 binding site2 (**D5, D6**) in *dlst* promoter in the control, *epc1a*$^{-/-}$ and *epc2*$^{-/-}$ embryos at 33 hpf. The occupancy was presented as the percentage of input.

Each experiment was repeated three times, and a representative result is shown. Data are presented as mean ± SD ($n \geq 3$). t-test, **P* < 0.05, ***P* < 0.01, ****P* < 0.001. NS, not significant.

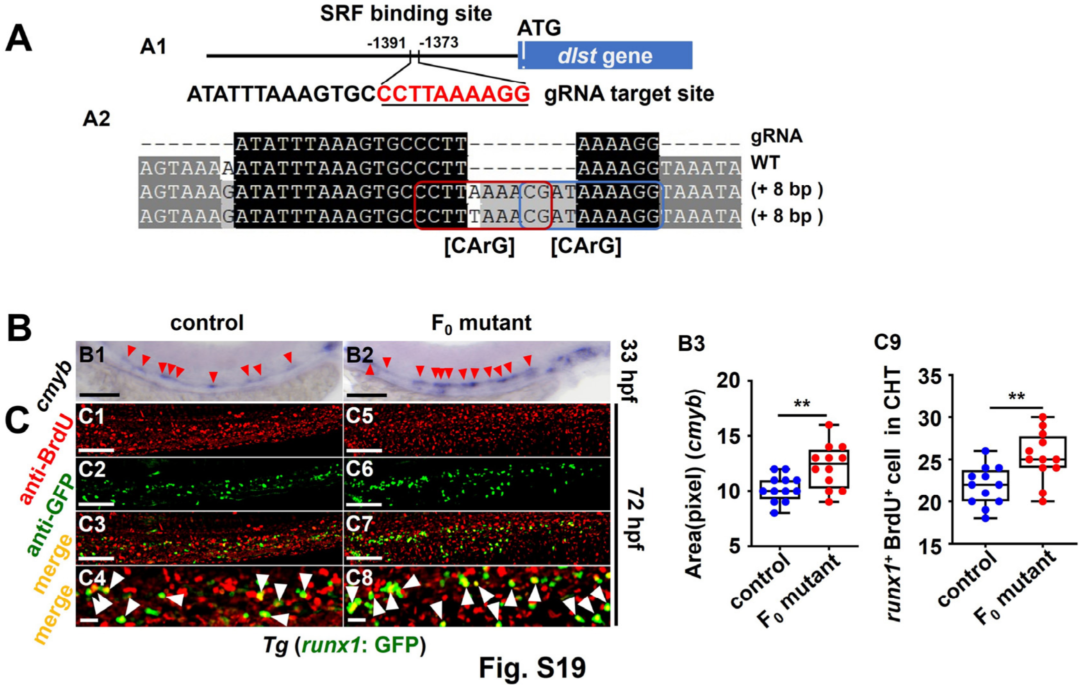

Fig. S19

Fig S19 Genome editing of SRF binding site at zebrafish *dlst* promoter, related to Figure 7.

(**A**) Schematic diagram of gRNA target site at the predicted SRF binding site (dark underline) of zebrafish *dlst* promoter (**A1**), and sequencing of the cloned PCR products that correspond to the mutation site of F_0 embryos (**A2**), and red and blue boxes indicate two SRF motifs (CArG).

(**B**) WISH analysis of *cmyb* in the AGM region in the control and F_0 embryos at 33 hpf, and quantification of the WISH data (**B3**), with red arrowheads indicating positive signals.

(C) Double staining of *runx1*$^{+}$ and BrdU in the CHT region in the control and F_0 mutants at 72 hpf, and quantification of *runx1*$^{+}$BrdU^{+} cells (**C9**), with white arrowheads indicating double-positive cells. **C4** and **C8** show the magnified images for **C3** and **C7**, respectively.

Each experiment was repeated three times, and a representative result is shown. All embryos are shown in lateral view, anterior to the left, and dorsal to the up. Scale bars, 100 µm. Data are presented as mean ± SD (n ≥ 3). t-test, *P < 0.05, **P < 0.01, ***P < 0.001, NS, not significant.

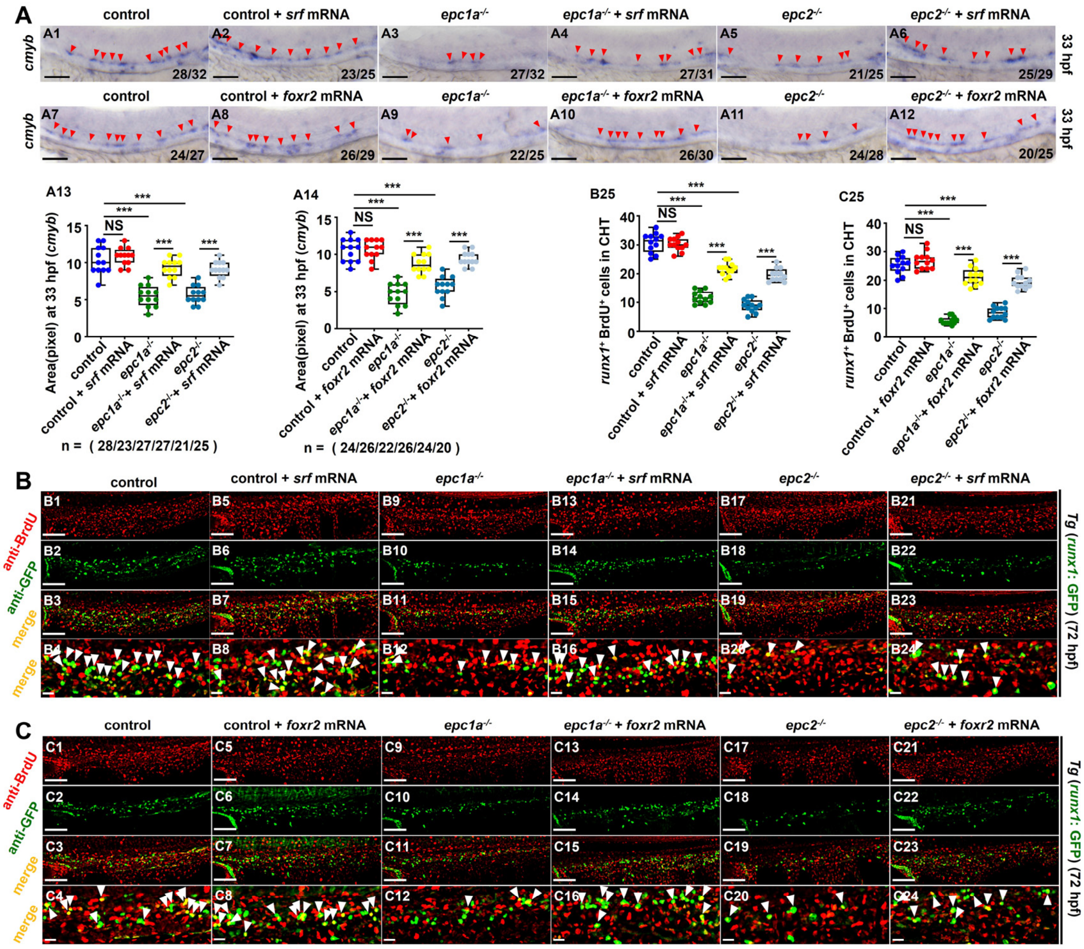

Fig. S20

Fig S20 Ectopic expression of *dlst* and *foxr2* mRNA partially restored the decreases of *cmyb/runx1* and *runx1*$^{+}$BrdU^{+} cells in *epc1a*$^{-/-}$ and *epc2*$^{-/-}$ mutants, related to Figure 7.

(A) WISH analysis of *cmyb* in the AGM region in the control, *epc1a*$^{-/-}$, *epc2*$^{-/-}$ and the corresponding groups injected with *srf* mRNA (**A1-A6**) and *foxr2* mRNA (**A7-A12**) at 33 hpf, and quantification of the WISH data (**A13, A14**), with red arrowheads indicating positive signals.

(B, C) Double staining of *runx1*$^{+}$ and BrdU in the CHT region in the control, *epc1a*$^{-/-}$ and *epc2*$^{-/-}$ embryos and the corresponding groups injected with *srf* mRNA (**B1-B24**) and *foxr2* mRNA (**C1-C24**) at 72 hpf, and quantification of *runx1*$^{+}$BrdU^{+} cells (**B25**, **C25**), with white arrowheads indicating double-positive cells. **B4**, **B8**, **B12**, **B16**, **B20**, **B24**, **C4**, **C8**, **C12**, **C16**, **C20** and **C24** show the magnified images for **B3**, **B7**, **B11**, **B15**, **B19**, **B23**, **C3**, **C7**, **C11**, **C15**, **C19**, and **C23**, respectively.

Each experiment was repeated three times, and a representative result is shown. All embryos are shown in lateral view, anterior to the left, and dorsal to the up. Scale bars, 100 µm. Data are presented as mean ± SD ($n \geq 3$). t-test, *$P < 0.05$, **$P < 0.01$, ***$P < 0.001$, NS, not significant.

Supplemental table 1-19

Table S1 Full names for genes tested in this study, related to all Figures

Gene	Full names of gene
adnpb	activity-dependent neuroprotector homeobox b
atpif1b	ATP synthase inhibitory factor subunit 1b
atm	ATM serine/threonine kinase
brpf1	bromodomain and PHD finger containing, 1
cbx6b	chromobox homolog 6b
cbx8b	chromobox homolog 8b
ccna1	cyclin A1
ccna2	cyclin A2
ccnb1	cyclin B1
ccnd1	cyclin D1
ccng2	cyclin G2
cdc25b	cell division cycle 25B
cdyl	chromodomain protein, Y-like
cenpf	centromere protein F
clocka	clock circadian regulator a
cmyb	v-myb avian myeloblastosis viral oncogene homolog
crebbpa	CREB binding protein a
dlst	dihydrolipoamide S-succinyltransferase
runx1	RUNX family transcription factor 1
myod	myogenic differentiation 1
foxr1 (*foxr2*)	forkhead box R1
srfa	serum response factor a
elp3/kat9	elongator acetyltransferase complex subunit 3
epc1a	enhancer of polycomb homolog 1 (Drosophila) a
epc1b	enhancer of polycomb homolog 1 (Drosophila) b
epc2	enhancer of polycomb homolog 2 (Drosophila)
ep300a	E1A binding protein p300 a
flvcr1	FLVCR heme transporter 1
gata5	GATA binding protein 5
gfi1aa	growth factor independent 1A transcription repressor a
gfi1b	growth factor independent 1B transcription repressor
gtf3c4 (*kat12*)	general transcription factor IIIC, polypeptide 4
haus3	HAUS augmin-like complex, subunit 3
hdac1	histone deacetylase 1
hspa9	heat shock protein 9
idh1	isocitrate dehydrogenase 1 (NADP+)
kat2b	K(lysine) acetyltransferase 2B
kat6a	K(lysine) acetyltransferase 6A
kat7b	K(lysine) acetyltransferase 7b
kat8	K(lysine) acetyltransferase 8
kdm2ba	lysine (K)-specific demethylase 2Ba
klf3	Kruppel-like factor 3
lsm8	LSM8 homolog, U6 small nuclear RNA associated
meox1	mesenchyme homeobox 1
mettl8	methyltransferase 8, methylcytidine
ncoa1	nuclear receptor coactivator 1
ncoa2	nuclear receptor coactivator 2
ncor1	nuclear receptor corepressor 1
oga	O-GlcNAcase
olig2	oligodendrocyte lineage transcription factor 2
pax2a	paired box 2a
poln	polymerase (DNA directed) nu
ptena	phosphatase and tensin homolog A

rag1	recombination activating 1
sfxn4	sideroflexin 4
six3b	SIX homeobox 3b
taf1	TAF1 RNA polymerase II, TATA box binding protein (TBP)-associated factor
wasla	WASP like actin nucleation promoting factor a
BRPF1	bromodomain and PHD finger containing 1
CDYL	chromodomain Y like
DLST	dihydrolipoamide S-succinyltransferase
EPC1	enhancer of polycomb homolog 1
EPC2	enhancer of polycomb homolog 2
FOXR2	forkhead box R2
GFI1B	growth factor independent 1B transcriptional repressor
KAT2A (*GCN5*)	lysine acetyltransferase 2A
KAT7 (*HBO1/MYST2*)	lysine acetyltransferase 7
KAT8 (*MYST1*)	lysine acetyltransferase 8
KAT9 (*ELP3*)	elongator acetyltransferase complex subunit 3
KAT12 (*GTF3C4*)	general transcription factor IIIC subunit 4
KAT13A (*NCOA1*)	nuclear receptor coactivator 1
SRF	serum response factor

Table S2 Sequences of zebrafish for gRNA, related to Figures 1 and 4

Gene	Primer sequence(5'to3')
epc1a -gRNA	5' GTAATACGACTCACTATAGGTCTGGCAGATCCTCGCAGGTTTTAGAGCTAGAAATAGC 3'
epc2 -gRNA	5' GTAATACGACTCACTATAGGGCGTCTAGCGCTCGCGCGGTTTTAGAGCTAGAAATAGC 3'
dlst -gRNA	5' GTAATACGACTCACTATAGGGTATGGGTCCACCCGCAGGTTTTAGAGCTAGAAATAGC 3'

Table S3 Primers sequences used for screening homozygous mutants, related to Figures 1 and 4

Gene	Primer sequence (5'to3')
epc1a -det-F	5' TTTCTCATTCTCCACCTCC 3'
epc1a -det-R	5' ACAAAATAAATCCCAGCA 3'
epc2 -det-F	5' TCAGAGATGCCTTTAACCGTC 3'
epc2 -det-R	5' ACCTCCGTGAGCAAAGAATT 3'
dlst -det-F	5' GTAAACGTGAGCTTGTGC 3'
dlst -det-R	5' TCAACCTCATCTCTACTGC 3'

Table S4 Sequences of human for ShRNA, related to Figure 5

Gene	Primer sequence (5'to3')
EPC1	F1: 5' CCGGGGGCCATCTCTTATTCCATCACTCGAGTGATGGAATAAGAGATGGCCCTTTTTG 3'
	R1: 5' AATTCAAAAAGGGCCATCTCTTATTCCATCACTCGAGTGATGGAATAAGAGATGGCCC 3'
	F2: 5' CCGGGGCAGGTTCAGCTTTGTTACACTCGAGTGTAACAAAGCTGAACCTGCCTTTTTG 3'
	R2: 5' AATTCAAAAAGGCAGGTTCAGCTTTGTTACACTCGAGTGTAACAAAGCTGAACCTGCC 3'
	F3: 5' CCGGGCCCAAAGTCTTACCATCGTCCTCGAGGACGATGGTAAGACTTTGGGCTTTTTG 3'
	R3: 5' AATTCAAAAAGCCCAAAGTCTTACCATCGTCCTCGAGGACGATGGTAAGACTTTGGGC 3'
	F4: 5' CCGGGGATGCTGAACAGCCTGATTACTCGAGTAATCAGGCTGTTCAGCATCCTTTTTG 3'
	R4: 5' AATTCAAAAAGGATGCTGAACAGCCTGATTACTCGAGTAATCAGGCTGTTCAGCATCC 3'
EPC2	F1: 5' CCGGGGACCAATGCTTCCAGTAAACCTCGAGGTTTACTGGAAGCATTGGTCCTTTTTG 3'
	R1: 5' AATTCAAAAAGGACCAATGCTTCCAGTAAACCTCGAGGTTTACTGGAAGCATTGGTCC 3'
	F2: 5' CCGGGCAACCCACTCCTGAGACATTCTCGAGAATGTCTCAGGAGTGGGTTGC TTTTTG 3'
	R2: 5' AATTCAAAAAGCAACCCACTCCTGAGACATTCTCGAGAATGTCTCAGGAGTGGGTTGC 3'
	F3: 5' CCGGTGGACCGAATATCCACAGAACCTCGAGGTTCTGTGGATATTCGGTCCATTTTTG 3'
	R3: 5' AATTCAAAAATGGACCGAATATCCACAGAACCTCGAGGTTCTGTGGATATTCGGTCCA 3'

Table S5 Sequences of zebrafish for Morpholinos, related to Figures 1 and 4

Gene	Primer sequence (5'to3')
epc1a- MO	5' CTGAACGACAGTTTGCTCATTTTAC 3'
epc2 -MO	5' CGCGGAACGAGAGTTTACTCATTGT 3'
dlst -MO	5' ATGATCCATTTTCACTCCTCAGCCACAC 3'

Table S6 Sequences of primers for mRNA synthesis, related to Figures 1, 4 and 7

Gene	Primer sequence (5' to 3')
epc1a -ORF-F	5' TAATACGACTCACTATAGGG AGCAGGTGTACGGAGAGAAG 3'
epc1a- ORF-R	5' GGACTGGAAACGGTCACTAC 3'
epc2 -ORF-F	5' TAATACGACTCACTATAGGGATGAGTAAACTCTCGTTCCG 3'
epc2- ORF-R	5' GGATGGGCTATGTGACCTCC 3'
dlst -ORF-F	5' TAATACGACTCACTATAGGG ATGTTGTGTCATTCCCGC 3'
dlst- ORF-R	5' GATTGGTGGAGCCGCATT 3'
foxr2 -ORF-F	5' TAATACGACTCACTATAGGG AGCGAACGTTTTGCTGAATGT 3'
foxr2- ORF-R	5' ATTGGTGGATCACAGCTCTAACAA 3'
srf -ORF-F	5' TAATACGACTCACTATAGGG TGCAGGTCAGCGATGTTAGG 3'
srf- ORF-R	5' TGGCAGTCTGATTGGATGGC 3'

Table S7 Zebrafish primer sequences used for qRT-PCR, related to Figures 1, 3, 4 and 7

Gene	Primer sequence (5'to3')	GenBank ID
cmyb	F: 5' AAGAAAGCGCACATGGCAAC 3'<br>R: 5' AGCAAAGAAAAGTGGGGGCA 3'	NM_131266.2
runx1	F: 5' CCGCCATCAAGAACCAAGTG 3'<br>R: 5' ATGGCACTTCGCCTCAACTG 3'	XM_021475829.1
myod	F: 5' TATCCCCTTCCCCATCCC 3'<br>R: 5' CCTTGCTCAGTCGCCTCC 3'	NM_131262.2
dlst	F: 5' GTTTTCGGCTCCATGTTCGG 3'<br>R: 5' AATCAAGCGGTGGTCGTAGG 3'	NM_201487.1
epc1a	F: 5' AAGACCTGGGCAACGGCTAT 3'<br>R: 5' CTACTGCAGGTGGTCCCATT 3'	XM_005156406.4
epc1b	F: 5' TTCCTCATCCGCCCAATCC 3'<br>R: 5' CTGATACTGCTCTGCGGTGAA 3'	NM_001089409.1
epc2	F: 5' GCTCCCATATCCCTACCCAT 3'<br>R: 5' CTGATGCTGGAGACAGTGGA 3'	NM_201075.1
kat2b	F: 5' TACTGGCAGCTAGAAACGCC 3'<br>R: 5' AATCTGTGTGGCCTCGTACC 3'	NM_001045034.1
kat8	F: 5' GTTGGAAAGGCTCGTTTGGC 3'<br>R: 5' GTAGTGGGGTCCATCTCTGC 3'	NM_212742.1
kat7b	F: 5' CGGCGGAAGGGCCATAAATC 3'<br>R: 5' GGGTGTTATTGCGCTGGTTG 3'	NM_212635.1
kat6a	F: 5' CAGTGCGGGTCAAAGCTCTA 3'<br>R: 5' CCTTTCGGCATTCGCGTTAG 3'	NM_001123312.4
ncoa1	F: 5' ATGCCTGGCTCACCTCAATC 3'<br>R: 5' ATTGACCCCGATGGAAAGGC 3'	XM_686652.8
ncoa2	F: 5' TCAGTTCAGCCAACAAGCCA 3'<br>R: 5' TCAGTTCAGCCAACAAGCCA 3'	NM_131777.1
clocka	F: 5' CCGAGTGGTGGTGGATTGAAC 3'<br>R: 5' CTGAGACTGCTGGTTGAGGTTG 3'	NM_130957.2
crebbpa	F: 5' AGGGAACATGGTCACGCAAA3'<br>R: 5'CCAAGTGCATTCAGGGGGAG 3'	XM_005161769.4
ep300a	F: 5' AGCATGACCTTCCAGACGAG 3'<br>R: 5' ATTGAGGCTCCTCCTGGGTT 3'	XM_021480295.1
elp3	F: 5' CAGAGGACACACAGTTCGGG 3'<br>R: 5'GTCCAGTGCCTCGTATCACC 3'	NM_001014322.2
gtf3c4	F: 5' GAATGGAGACGTGGTGGTGT 3'<br>R: 5'GCTCCCTATGATCAGTCCGC 3'	XM_691843.8
taf1	F: 5'GTGACTACCTCAATCGGCCT 3'<br>R: 5'GTGACTACCTCAATCGGCCT 3'	NM_001044785.1
mettl8	F: 5' CCATCAGAGGCAGTAAGGCG 3'<br>R: 5' GGCAGAGAGCACAAAGACCA 3'	NM_001321154.2
oga	F: 5' GGAGATGTGCTGCTCTGTCA 3'<br>R: 5'GGAGATGTGCTGCTCTGTCA 3'	NM_001305556.1
cdyl	F: 5'CATGGGAGTTGCATCGGCTA 3'<br>R: 5' TCCTCACCAGTGCTTTGGAC 3'	XM_691787.8
brpf1	F: 5'TCTGATTGACGAGGATGCGG 3'<br>R: 5' GAGTCGTCGGTCTGCTTGAA 3'	NM_201016.1
kdm2ba	F: 5'CAGTGGGTGGAAGGGCTAAA 3'<br>R: 5'CAGACGGGAGAGTAGAGGCA 3'	NM_213167.1
cbx6b	F: 5' CGTCCGTAAGGGAAGGATGG 3'<br>R: 5'TCGGCTTGTGCTCTTGACTT 3'	XM_021474789.1
cbx8b	F: 5' TCGGCTTGTGCTCTTGACTT 3'<br>R: 5' AGCGTAATTTGGGCTTCCGT 3'	NM_001024415.2
hdac1	F: 5'CTGCTCATGCTGGGAGGTGG 3'<br>R: 5'GTTGAAGGGGCTGATGTGAAGT 3'	NM_004964.3

gfi1b	F: 5' TGTGCATCGACCAAACGACA 3'<br>R: 5'GAGTGATGGGATTGGGCAGT 3'	NM_001271841.1
gfi1aa	F: 5' GCACGGCAGATCAGTCTTCT 3'<br>R: 5' TGTCGAAAGGCTGAGAGTCG 3'	NM_001020776.1
cdc25b	F: 5' TTTTGCCTGCCGACCGAA 3'<br>R: 5' CCCTCCAGAAAGCCATCA 3'	NM_001115095.2
atm	F: 5' TCAGCCTCCACTCAAGCA 3'<br>R: 5' TGAAGAACCTCCACCACA 3'	XM_002664557.3
ccnb1	F: 5' ATGGTCCACTACCCTCCC 3'<br>R: 5' CAGCCAGATGCTTAGAAA 3'	NM_131513.1
ccna1	F: 5' TCGCCACCATTGACCTTC 3'<br>R: 5' GACTTGACCTCCATTTCC 3'	NM_152949.1
ccna2	F: 5' TCGCCACCATTGACCTTC 3'<br>R: 5' GACTTGACCTCCATTTCC 3'	NM_152949.1
ccnd1	F: 5' TGTTGAATGACCGAGTTT 3'<br>R: 5' TCCAGGTAGTTCATAGCC 3'	NM_131025.4
ccng2	F: 5' TTGTCGCTGTTGATGCTT 3'<br>R: 5' CGGTTTGGCACATTCTGG 3'	NM_213172.1
cenpf	F: 5' CAGTCGGTTTCTAAGTGTTC 3'<br>R: 5' CCTTGATTTTCTGCTCCA 3'	XM_021467448.1
srfa	F: 5' CATACAGTGTCCACAGACGCA 3'<br>R: 5' GACTGGGATGAACCTGGATGG 3'	NM_001110526.1
foxr2	F: 5' GTGCAGGACACCTCTGTCTC 3'<br>R: 5' ACCCAGCCTACGAGTGTTTG 3'	NM_001103124.1
18s	F: 5' GCATACCCGTAACCACAAT 3'<br>R: 5' GCAAACAAGAAGCCCAAA 3'	
gapdh	F: 5' TGAGGGTCCCAATTACCA 3'<br>R: 5' AGCAGATGCCCATTTAGC 3'	
β-actin	F: 5' ACCTACAATCTCTACATCGCTGC 3'<br>R: 5' CCCTTTCCTCCCCAAATACC 3'	

Table S8 Human primer sequences used for qRT-PCR, related to Figures 5 and 7

Gene	Primer sequence (5'to3')	GenBank ID
FOXR2	F: 5' GACGACAACTCCCTCCAGTC 3'<br>R: 5' AAAGGGGAAATGCTGTCGGG 3'	NM_198451.4
SRF	F: 5' CGGGTACAACCTCCACCATC 3'<br>R: 5' ACACTGGTGCCAGGTAGTTG 3'	NM_003131.4
DLST	F: 5' TGATGGGGGAAAAGTCGAAGG 3'<br>R: 5' CTCAGTCCCAATCCCAAGAGG 3'	NM_001933.5
EPC1	F: 5' CTGCACGAATACGCCTCGAT 3'<br>R: 5' CCCTCTTCTCGCCATACACC 3'	NM_001272004.3
EPC2	F: 5' GGGGGTATCACAGAAGAGCAG 3'<br>R: 5' TGTCTGTTGCGAGGAATGCT 3'	NM_015630.4
RUNX1	F: 5' GATGGCACTCTGGTCACTGT 3'<br>R: 5' CCACTTCGACCGACAAACCT 3'	NM_001754.5
FLI1	F: 5' ACGTCAAGCGGGAGTATGAC 3'<br>R: 5' TTGGTGGTCATGTTGGGAGG 3'	NM_002017.5
FLK1	F: 5' CAAAGGGTGGAGGTGACTGCG 3'<br>R: 5' ATAGACATAAATGACCGAGGC 3'	NM_002253
GATA1	F: 5' TTCAGGTGTTCCCATTGCTCA 3'<br>R: 5' GCCGCTCTGTCTTCAAAGTCTCC 3'	NM_002049.4
GATA2	F: 5' TACCACAAGATGAATGGGCAGAA 3'<br>R: 5' ACAGGGTCCCCGTTGGCGTTT 3'	NM_032638.5
CMYB	F: 5' TGTTTGCAACTGGGGAGACA 3'<br>R: 5' TCCTGGCTTCTTCACACGAA 3'	NM_001130173.2
CD41	F: 5' GATGGCGTCGTATTTTGGGC 3'<br>R: 5' CACTCTGACCCAGGAACACC 3'	BC126442.1
CD45	F: 5' TGCAAACATCACTGTGGATTACT 3'<br>R: 5' GCACCTCATTGTTTGTGCAAG 3'	NM_002838.5
CDH5	F: 5' ATTTTCCAGCAGCCTTTCTAC 3'<br>R: 5' CGTGTTATCGTGATTTTCCGT 3'	NM_001795.5
PSME4	F: 5' CCATGCTGGGGTGCTAGG 3'<br>R: 5' TGCCAGTTGTCATGGTGAGT 3'	NM_014614.3
LDB1	F: 5' AGAACACCCAGTTTGACGCA 3'<br>R: 5' GACGTGGGGTTCTCCGATTT 3'	NM_001113407.3
ADAM8	F: 5' CTGACATGTGTGGCGTTCTG 3'<br>R: 5' GCTTGTGGTTGCACACCTTC3'	NM_001109.5
CSF1	F: 5' TGCCAGCGGAACTTGTAGAT 3'<br>R: 5' GGAAACTCGGTGTCCATAGCA 3'	NM_000757.6
NOTCH1	F: 5' TGAATGGCGGGAAGTGTGAA 3'<br>R: 5' CACAGCTGCAGGCATAGTCT3'	NM_017617.5
JUN	F: 5' TAATCCAGTCCAGCAACGGG 3'<br>R: 5' GTGTTCTGGCTGTGCAGTTC 3'	NM_002228.4
ZMYND8	F: 5' CAGGTCGATGCCCGATTCTT 3'<br>R: 5' GCTGTTGTGTGTGTAGGGTG 3'	NM_001281769.2
BRD8	F: 5' AGTACCCCAGTCACCTCGTT 3'<br>R: 5' GGGGTGGAGTTGCTTTCTGT3'	NM_001164326.2
PHIP	F: 5' CCGTGGATCTGCAAGCCTAT 3'<br>R: 5' GGGAAGAAACCCGCCTGTAA 3'	NM_017934.7
BRCA2	F: 5' TGGTGGATGGCTCATACCCT 3'<br>R: 5' AGCACCCTTTCTGGGCTTAG 3'	NM_000059.4
MLLT3	F: 5' AGAATGGTGAATGTGACAAGGC 3'<br>R: 5' TGGTTTTGTCCAGCGAGCAA 3'	NM_001286691.2

EID1	F: 5' GGGAAACGGAGCCTTGCTAA 3'<br>R: 5' AGCTGCTCCTCTTCGGGATA3'	NM_014335.3
JADE1	F: 5' TGGCCAGGCCCAAGAAGTA 3'<br>R: 5' GCATCGCTGCTCAAATTCCT 3'	NM_001287437.2
MECP2	F: 5' TCGAAAAGGTAGGCGACACA 3'<br>R: 5' TTTCACCTGCACACCCTCTG 3'	NM_001110792.2
LIF	F: 5' ATGAAGGTCTTGGCGGCAG 3'<br>R: 5' TTGTGACATGGGTGGCGTAT 3'	NM_001257135.2
hGAPDH	F: 5' GTCTCCTCTGACTTCAACAGCG 3'<br>R: 5' ACCACCCTGTTGCTGTAGCCAA 3'	
hβ-ACTIN	F: 5' AGCGAGCATCCCCCAAAGTT 3'<br>R: 5' GGGCACGAAGGCTCATCATT 3'	

Table S9 Sequences of primer used for amplifying probes for WISH, related to Figures 1 and 4

Gene	Primer sequence (5'to3')	GenBank ID
haus3	F: 5' GACACAATCTCACTTGACTG 3'<br>R: 5' GCTAATACGACTCACTATAGGAGTGCAGGTGTGGCTGTA 3'	NM_001077171.2
meox1	F: 5' GCAGTGGGAGCTGAAATGGA 3'<br>R: 5' GCTAATACGACTCACTATAGGGGTTGACTGCGATCTCGTA 3'	NM_001002450.2
adnpb	F: 5' CCGTAGACCCGCACAGGCAC 3'<br>R: 5' GCTAATACGACTCACTATAGGTCTTCTACCTGTCCCGCGCT 3'	NM_001347570.2
lsm8	F: 5' AGCGAACGAGCACCGGAGGA 3'<br>R: 5' GCTAATACGACTCACTATAGGAGGATGAGGTTGATTGCCTG 3'	NM_001302466.1
hspa9	F: 5' ACTGCTTCTACATCGCACT 3'<br>R: 5' GCTAATACGACTCACTATAGGACTAACCTTGCCGTTCTCG 3'	NM_201326.2
ptena	F: 5' TCTTCATCCGGGTCAGTGCT 3'<br>R: 5' GCTAATACGACTCACTATAGGCTGGTTCCCTGCGCTTTCA 3'	NM_200708.2
ncor1	F: 5' TTGTATGGATGAATGGATGG 3'<br>R: 5' GCTAATACGACTCACTATAGGTGTGGTTCCTCCCTACAGT 3'	XM_009301630
flvcr1	F: 5' TTCTCCTTGGGATTGTGCTG 3'<br>R: 5' GCTAATACGACTCACTATAGGATGGTGGTCTCGTGAGGT 3'	XM_005160739.3
idh1	F: 5' GGTTATGGATCATTGGGCAT 3'<br>R: 5' GCTAATACGACTCACTATAGGTGCGTTCTTATCCAGCTCCG 3'	NM_201499.1
wasla	F: 5' AGAAGCGCACTCAACGAACT 3'<br>R: 5' GCTAATACGACTCACTATAGGCCTACATGACCGATGTGCT 3'	NM_001083006.1
atpif1b	F: 5' CAGGCTGGAGGTGCATTTGG 3'<br>R: 5' GCTAATACGACTCACTATAGGTGGCGGGTGATCTCATGCT 3'	NM_001044859.1
poln	F: 5' GCCTTCATCCAGCACACCAT 3'<br>R: 5' GCTAATACGACTCACTATAGGGTCTTCTTCACCAACGCAGC 3'	NM_001100026.1
sfxn4	F: 5' AACATATTGGTGGTGAGAAG 3'<br>R: 5' GCTAATACGACTCACTATAGGCAGTACACATGCTCCCAATT 3'	NM_001076662.1
kat8	F: 5' GACTCTGGTCACGCTGGAAC 3'<br>R: 5' GCTAATACGACTCACTATAGGCCAAGCGACACTCCGTCA 3'	NM_212742.1
klf3	F: 5' ACCCTTCGTATCGAGTCCTG 3'<br>R: 5' GCTAATACGACTCACTATAGGATGACCGATTTCTCTTGGCT 3'	NM_131859.3
dlst	F: 5' TACGCTCTAACAGACCAGC 3'<br>R: 5' GCTAATACGACTCACTATAGGCTGTGGCGGGTTGATGATTG 3'	NM_201487.1
epc1a	F: 5' GGCAGCGGAACTGTCTCA 3'<br>R: 5' GCTAATACGACTCACTATAGGGGCTTGTAGGAGAGTGGGCA 3'	NM_001089409.1
epc2	F: 5' GCTCCCATATCCCTACCCAT 3'<br>R: 5' GCTAATACGACTCACTATACTGATGCTGGAGACAGTGGA 3'	NM_201075.1

Table S10 Sequences of primer used for luciferase reporter assay and CoIP, related to Figures 6 and 7

Genes	Primer sequence (5'to3')
pGL3-*hDLST* -promoter-xhoI-F	5' cctcgagCTCCCTTGCTTTATCCGACCA 3'
pGL3-*hDLST* -promoter-HindIII-R	5' cccaagcttTTCCCCTACCCTTCCACAGAA 3'
hEPC1 -myc-EcoRI-F	5' ggaattcCTGCGCCTGAGATGAGTAAACTG 3'
hEPC1 -myc-XhoI-R	5' cctcgagACCTAATCAAGTCCCCAGGCTG 3'
hEPC2- myc-EcoRI-F	5' ggaattcGGCGGGGAGACAATGAGTAAA 3'
hEPC2 -myc-XhoI-R	5' cctcgagATGACTGCACACCATCAGCACA 3'
hFOXR2 -HAM-XbaI-F	5' ctctagaGTCTCTCTCCACCTATCTCTCCAGTAA 3'
hFOXR2 -HAM-BamHI-R	5' cggatccGCACAGTAAGGAAGGCAGTGTTC 3'
hSRF- HAM-XbaI-F	5' ctctagaACCTTTGCCACCCGAAAACT 3'
hSRF -HAM--BamHI-R	5' cggatccCCATCCCTTGGGCCATCTGT 3'
FOXR2-A-Xho-frag1-418bp-F	5' gcgtgctagcccgggctcgagCTCCCTTGCTTTATCCGACCA 3'
FOXR2-A-frag1-418bp-R	5' ctagcccatcCAAAGATCCTTCTGAGCCTGGT 3'
FOXR2-A-frag2-1447bp-F	5' aggatctttgGATGGGCTAGAGAAAGCGAGAG 3'
FOXR2-A-HindIII-frag2-1447bp-R	5' cagtaccggaatgccaagcttATCGGGACAGCATCACGGC 3'
FOXR2-B-xho-frag1-697bp-F1	5' gcgtgctagcccgggctcgagCTCCCTTGCTTTATCCGACCA 3'
FOXR2-B-frag1-697bp-R1	5' ATAAATTGAGACGGGGTCTCACTATG 3'
FOXR2-B-frag2-1168bp-F1	5' gagaccccgtctcaatttatTTAGAAAAATAAAAAATAAAGGTCCTGAG 3'
FOXR2-B-HindIII-frag2-1168bp-R1	5' cagtaccggaatgccaagcttATCGGGACAGCATCACGGC 3'
SRF-xho-frag1-1250bp-F	5' gcgtgctagcccgggctcgagCTCCCTTGCTTTATCCGACCA 3'
SRF-frag1-1250bp-R	5' tcccaGATATCTTCACCAACTATAGCCTTATTTC 3'
SRF-frag2-614bp-F	5' gttggtgaagatatcTGGGATTTAGAAATTCCTGGGA 3'
SRF-HindIII-frag2-614bp-R	5' cagtaccggaatgccaagcttATCGGGACAGCATCACGGC 3'
hKAT2A/GCN5 -HAM-XbaI-F	5' ctctagaTTGCCCATGCGGCCCTA 3'
hKAT2A/GCN5 -HAM-Not-R	5' ttgcggccgcGTCTCAAGCTGAGTCGGGTC 3'
hKAT6A -HAM-XbaI-F	5'ctctagaGGGTGGCATTCTGTTTTGTG 3'
hKAT6A -HAM-Not-R	5' ttgcggccgcAAGGTCCATTTTTCTCTGGTTTG 3'
hKAT7- HAM-XbaI-F	5'ctctagaCGAATCGGAACCGTCGGG 3'
hKAT7 -HAM-NotI-R	5' ttgcggccgcCGGATTCCTACTGCTGGGGT 3'
hKAT8/MYST1 -HAM-XbaI-F	5' ctctagaACTTCCCTTCCCGCGAT 3'
hKAT8/MYST1 -HAM-BamHI-R	5' cggatccAGCTGGTCTGACTGGTTTGG 3'
hKAT9/ELP3 -HAM-XbaI-F	5' ctctagaCCTCCTCAGTATCGCAAGGTC 3'
hKAT9/ELP3- HAM-NotI-R	5' ttgcggccgcGCTCAGCCTCTCTGTTGAGT 3'
hKAT12/GTF3C4 -HAM-XbaI-F	5' ctctagaTCCGACCTCTGCACCTGA 3'
hKAT12/GTF3C4 -HAM-NotI-R	5' ttgcggccgcATCATGCCCTTCCATCTTCCC 3'
hKAT13A/NCOA1 -HAM-XbaI-F	5' ctctagaAAGCTACCCTCTGGAACTCAA 3'
hKAT13A/NCOA1 -HAM-NotI-R	5' ttgcggccgcAACGACCTGAAGAATGGCTG 3'
hBRPF1 -HAM-XbaI-F	5' ctctagaTGTGACAGCATGGGGGTG 3'
hBRPF1 -HAM-NotI-R	5' ttgcggccgcTGAGCAAAGGGGAGGAGAGA 3'
hCDYL -HAM-XbaI-F	5' ctctagaCGCAGGACCCAACTGAAACA 3'
hCDYL -HAM-BamHI-R	5' cggatccCGGTGATGTTCTCCTGCTCAG 3'
hGFI1B -HAM-XbaI-F	5' ctctagaCGAGAGAGGCTTTGCAGTT 3'
hGFI1B- HAM-BamHI-R	5' cggatccGGAGACTGGAGATTTGGGCA 3'

Table S11 Sequences of primer used for ChIP-qPCR, related to Figures 3 and 7

Genes	Primer sequence (5'to3')
cmyb -ChIP-115bp-F	5' CTGGAACCACAAGCCAGTTTTT 3'
cmyb -ChIP-115bp-R	5' AAACAGAGCGTGAGGGGGTC 3'
runx1 -ChIP-166bp-F	5' AGCGCATCATCTGATCCAGG 3'
runx1 -ChIP-166bp-R	5' GAAATCATCATCGGCCACGC 3'
dlst- ChIP-100bp-F	5' GTCACGGTGCACAGAATGACAG 3'
dlst- ChIP-100bp-R	5' GAGCACACGTCGGAAGCA 3'
dlst/srf -ChIP-161bp-F	5' GGGGGGTGAATAATTCTG 3'
dlst/srf- ChIP-161bp-R	5' AGGGTTTGTTGAAGTGGG 3'
dlst/foxr2 -1-ChIP-384bp-F	5' CTGGCTGCAAATGTGTATTC 3'
dlst/foxr2 -1-ChIP-384bp-R	5' CTTTCACAGTATGTCTG 3'
dlst/foxr2 -2-ChIP-297bp-F	5' GTAACAGGGAATACAGATCA 3'
dlst/foxr2 -2-ChIP-297bp-R	5' CTTACTTTTGGCTCTGTCC 3'
dlst/foxr2 -3-ChIP-271bp-F	5' GGACAGAGCCAAAAGTAAG 3'
dlst/foxr2 -3-ChIP-271bp-R	5' CGTGCCATTGAGCTGTCA 3'
DLST -ChIP-173bp-F	5' TCAGTTCAATGTCCGTGCCC 3'
DLST -ChIP-173bp-R	5' GTGTATACGACTGTCCCGCC 3'
DLST/SRF -ChIP-154bp-F	5' TTAGTTTGGCTGGAATTTGCGT 3'
DLST/SRF -ChIP-154bp-R	5' ACTTCCCTGTCTTCCTAACCC 3'
DLST/FOXR2 -1-ChIP-187bp-F	5' TGATGGGATTACAGGCGTGA 3'
DLST/FOXR2 -1-ChIP-187bp-R	5' AGAGAAAACAGCTCTGCCCTG 3'
DLST/FOXR2 -2-ChIP-142bp-F	5' AGGCACTGTAAAGGTCCTGAT 3'
DLST/FOXR2 -2-ChIP-142bp-R	5' TTTATCTGAGGCATTTTGCCTGG 3'

Table S12 Hematopoiesis and cell cycle-associated DEGs in epc1-/- embryos at 28 hpf, related to Figures 2 and 3

Up-regulated DEGs related to hematopoiesis

gene ID	control_1 FPKM	control_2 FPKM	epc1_mut_1 FPKM	epc1_mut_2 FPKM	Qvalue (control-vs-epc1_mut)	Pvalue (control-vs-epc1_mut)	log2 (epc1_mut/control)	Other Gene ID
321217	19.61	22.13	29.73	34.08	1.65E-71	1.18E-72	0.705806119	*mbd3b*
566857	4.12	4.73	8.29	9.93	6.01E-46	6.14E-47	1.020940217	*poln*
373085	35.49	38.16	73.25	79.08	0.00E+00	0.00E+00	1.051026441	*hspa9*
436723	9.17	8.21	17.94	19.87	7.81E-54	7.05E-55	1.128837813	*meox1*
562595	1.49	1.8	4.23	4.23	1.14E-43	1.23E-44	1.36390511	*flvcr1*
556121	1.8	1.65	4.08	5.26	6.97E-20	1.42E-20	1.442590598	*sfxn4*
415205	0.39	0.1	0.52	0.87	6.32E-05	3.82E-05	1.502382272	*eaf1*
393246	1.14	1.25	5.38	4.54	1.01E-275	1.91E-277	2.089531304	*ncor1*
553814	0.05	0.15	0.99	0.81	2.11E-13	5.89E-14	3.138803821	*gfi1aa*
368262	3.9	2.73	30.59	29.29	0.00E+00	0.00E+00	3.178444057	*dlst*
566017	9.66	5.84	69.62	73.32	0.00E+00	0.00E+00	3.219174468	*lsm8*
393665	0.07	0.02	0.58	0.56	1.16E-11	3.61E-12	3.716977156	*ndrg1b*
504174	0	0	0.2	0.8	1.15E-04	7.28E-05	4.893059017	*ba1l*
558368	0	0	0	3.58	4.22E-13	1.19854E-13	6.695034603	*atpif1b*

Down-regulated DEGs related to hematopoiesis

gene ID	control_1 FPKM	control_2 FPKM	epc1_mut_1 FPKM	epc1_mut_2 FPKM	Qvalue (control-vs-epc1_mut)	Pvalue (control-vs-epc1_mut)	log2 (epc1_mut/control)	Other Gene ID
794088	3.2	0.46	0	0	2.04E-59	1.71662E-60	-9.362942571	*ptena*
334316	0.81	0.85	0	0	2.88E-29	4.28285E-30	-8.098300056	*brf1b*
110439183	11.78	15.94	2.78	1.15	1.32E-239	2.9747E-241	-2.818052382	*idh1*
100009637	1.33	1.26	0.2	0.19	2.40E-32	3.28638E-33	-2.696436228	*wasla*
117603	3.49	4.13	0.74	0.63	3.14E-92	1.79521E-93	-2.450199543	*klf3*
100003790	3.4	3.62	1.29	1.01	2.88E-25	4.8312E-26	-1.602501779	*kat8*
566923	7.3	6.28	2.67	1.89	6.27E-66	4.8323E-67	-1.571242052	*haus3*
100330315	6.65	6.19	2.51	3.04	1.80E-82	1.12454E-83	-1.209287768	*adnpb*
337155	52.01	52.44	25.05	21.23	1.02E-286	1.834E-288	-1.186155315	*nap1l4a*
BGI_novel_G001456	2.03	2.44	0.86	1.17	5.40E-22	1.01207E-22	-1.141562793	*BGI_novel_G001456*
64274	0.73	0.89	0.44	0.31	6.86E-08	2.88551E-08	-1.097638458	*csf1ra*

Up-regulated DEGs related to cell cycle

Gene ID	control_1 FPKM	control_2 FPKM	epc1_mut_1 FPKM	epc1_mut_2 FPKM	Qvalue (control-vs-epc1_mut)	Pvalue (control-vs-epc1_mut)	log2 (epc1_mut/control)	Other Gene ID
368320	5.38	3.11	15.58	12.76	1.89543E-61	1.54E-62	1.726530125	*gmnn*
280649	18.48	21.9	64.43	65.14	0	0.00E+00	1.685601429	*plk1*
436938	1.55	1.74	4.82	4.97	4.844E-56	4.27E-57	1.576568757	*dmtf1*
393176	1.57	1.45	3.88	4.63	4.36226E-28	6.70E-29	1.49513824	*rad9a*
555606	2.75	1.63	5.58	5.86	7.54233E-42	8.39E-43	1.322491805	*bora*
563212	28.01	25.13	65.93	66.71	0	0.00E+00	1.322315842	*chaf1a*
571082	0.19	0.17	0.35	0.48	0.000736365	5.38E-04	1.240595468	*rad21l1*
30452	16.29	15.99	37.44	36.92	5.4745E-293	9.73E-295	1.206910739	*etv5b*
386844	16.88	16.98	36.11	38.11	4.9868E-246	1.09E-247	1.131216762	*ssrp1a*
554173	5.68	5.68	11.2	13.49	0	0.00E+00	1.125287075	*aspm*
100001603	0.77	1.29	2.39	1.78	1.45933E-17	3.27E-18	1.070670467	*brinp3a.2*
566857	4.12	4.73	8.29	9.93	6.01091E-46	6.14E-47	1.020940217	*poln*

Down-regulated DEGs related to cell cycle

**Gene ID**	control_1 FPKM	control_2 FPKM	epc1_mut_1 FPKM	epc1_mut_2 FPKM	Qvalue (control-vs-epc1_mut)	Pvalue (control-vs-epc1_mut)	log2 (epc1_mut/control)	Other Gene ID
436853	9.27	8.64	2.24	2.06	1.89956E-42	2.09E-43	-1.102941449	*pmepa1*
794952	5.85	6.56	2.38	3.32	8.8722E-26	1.46E-26	-1.116860711	*LOC794952*
324125	13.2	14.14	4.07	5.54	9.266E-155	3.31E-156	-1.50565518	*stk3*
192317	2.6	1.81	0.63	0.81	5.41163E-37	6.64E-38	-1.519769643	*stil*
406487	10.25	9.19	3.51	2.77	4.87431E-57	4.26E-58	-1.648695013	*rad51*
58216	0.72	0.91	0.26	0.22	5.81155E-05	3.49E-05	-1.736768336	*atoh7*
30188	3.52	4.56	1.23	1.15	4.49391E-41	5.07E-42	-1.758396871	*ccne1*
767751	13.34	14.89	3.55	3.72	8.5279E-133	3.50E-134	-1.952927365	*ccnb3*
110438505	1.87	1.37	0.46	0.25	5.4958E-06	2.85E-06	-2.186213601	*LOC110438505*
492707	6.51	5.4	1.22	1.28	3.9351E-138	1.54E-139	-2.249381948	*ncapg*
100330617	2.21	0	0.28	0	3.99386E-56	3.51E-57	-2.960296454	*tsc1b*
567533	1.94	2.12	0.28	0.17	1.44343E-81	9.13E-83	-3.185064798	*tnksb*
406455	109.6	110.18	10.88	11.96	0	0.00E+00	-3.262834738	*rcc2*
567674	2.01	2.17	0.01	0	6.0422E-163	2.08E-164	-8.585291539	*lats2*

Table S13 GO enrichment of DEGs related to acetylation in epc1a-/- embryos at 28 hpf , related to Figure 3

GO Term ID	GO Term	Level 1	Level 2	Term Candidate Gene Num	Total Candidate Gene Num	Term Gene Num	Total Gene Num	P value
GO:0006475	internal protein amino acid acetylation	biological_process	biological regulation	6	437	47	12665	0.005282581
GO:1902562	H4 histone acetyltransferase complex	cellular_component	organelle part	4	470	23	14111	0.006504612
GO:0006473	protein acetylation	biological_process	biological regulation	6	437	55	12665	0.0113312
GO:0031248	protein acetyltransferase complex	cellular_component	cell	6	470	58	14111	0.01239185
GO:1902493	acetyltransferase complex	cellular_component	macromolecular complex	6	470	58	14111	0.01239185
GO:0016573	histone acetylation	biological_process	cellular component organization or biogenesis	6	582	43	12665	0.01315512
GO:0018393	internal peptidyl-lysine acetylation	biological_process	cellular process	6	582	45	12665	0.01627635
GO:0018394	peptidyl-lysine acetylation	biological_process	cellular process	6	582	45	12665	0.01627635
GO:0018393	internal peptidyl-lysine acetylation	biological_process	biological regulation	5	437	45	12665	0.01878845
GO:0006475	internal protein amino acid acetylation	biological_process	cellular process	6	582	47	12665	0.01987418
GO:0000123	histone acetyltransferase complex	cellular_component	organelle part	5	470	49	14111	0.02297518
GO:0016407	acetyltransferase activity	molecular_function	catalytic activity	6	532	69	15078	0.03458191
GO:0043967	histone H4 acetylation	biological_process	biological regulation	3	437	22	12665	0.03861986

Table S14 GO enrichment of DEGs related to hematopoiesis and acetylation in ShEPC1, ShEPC2 and ShEPC1/2 K562 cells, related to Figure 5

Hematopoiesis/acetylation-related GO terms (ShEPC1 vs control)

Category	GOID	Description	GeneRatio	BgRatio	pvalue
BP	GO:0002283	neutrophil activation involved in immune response	79/1975	421/14136	0.00333645
BP	GO:0051249	regulation of lymphocyte activation	67/1975	333/14136	0.001128023
BP	GO:0042119	neutrophil activation	83/1975	429/14136	0.001084854
BP	GO:0036230	granulocyte activation	84/1975	433/14136	0.000934199
BP	GO:0046651	lymphocyte proliferation	46/1975	201/14136	0.000405243
BP	GO:0042110	T cell activation	74/1975	360/14136	0.000334315
BP	GO:0042098	T cell proliferation	36/1975	145/14136	0.000331187
BP	GO:0050870	positive regulation of T cell activation	41/1975	162/14136	8.41586E-05
BP	GO:0031063	regulation of histone deacetylation	8/1975	27/14136	0.027117693
BP	GO:0035065	regulation of histone acetylation	12/1975	49/14136	0.03413104
BP	GO:2000756	regulation of peptidyl-lysine acetylation	13/1975	55/14136	0.036832584
MF	GO:0016405	CoA-ligase activity	7/2029	23/14634	0.031637284

Hematopoiesis-related GO terms (ShEPC1 vs ShEPC2 vs ShEPC1/2)

Category	GOID	Description	GeneRatio	BgRatio	pvalue
BP	GO:0045577	regulation of B cell differentiation	3/262	27/18027	0.006859772
BP	GO:0061437	renal system vasculature development	3/262	27/18027	0.006859772
BP	GO:0061440	kidney vasculature development	3/262	27/18027	0.006859772
BP	GO:0090183	regulation of kidney development	4/262	57/18027	0.009426225
BP	GO:0001885	endothelial cell development	4/262	58/18027	0.010011416
BP	GO:0050869	negative regulation of B cell activation	3/262	31/18027	0.010102533
BP	GO:0050870	positive regulation of T cell activation	8/262	205/18027	0.010396039
BP	GO:0001886	endothelial cell morphogenesis	2/262	11/18027	0.010614315
BP	GO:0002328	pro-B cell differentiation	2/262	11/18027	0.010614315

Hematopoiesis-related GO terms (ShEPC1 vs ShEPC2 vs ShEPC1/2)

Category	GOID	Description	GeneRatio	BgRatio	pvalue
BP	GO:0001889	liver development	10/262	139/18027	3.6472E-05
BP	GO:0045446	endothelial cell differentiation	8/262	110/18027	0.000204265
BP	GO:0001822	kidney development	13/262	283/18027	0.000268863
BP	GO:0003158	endothelium development	8/262	128/18027	0.000569935
BP	GO:0060841	venous blood vessel development	3/262	15/18027	0.001213312
BP	GO:0072576	liver morphogenesis	3/262	20/18027	0.002881152
BP	GO:0090184	positive regulation of kidney development	4/262	41/18027	0.002894527
BP	GO:0007596	blood coagulation	12/262	339/18027	0.004148852
BP	GO:0072012	glomerulus vasculature development	3/262	25/18027	0.005510283
BP	GO:0030193	regulation of blood coagulation	5/262	79/18027	0.005870295
BP	GO:0030195	negative regulation of blood coagulation	4/262	52/18027	0.006834063
BP	GO:0045577	regulation of B cell differentiation	3/262	27/18027	0.006859772
BP	GO:0061437	renal system vasculature development	3/262	27/18027	0.006859772
BP	GO:0061440	kidney vasculature development	3/262	27/18027	0.006859772
BP	GO:0090183	regulation of kidney development	4/262	57/18027	0.009426225
BP	GO:0001885	endothelial cell development	4/262	58/18027	0.010011416
BP	GO:0050869	negative regulation of B cell activation	3/262	31/18027	0.010102533
BP	GO:0050870	positive regulation of T cell activation	8/262	205/18027	0.010396039
BP	GO:0001886	endothelial cell morphogenesis	2/262	11/18027	0.010614315
BP	GO:0002328	pro-B cell differentiation	2/262	11/18027	0.010614315

Table S15 GO enrichment of DEGs related to hematopoiesis and acetylation in ShEPC1, ShEPC2 and ShEPC1/2 K562 cells , related to Figure 5

Hematopoiesis/acetylation-related GO terms (ShEPC1 vs control)

Category	GOID	Description	GeneRatio	BgRatio	pvalue
BP	GO:0002444	myeloid leukocyte mediated immunity	90/1975	476/14136	0.00143428
BP	GO:0002573	myeloid leukocyte differentiation	39/1975	169/14136	0.000908997
BP	GO:0002763	positive regulation of myeloid leukocyte differentiation	15/1975	44/14136	0.00060356
BP	GO:0030218	erythrocyte differentiation	28/1975	108/14136	0.000703411
BP	GO:0002521	leukocyte differentiation	83/1975	421/14136	0.000602608
BP	GO:0034101	erythrocyte homeostasis	30/1975	116/14136	0.000483404
BP	GO:0002696	positive regulation of leukocyte activation	55/1975	251/14136	0.00037664
BP	GO:0048821	erythrocyte development	13/1975	33/14136	0.000276387
BP	GO:1903039	positive regulation of leukocyte cell-cell adhesion	42/1975	173/14136	0.000188131
BP	GO:1902107	positive regulation of leukocyte differentiation	33/1975	123/14136	0.000122531
BP	GO:0070661	leukocyte proliferation	53/1975	218/14136	2.79862E-05
BP	GO:0006085	acetyl-CoA biosynthetic process	10/1975	21/14136	0.000223681
BP	GO:0006084	acetyl-CoA metabolic process	13/1975	33/14136	0.000276387
BP	GO:0071616	acyl-CoA biosynthetic process	16/1975	50/14136	0.000886004
BP	GO:0006637	acyl-CoA metabolic process	22/1975	88/14136	0.004063209
BP	GO:0031065	positive regulation of histone deacetylation	7/1975	17/14136	0.005537677
BP	GO:0006476	protein deacetylation	21/1975	89/14136	0.00986981
BP	GO:0016575	histone deacetylation	18/1975	74/14136	0.011807318
BP	GO:0006086	acetyl-CoA biosynthetic process from pyruvate	6/1975	15/14136	0.01194107
BP	GO:0090312	positive regulation of protein deacetylation	7/1975	20/14136	0.015112618
BP	GO:0090311	regulation of protein deacetylation	11/1975	41/14136	0.022011902

Hematopoiesis/acetylation-related GO terms (ShEPC1 vs ShEPC2 vs ShEPC1/2)

Category	GOID	Description	GeneRatio	BgRatio	pvalue
BP	GO:0072576	liver morphogenesis	3/262	20/18027	0.002881152
BP	GO:0090184	positive regulation of kidney development	4/262	41/18027	0.002894527
BP	GO:0007596	blood coagulation	12/262	339/18027	0.004148852
BP	GO:0072012	glomerulus vasculature development	3/262	25/18027	0.005510283
BP	GO:0030193	regulation of blood coagulation	5/262	79/18027	0.005870295
BP	GO:0030195	negative regulation of blood coagulation	4/262	52/18027	0.006834063
BP	GO:0071616	acyl-CoA biosynthetic process	5/187	54/18027	0.000238497
BP	GO:0006637	acyl-CoA metabolic process	6/187	104/18027	0.000752249
BP	GO:0046949	fatty-acyl-CoA biosynthetic process	3/187	32/18027	0.004366776
BP	GO:0006084	acetyl-CoA metabolic process	3/187	38/18027	0.007097336
BP	GO:0035337	fatty-acyl-CoA metabolic process	3/187	41/18027	0.008767556
BP	GO:0035338	long-chain fatty-acyl-CoA biosynthetic process	2/187	19/18027	0.016299466
BP	GO:0035336	long-chain fatty-acyl-CoA metabolic process	2/187	25/18027	0.0274599

Table S16 Hematopoiesis and acetylation-related DEGs in ShEPC1, ShEPC2 and ShEPC1/2 K562 cells, related to Figure 5

Down-regulated DEGs related to hematopoiesis

gene_id	control-1_count	control-2_count	ShEPC1-1_count	ShEPC1-2_count	ShEPC2-1_count	ShEPC2-2_count	ShEPC1/2-1_count	ShEPC1/2-2_count	control-1_fpkm	control-2_fpkm	ShEPC1-1_fpkm	ShEPC1-2_fpkm	ShEPC2-1_fpkm	ShEPC2-2_fpkm	ShEPC1/2-1_fpkm	ShEPC1/2-2_fpkm	ShEPC1vscontrol_log2FoldChange	ShEPC1vscontrol_pvalue	ShEPC2vscontrol_log2FoldChange	ShEPC2vscontrol_pvalue	ShEPC1/2vscontrol_log2FoldChange	ShEPC1/2vscontrol_pvalue	gene_name
ENSG00000068878	5968	6203	6205	7729	4197	4013	5933	4872	27.8798	25.2046	29.1592	30.0408	19.0526	17.3591	21.4005	19.4476	0.163201	0.053904	-0.54455	8.69E-03	-0.36872	0.0079436	*PSME4*
ENSG00000177606	7443	9312	4398	5246	3827	2907	4742	4174	110.94	120.726	65.9433	65.0577	55.4312	40.1221	54.5748	53.1608	-0.81687	2.04E-23	-1.27904	2.32E-08	-1.095848	6.006E-16	*JUN*
ENSG00000198728	3463	3890	2770	3338	2576	2586	3822	3266	60.2571	58.8738	48.4852	48.3248	43.5567	41.6659	51.3493	48.5589	-0.29357	0.000745	-0.48375	1.92E-02	-0.244771	0.0758835	*LDB1*
ENSG00000151651	1112	1319	1004	1165	983	759	807	899	11.334	11.6934	10.2941	9.87946	9.73613	7.16338	6.35099	7.82954	-0.1857	0.115413	-0.44785	0.067827	-0.69041	0.0001231	*ADAM8*
ENSG00000184371	800	1032	504	611	585	647	669	640	7.41454	8.31938	4.69892	4.71155	5.2687	5.55259	4.7875	5.06841	-0.73636	2.37E-07	-0.54038	0.023308	-0.666533	0.0002609	*CSF1*
ENSG00000148400	473	481	304	341	318	169	339	276	2.15834	1.90907	1.39542	1.29462	1.41007	0.71407	1.19439	1.07613	-0.59099	0.001184	-0.94003	0.005285	-0.830893	0.0001804	*NOTCH1*

Up-regulated DEGs related to acetylation

gene_id	control-1_count	control-2_count	ShEPC1-1_count	ShEPC1-2_count	ShEPC2-1_count	ShEPC2-2_count	ShEPC1/2-1_count	ShEPC1/2-2_count	control-1_fpkm	control-2_fpkm	ShEPC1-1_fpkm	ShEPC1-2_fpkm	ShEPC2-1_fpkm	ShEPC2-2_fpkm	ShEPC1/2-1_fpkm	ShEPC1/2-2_fpkm	ShEPC1vscontrol_log2FoldChange	ShEPC1vscontrol_pvalue	ShEPC2vscontrol_log2FoldChange	ShEPC2vscontrol_pvalue	ShEPC1/2vscontrol_log2FoldChange	ShEPC1/2vscontrol_pvalue	gene_name
ENSG00000101040	2606	2928	3205	3893	3143	2506	4646	4059	16.5918	16.2147	20.5268	20.6221	19.4454	14.774	22.8396	22.0819	0.332689	0.000164	0.059603	0.792007	0.4625759	0.0007813	*ZMYND8*
ENSG00000112983	2148	2510	2748	3371	2470	2552	2971	2844	14.7996	15.0421	19.0462	19.3243	16.5374	16.2815	15.8055	16.7434	0.368379	5.51E-05	0.136757	0.512494	0.1344274	0.3503682	*BRD8*
ENSG00000146247	1356	1219	2237	2832	1652	1615	3328	2066	4.47271	3.49729	7.4225	7.77199	5.2951	4.93265	8.47584	5.82289	0.937732	3.68E-14	0.35951	0.122627	0.8526456	1.625E-05	*PHIP*
ENSG00000139618	170	146	397	436	304	432	615	425	0.64441	0.48137	1.51383	1.37508	1.1198	1.51633	1.80001	1.37657	1.367947	1.07E-09	1.229929	0.000142	1.5087491	8.67E-09	*BRCA2*
ENSG00000171843	86	131	315	371	477	610	298	384	0.51543	0.68291	1.89915	1.85002	2.77809	3.38533	1.37905	1.96654	1.647298	2.31E-11	2.360407	1E-14	1.4870764	1.41E-06	*MLLT3*

Down-regulated DEGs related to acetylation

gene_id	control-1_count	control-2_count	ShEPC1-1_count	ShEPC1-2_count	ShEPC2-1_count	ShEPC2-2_count	ShEPC1/2-1_count	ShEPC1/2-2_count	control-1_fpkm	control-2_fpkm	ShEPC1-1_fpkm	ShEPC1-2_fpkm	ShEPC2-1_fpkm	ShEPC2-2_fpkm	ShEPC1/2-1_fpkm	ShEPC1/2-2_fpkm	ShEPC1vscontrol_log2FoldChange	ShEPC1vscontrol_pvalue	ShEPC2vscontrol_log2FoldChange	ShEPC2vscontrol_pvalue	ShEPC1/2vscontrol_log2FoldChange	ShEPC1/2vscontrol_pvalue	gene_name
ENSG00000255302	2440	2853	1828	2322	2864	3664	3614	2734	58.0657	59.0539	43.7602	45.9748	66.2302	80.7387	66.4058	55.5937	-0.37826	7.4E-05	0.327762	1.34E-01	6.75E-02	0.655307	*EID1*
ENSG00000169057	1888	2015	1062	1315	1522	1566	1904	1252	4.99054	4.63273	2.82386	2.89201	3.90943	3.83296	3.88598	2.82779	-0.74538	8.09E-12	-0.31413	1.44E-01	-5.11E-01	0.0043615	*MECP2*
ENSG00000077684	1811	1942	1178	1349	1180	1159	1817	1688	9.71256	9.05902	6.35527	6.01943	6.14966	5.75567	7.52417	7.73544	-0.59566	5.55E-08	-0.65749	2.60E-03	-2.89E-01	0.055049	*JADE1*
ENSG00000147133	1059	1031	898	1210	685	247	1104	759	4.73397	4.00872	4.03812	4.50032	2.9756	1.02241	3.81055	2.89914	-0.02694	0.841247	-1.13244	0.002489	-0.372052	0.053347	*TAF1*
ENSG00000128342	55	52	16	20	38	29	60	26	0.68976	0.56723	0.20185	0.20869	0.4631	0.33677	0.581	0.27862	-1.6069	0.004885	-0.65312	0.25863	-0.535596	0.3424065	*LIF*

Table S17 Transcriptional activities of the DLST promoter by cotransfecting with EPC1 and EPC2, related to Figure 6

Name	Other name	Function	substrate specifity	Luciferase activity by cotransfecting with EPC1	Luciferase activity by cotransfecting with EPC2	Notes
KAT2A	*GCN5*	writer	H3(9, 14, 18)/H2B	ns	**up**	epigenetic transcription activation
KAT6A	*MOZ/MYST3*		H3(14)	ns	**up**	transcriptional activation, component of the MOZ/MORF complex, interacts with RUNX1
KAT7	*HBO1/MYST2*		H4(5, 8, 12)>H3	**up**	ns	part of the multimeric HBO1 complex
KAT8	*MYST1*		H4(16)	ns	ns	transcriptional activation, a member of the MYST histone acetylase protein family
KAT9	*ELP3*		H3	ns	ns	the catalytic subunit of the histone acetyltransferase elongator complex
KAT12	*GTF3C4*		H3	**up**	ns	histone acetyltransferase activity and enzyme activator activity
KAT13A	*NCOA1*		H3, H4	**up**	ns	participating in both chromatin remodeling and recruitment of general transcription factors
BRPF1		reader	H3(K9, 14, 23)	**up**	ns	component of the MOZ/MORF histone acetyltransferase complexes
CDYL				**up**	ns	transcriptional co-repressor
GFI1B		eraser	H3K56	ns	ns	transcriptional repressors, component of a RCOR-GFI-KDM1A-HDAC complex

Table S18 SRF or FOXR2 motifs in the hDLST/zdlst promoter region, related to Figure 7

Promoter	Matrix ID	Name	Score	Relative score	Strand	Predicted sequence
DLST	MA0083.2	MA0083.2.SRF	7.482153	0.808809356	+	CGACTCGTAAAAAGGCAA
DLST	UN0123.1	UN0123.1.FOXR2	13.380473	0.868434026	+	ACACAGATATAAAGGAT
DLST	UN0123.1	UN0123.1.FOXR2	9.390869	0.823912718	+	ATTAAAACATAAATTAA
dlst	MA0083.2	MA0083.2.SRF	16.01504	0.911541877	+	AGTGCCCTTAAAAGGTAA
dlst	UN0123.1	UN0123.1.FOXR2	20.851528	0.951805996	+	ACATAAATATAAATATT
dlst	UN0123.1	UN0123.1.FOXR2	19.029892	0.931477756	+	ATATATATATATATATA
dlst	UN0123.1	UN0123.1.FOXR2	19.029892	0.931477756	+	ATATATATATATATATA
dlst	UN0123.1	UN0123.1.FOXR2	19.029892	0.931477756	+	ATATATATATATATATA
dlst	UN0123.1	UN0123.1.FOXR2	19.029892	0.931477756	+	ATATATATATATATATA
dlst	UN0123.1	UN0123.1.FOXR2	19.029892	0.931477756	+	ATATATATATATATATA
dlst	UN0123.1	UN0123.1.FOXR2	19.029892	0.931477756	+	ATATATATATATATATA
dlst	UN0123.1	UN0123.1.FOXR2	19.029892	0.931477756	+	ATATATATATATATATA
dlst	UN0123.1	UN0123.1.FOXR2	19.029892	0.931477756	+	ATATATATATATATATA
dlst	UN0123.1	UN0123.1.FOXR2	19.029892	0.931477756	+	ATATATATATATATATA
dlst	UN0123.1	UN0123.1.FOXR2	19.029892	0.931477756	+	ATATATATATATATATA
dlst	UN0123.1	UN0123.1.FOXR2	19.029892	0.931477756	+	ATATATATATATATATA
dlst	UN0123.1	UN0123.1.FOXR2	19.029892	0.931477756	+	ATATATATATATATATA
dlst	UN0123.1	UN0123.1.FOXR2	19.029892	0.931477756	+	ATATATATATATATATA
dlst	UN0123.1	UN0123.1.FOXR2	19.029892	0.931477756	+	ATATATATATATATATA
dlst	UN0123.1	UN0123.1.FOXR2	19.029892	0.931477756	+	ATATATATATATATATA
dlst	UN0123.1	UN0123.1.FOXR2	19.029892	0.931477756	+	ATATATATATATATATA
dlst	UN0123.1	UN0123.1.FOXR2	19.029892	0.931477756	+	ATATATATATATATATA
dlst	UN0123.1	UN0123.1.FOXR2	19.029892	0.931477756	+	ATATATATATATATATA
dlst	UN0123.1	UN0123.1.FOXR2	19.029892	0.931477756	+	ATATATATATATATATA
dlst	UN0123.1	UN0123.1.FOXR2	19.029892	0.931477756	+	ATATATATATATATATA
dlst	UN0123.1	UN0123.1.FOXR2	19.029892	0.931477756	+	ATATATATATATATATA
dlst	UN0123.1	UN0123.1.FOXR2	19.029892	0.931477756	+	ATATATATATATATATA
dlst	UN0123.1	UN0123.1.FOXR2	19.029892	0.931477756	+	ATATATATATATATATA
dlst	UN0123.1	UN0123.1.FOXR2	19.029892	0.931477756	+	ATATATATATATATATA
dlst	UN0123.1	UN0123.1.FOXR2	19.029892	0.931477756	+	ATATATATATATATATA
dlst	UN0123.1	UN0123.1.FOXR2	19.029892	0.931477756	+	ATATATATATATATATA
dlst	UN0123.1	UN0123.1.FOXR2	19.029892	0.931477756	+	ATATATATATATATATA
dlst	UN0123.1	UN0123.1.FOXR2	19.029892	0.931477756	+	ATATATATATATATATA
dlst	UN0123.1	UN0123.1.FOXR2	19.029892	0.931477756	+	ATATATATATATATATA
dlst	UN0123.1	UN0123.1.FOXR2	19.029892	0.931477756	+	ATATATATATATATATA
dlst	UN0123.1	UN0123.1.FOXR2	19.029892	0.931477756	+	ATATATATATATATATA
dlst	UN0123.1	UN0123.1.FOXR2	19.029892	0.931477756	+	ATATATATATATATATA
dlst	UN0123.1	UN0123.1.FOXR2	19.029892	0.931477756	+	ATATATATATATATATA
dlst	UN0123.1	UN0123.1.FOXR2	19.029892	0.931477756	+	ATATATATATATATATA
dlst	UN0123.1	UN0123.1.FOXR2	19.029892	0.931477756	+	ATATATATATATATATA
dlst	UN0123.1	UN0123.1.FOXR2	19.029892	0.931477756	+	ATATATATATATATATA
dlst	UN0123.1	UN0123.1.FOXR2	19.029892	0.931477756	+	ATATATATATATATATA
dlst	UN0123.1	UN0123.1.FOXR2	19.029892	0.931477756	+	ATATATATATATATATA
dlst	UN0123.1	UN0123.1.FOXR2	19.029892	0.931477756	+	ATATATATATATATATA
dlst	UN0123.1	UN0123.1.FOXR2	19.029892	0.931477756	+	ATATATATATATATATA
dlst	UN0123.1	UN0123.1.FOXR2	19.029892	0.931477756	+	ATATATATATATATATA
dlst	UN0123.1	UN0123.1.FOXR2	19.029892	0.931477756	+	ATATATATATATATATA
dlst	UN0123.1	UN0123.1.FOXR2	19.029892	0.931477756	+	ATATATATATATATATA
dlst	UN0123.1	UN0123.1.FOXR2	19.029892	0.931477756	+	ATATATATATATATATA
dlst	UN0123.1	UN0123.1.FOXR2	19.029892	0.931477756	+	ATATATATATATATATA
dlst	UN0123.1	UN0123.1.FOXR2	19.029892	0.931477756	+	ATATATATATATATATA
dlst	UN0123.1	UN0123.1.FOXR2	19.029892	0.931477756	+	ATATATATATATATATA
dlst	UN0123.1	UN0123.1.FOXR2	19.029892	0.931477756	+	ATATATATATATATATA
dlst	UN0123.1	UN0123.1.FOXR2	19.029892	0.931477756	+	ATATATATATATATATA
dlst	UN0123.1	UN0123.1.FOXR2	19.029892	0.931477756	+	ATATATATATATATATA
dlst	UN0123.1	UN0123.1.FOXR2	19.029892	0.931477756	+	ATATATATATATATATA
dlst	UN0123.1	UN0123.1.FOXR2	19.029892	0.931477756	+	ATATATATATATATATA
dlst	UN0123.1	UN0123.1.FOXR2	19.029892	0.931477756	+	ATATATATATATATATA
dlst	UN0123.1	UN0123.1.FOXR2	19.029892	0.931477756	+	ATATATATATATATATA
dlst	UN0123.1	UN0123.1.FOXR2	19.029892	0.931477756	+	ATATATATATATATATA

dlst	UN0123.1	UN0123.1.FOXR2	19.029892	0.931477756	+	ATATATATATATATATA
dlst	UN0123.1	UN0123.1.FOXR2	19.029892	0.931477756	+	ATATATATATATATATA
dlst	UN0123.1	UN0123.1.FOXR2	19.029892	0.931477756	+	ATATATATATATATATA
dlst	UN0123.1	UN0123.1.FOXR2	19.029892	0.931477756	+	ATATATATATATATATA
dlst	UN0123.1	UN0123.1.FOXR2	19.029892	0.931477756	+	ATATATATATATATATA
dlst	UN0123.1	UN0123.1.FOXR2	19.029892	0.931477756	+	ATATATATATATATATA
dlst	UN0123.1	UN0123.1.FOXR2	19.029892	0.931477756	+	ATATATATATATATATA
dlst	UN0123.1	UN0123.1.FOXR2	19.029892	0.931477756	+	ATATATATATATATATA
dlst	UN0123.1	UN0123.1.FOXR2	19.029892	0.931477756	+	ATATATATATATATATA
dlst	UN0123.1	UN0123.1.FOXR2	19.029892	0.931477756	+	ATATATATATATATATA
dlst	UN0123.1	UN0123.1.FOXR2	19.029892	0.931477756	+	ATATATATATATATATA
dlst	UN0123.1	UN0123.1.FOXR2	19.029892	0.931477756	+	ATATATATATATATATA
dlst	UN0123.1	UN0123.1.FOXR2	19.029892	0.931477756	+	ATATATATATATATATA
dlst	UN0123.1	UN0123.1.FOXR2	19.029892	0.931477756	+	ATATATATATATATATA
dlst	UN0123.1	UN0123.1.FOXR2	19.029892	0.931477756	+	ATATATATATATATATA
dlst	UN0123.1	UN0123.1.FOXR2	19.029892	0.931477756	+	ATATATATATATATATA
dlst	UN0123.1	UN0123.1.FOXR2	19.029892	0.931477756	+	ATATATATATATATATA
dlst	UN0123.1	UN0123.1.FOXR2	19.029892	0.931477756	+	ATATATATATATATATA
dlst	UN0123.1	UN0123.1.FOXR2	19.029892	0.931477756	+	ATATATATATATATATA
dlst	UN0123.1	UN0123.1.FOXR2	19.029892	0.931477756	+	ATATATATATATATATA
dlst	UN0123.1	UN0123.1.FOXR2	19.029892	0.931477756	+	ATATATATATATATATA
dlst	UN0123.1	UN0123.1.FOXR2	19.029892	0.931477756	+	ATATATATATATATATA
dlst	UN0123.1	UN0123.1.FOXR2	19.029892	0.931477756	+	ATATATATATATATATA
dlst	UN0123.1	UN0123.1.FOXR2	19.029892	0.931477756	+	ATATATATATATATATA
dlst	UN0123.1	UN0123.1.FOXR2	19.029892	0.931477756	+	ATATATATATATATATA
dlst	UN0123.1	UN0123.1.FOXR2	19.029892	0.931477756	+	ATATATATATATATATA
dlst	UN0123.1	UN0123.1.FOXR2	19.029892	0.931477756	+	ATATATATATATATATA
dlst	UN0123.1	UN0123.1.FOXR2	19.029892	0.931477756	+	ATATATATATATATATA
dlst	UN0123.1	UN0123.1.FOXR2	19.029892	0.931477756	+	ATATATATATATATATA
dlst	UN0123.1	UN0123.1.FOXR2	19.029892	0.931477756	+	ATATATATATATATATA
dlst	UN0123.1	UN0123.1.FOXR2	18.575104	0.926402624	+	ACATATATATATATATA
dlst	UN0123.1	UN0123.1.FOXR2	18.399237	0.924440066	+	ATATATATATATATAAT
dlst	UN0123.1	UN0123.1.FOXR2	18.045307	0.920490449	+	ATATATATATATATACA
dlst	UN0123.1	UN0123.1.FOXR2	17.620934	0.915754724	+	ATATATATATACATAAA
dlst	UN0123.1	UN0123.1.FOXR2	16.92714	0.908012445	+	ATAAATACATAAAAAAT
dlst	UN0123.1	UN0123.1.FOXR2	16.37998	0.901906515	+	ACACATATATATATATA
dlst	UN0123.1	UN0123.1.FOXR2	16.345362	0.901520197	+	TTATATATATATATATA
dlst	UN0123.1	UN0123.1.FOXR2	16.07648	0.898519667	+	ATATATATATATACATA
dlst	UN0123.1	UN0123.1.FOXR2	15.957517	0.897192106	+	CTATATATATATATATA
dlst	UN0123.1	UN0123.1.FOXR2	15.824302	0.895705516	+	ACACACACATATATATA
dlst	UN0123.1	UN0123.1.FOXR2	15.214537	0.888900947	+	ATATATATATATATAGT
dlst	UN0123.1	UN0123.1.FOXR2	13.884218	0.874055484	+	AACTATATATATATATA
dlst	UN0123.1	UN0123.1.FOXR2	13.674029	0.871709917	+	ATATATACACACATAAA
dlst	UN0123.1	UN0123.1.FOXR2	13.629248	0.871210183	+	ATATATATATACACACA
dlst	UN0123.1	UN0123.1.FOXR2	13.313339	0.867684857	+	ATATATATATAATTACA
dlst	UN0123.1	UN0123.1.FOXR2	12.698407	0.860822627	+	ACACACATATATATATA
dlst	UN0123.1	UN0123.1.FOXR2	12.255239	0.855877162	+	ATATATATATATAATTA
dlst	UN0123.1	UN0123.1.FOXR2	11.557459	0.848090408	+	ATACACACATAAAATAA
dlst	UN0123.1	UN0123.1.FOXR2	10.997122	0.841837422	+	AAGTAAACAAATAAAAT
dlst	UN0123.1	UN0123.1.FOXR2	10.496305	0.836248647	+	ATATAAATATTTACATA
dlst	UN0123.1	UN0123.1.FOXR2	10.252442	0.833527298	+	ATATATATATATAGTTT
dlst	UN0123.1	UN0123.1.FOXR2	10.166588	0.832569219	+	TAATATATATATACACA
dlst	UN0123.1	UN0123.1.FOXR2	9.860025	0.829148188	+	AAATAAATAAATAAATA
dlst	UN0123.1	UN0123.1.FOXR2	9.708251	0.827454487	+	AAATAATTATACATAAA
dlst	UN0123.1	UN0123.1.FOXR2	9.335601	0.823295961	+	ATATACACACATAAAAT
dlst	UN0123.1	UN0123.1.FOXR2	9.101108	0.820679173	+	TTAATTACATAAATATA
dlst	UN0123.1	UN0123.1.FOXR2	8.826871	0.817618876	+	TATTATATATATATATA
dlst	UN0123.1	UN0123.1.FOXR2	8.789143	0.817197853	+	ATAAATAAATAAATAGA
dlst	UN0123.1	UN0123.1.FOXR2	8.768173	0.816963849	+	TTACATAAATATAAATA
dlst	UN0123.1	UN0123.1.FOXR2	8.751502	0.816777809	+	ACACACACACATATATA
dlst	UN0123.1	UN0123.1.FOXR2	8.728912	0.816525724	+	ATATATATACATAAATG
dlst	UN0123.1	UN0123.1.FOXR2	8.122644	0.80976018	+	AATTATACATAAAATAG
dlst	UN0123.1	UN0123.1.FOXR2	7.9659166	0.808011202	+	AACTACACATAATTAAT
dlst	UN0123.1	UN0123.1.FOXR2	7.556989	0.803447846	+	AAATAAATAAATAGACA

dlst	UN0123.1	UN0123.1.FOXR2	7.541033	0.803269783	+	ATATATATACACACATA
dlst	UN0123.1	UN0123.1.FOXR2	7.288854	0.800455638	+	ACACACACACACATATA

Table S19 Effective target screening for SRF/FoxR2 binding site in zebrafish *dlst* promoter, related to Figure 7

gRNA name	Target site	Sequence (5'to3')	Efficiency
gRNA-1	targeting SRF binding site	TGACTGTTATTTACCTTTTA**AGG**	No
gRNA-2	targeting SRF binding site	ATATTTAAAGTGCCCTTAAA**AGG**	Yes
gRNA-3	targeting upstream sequence of SRF binding site	AAATGATGTTTAACAGAGCA**AGG**	No
gRNA-4	targeting downstream sequence of SRF binding site	GCTAGCAGACACAATCCTAA**GGG**	No
gRNA-5	targeting downstream sequence of SRF binding site	GTCAATAGGCAGTGTATTTA**GGG**	No
gRNA-6	targeting upstream sequence of FOXR2 binding site 1	TTTCATTAACAGTAGTAACA**GGG**	No
gRNA-7	targeting upstream sequence of FOXR2 binding site 1	TACTGTTAATGAAATTAGAA**TGG**	No
gRNA-8	targeting upstream sequence of FOXR2 binding site 1	ACTGTTAATGAAATTAGAAT**GGG**	No
gRNA-9	targeting downstream sequence of FOXR2 binding site 1 or targeting upstream sequence of FOXR2 binding site 2	ATCAAGAATGTACTTACTTT**TGG**	No
gRNA-10	targeting downstream sequence of FOXR2 binding site 2	AGCACACGTCGGAAGCAAAG**CGG**	No
gRNA-11	targeting downstream sequence of FOXR2 binding site 2	CAGCTCTCACGGCACAGCAG**CGG**	No
gRNA-12	targeting downstream sequence of FOXR2 binding site 2	GAAACAGCGCGAGCACACGT**CGG**	No
gRNA-13	targeting downstream sequence of FOXR2 binding site 2	CCCCCCGCTCCACCTCATGA**TGG**	No
