# Supplementary figures and images for: EPC1/2 regulate hematopoietic stem and progenitor cell proliferation by modulating H3 acetylation and DLST
Source: iScience. 2024 Feb 17;27(3):109263. doi: 10.1016/j.isci.2024.109263 (PMC10910311; doi:10.1016/j.isci.2024.109263)

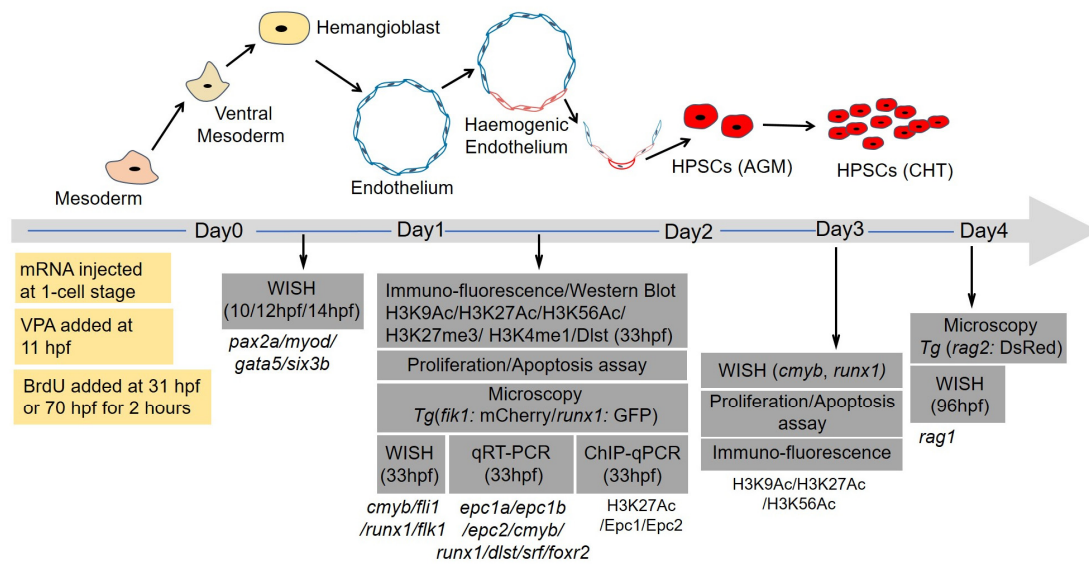

**Schema 1**

**Schema 1. The schema of the experimental program in zebrafish, related to all figures**

Supplement: Schema 1. The schema of the experimental program in zebrafish, related to all figures [file mmc2.pdf]
